# Revisiting 2‑Substituted-4(1*H*)‑Quinolones for Targeting the *Plasmodium falciparum* Cytochrome bc_1_ Complex

**DOI:** 10.1021/acs.jmedchem.5c03295

**Published:** 2026-06-10

**Authors:** Sovitj Pou, Katherine M. Liebman, Rolf W. Winter, Aaron Nilsen, Yuexin Li, Isaiah N. Lyche, Teresa M. Riscoe, Jemma Montgomery, Max J. Gramelspacher, Rozalia A. Dodean, Lev N. Zakharov, Binod Nepal, Jane X. Kelly, Martin J. Smilkstein, Sandhya Kortagere, Akhil B. Vaidya, P. Holland Alday, J. Stone Doggett, Karl Kudyba, Norma Roncal, Kutub Ashraf, Susan Leed, Patricia J. Lee, Michael S. Madejczyk, Alison Roth, Michael K. Riscoe

**Affiliations:** † VA Portland Healthcare System, 3710 SW US Veterans Hospital Road, Portland, Oregon 97239, United States; ‡ Department of Chemical Physiology and Biochemistry, 6684 Oregon Health & Science University, 3181 SW Sam Jackson Park Road, Portland, Oregon 97239, United States; § Department of Microbiology and Molecular Immunology, Oregon Health & Science University, 3181 SW Sam Jackson Park Road, Portland, Oregon 97239, United States; ∥ Center for Advanced Materials Characterization in Oregon (CAMCOR), Eugene, Oregon 97403, United States; ⊥ Department of Microbiology and Immunology, 12312 Drexel University College of Medicine, 2900 Queen Lane, Philadelphia, Pennsylvania 19129, United States; # School of Medicine Division of Infectious Diseases, Oregon Health & Science University, 3181 SW Sam Jackson Park Road, Portland, Oregon 97239, United States; ¶ 8394 Experimental Therapeutics Branch, CIDR, Walter Reed Army Institute of Research, Silver Spring, Maryland 20910, United States; ∇ Integrated Pathogen Therapeutics Department, CIDR, Walter Reed Army Institute of Research, Silver Spring, Maryland 20910, United States

## Abstract

Quinolones substituted at the 2- and 3-positions with biaryl and diphenylether groups have been investigated for their antimalarial potential. ELQ-300, with a 3-position diphenyl ether, is at an advanced stage of preclinical development. Here, we synthesize the 2-position isomer of ELQ-300, i.e., HLQ-102, and describe synthetic procedures for preparing it that avoid the use of expensive catalysts and afford access to substituted quinolones bearing substituents in the 2-position as well as the benzenoid ring and with the critical 3-position CH_3_ group. We profile HLQ analogs for their antimalarial activity along with pharmacokinetics of the selected lead molecule. Cross-resistance patterns indicate that, like its predecessor, HLQ-102 targets the Q_i_ site of the parasite cytochrome bc_1_ complex. This finding suggests the existence of two separate troughs in the target protein capable of accommodating such large structural features regardless of whether it is placed at the 2- or 3-positon of the quinolone ring.

## Graphical abstract

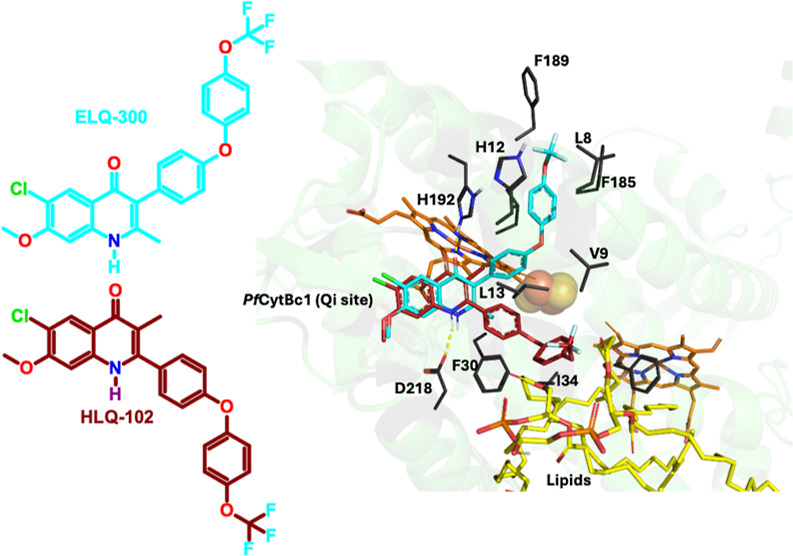

## Introduction

Malaria, as a major infectious disease, remains one of the leading causes of death and morbidity around the world despite determined efforts to blunt transmission with insecticides, insecticide-treated bed nets and treatment of active infections with antimalarial drugs.^1^ Anopheline mosquitoes that transmit malaria have become resistant to insecticides,^2,3^ and *Plasmodium falciparum*, which causes potentially lethal malaria infections, has acquired stable resistance to most of the antimalarials in clinical use today.^4^ As a result, there is a critical need for development of new countermeasures.

**Table 1:**
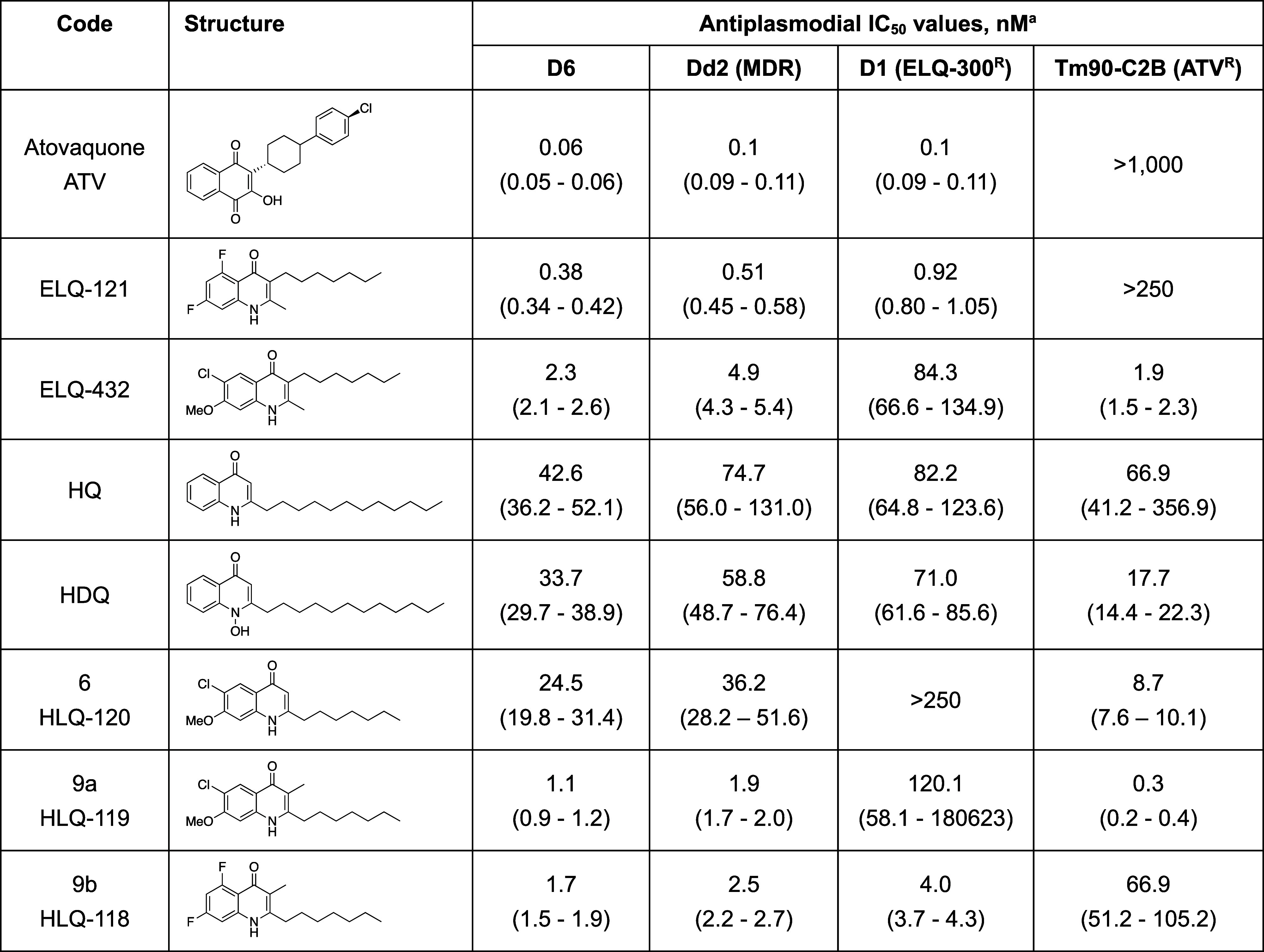
Comparative IC_50_ Values for Select ELQs and HLQ Derivatives Where the Extended Hydrophobic Alkyl Group is at the 2- or 3-Position^a^

a IC_50_ values represent the concentration of test agent that suppresses parasite growth by 50% relative to controls without addition of compound. For mean IC_50_ values, minimally quadruplicate data were collected for each concentration in the dose–response curves, and error represents the 95% confidence interval of the fit. For each compound, we show the results from a single representative experiment.

**Table 2:**
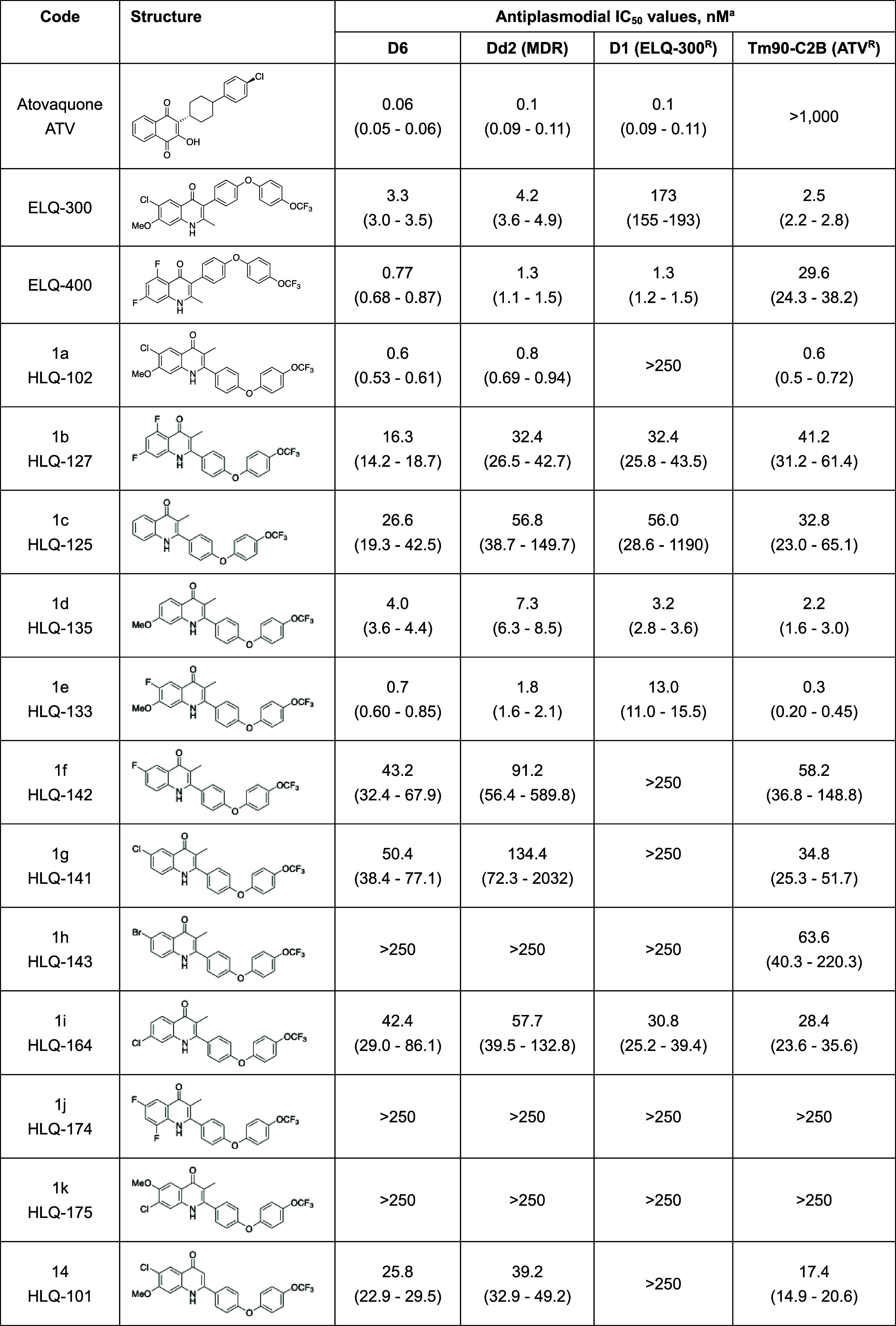
Comparative IC_50_ Values for Select ELQs and HLQ Derivatives Where a Diphenyl Ether Group is at the 2- or 3-Position^a^

a IC_50_ values represent the concentration of compound that suppresses parasite growth by 50% relative to controls without addition of drug. For mean IC_50_ values, minimally quadruplicate data were collected for each concentration in the dose–response curves, and error represents the 95% confidence interval of the fit. For each test agent, we show the results from a single representative experiment.

Over 20 years ago we became interested in endochin (Figure 1), a historical drug first described by Hans Andersag and colleagues working at Bayer IG Farbenindustrie in Elberfeld, Germany.^5^ We joined a consortium of investigators brought together by the Medicines for Malaria Venture (MMV) to optimize endochin for human use, and this effort yielded ELQ-300 (Figure 1), which remains listed in MMV’s preclinical development pipeline.^6,7^ It has been shown that ELQ-300 selectively inhibits the parasite cytochrome bc_1_ complex, targeting the Q_i_ site of the *P. falciparum* enzyme.^8^ This contrasts with atovaquone (Figure 1), which inhibits the distant Q_o_ site.^9,10^ Both agents are known to kill malaria parasites at all three of its life cycle stages (liver, blood, and vector stages), and there is a rational basis for combining the two drugs in a single antimalarial coformulation to improve outcomes and diminish the likelihood for resistance.^11^ Despite the success of ELQ-300 we continue to explore the ELQ scaffold for improvements in potency, e.g., ELQ-596 (Figure 1), and physiochemical characteristics such as decreased crystallinity and increased aqueous solubility.

**Figure 1:**
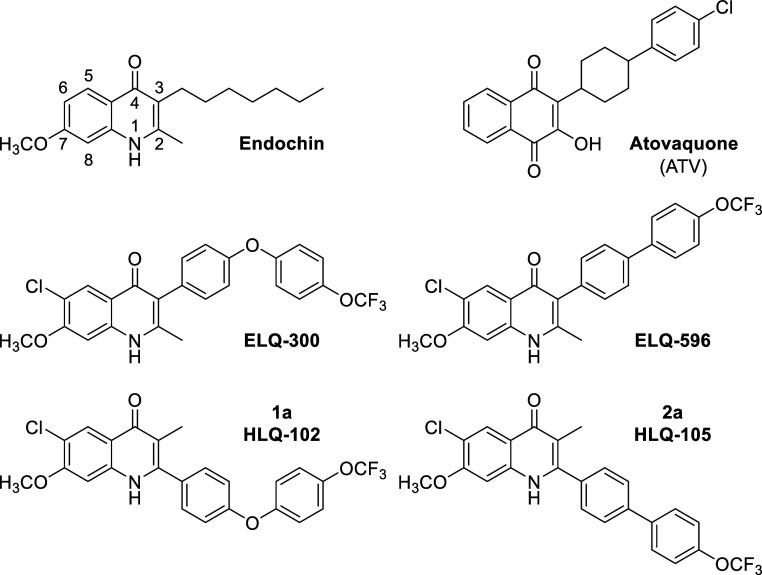
Chemical structure of the clinical drug atovaquone along with the historical lead, endochin, and advanced preclinical candidates **ELQ-300** and **ELQ-596**. These drugs target the cytochrome *bc*_1_ complex of *P. falciparum*. HLQ-102 and HLQ-105 are also presented.

While endochin served as the historical lead guiding our laboratory’s pursuit of endochin-like quinolones (ELQs) with 3-position side chains comprised of substituted diphenyl ether and biaryl groups,^6,12^ 1-hydroxy-2-dodecyl-4­(1*H*)-quinolone (HDQ) guided Stephen Ward’s group to study 2-position substituted quinolones (Figure 2).^13^ In a series of papers, they describe analogs with low nM IC_50_’s that target complex I and complex III of the *P. falciparum* electron transport chain.^13–18^ Their investigation produced molecules that targeted the Q_o_ and Q_i_ sites of *P. falciparum* cyt. *bc*_1_ including CK-2–68.^19^ RYL-552 (Figure 2) is a structurally similar molecule produced by the laboratory of Dr. Yu Rao.^20^ Sequencing of mutants resistant to these two compounds has demonstrated that they target the Q_o_ site of *P. falciparum* cyt. *bc*_1_.^21^ In a retrospective analysis of our ELQ chemical collection of ∼1000 molecules and after a review of the published literature, we realized that the 2-position isomers of ELQ-300 and ELQ-596 had not been prepared and examined alongside these lead molecules for comparison purposes. In this paper we describe new methods for the synthesis of these molecules, HLQ-102 (**1a**) and HLQ-105 (**2a**) (Figure 1), along with an assessment of critical physiochemical parameters, antimalarial profiling in vitro and in vivo, and pharmacological testing in preclinical species. We then expanded this series to gain an understanding of the associated structure–activity relationship (SAR) profile with targeting of the Q_o_ and Q_i_ sites of cytochrome bc_1_ complex. Our findings indicate that selected HLQs are highly active molecules with improved solubility and crystallinity characteristics and deserving of further examination.

**Figure 2:**
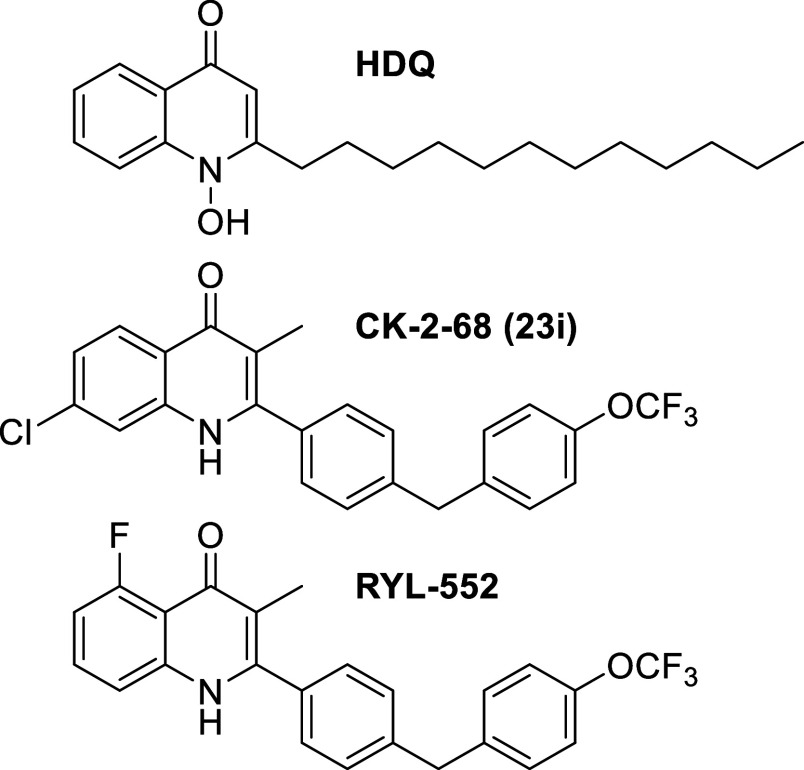
Structure of HDQ and advanced 2-substituted quinolones produced by Dr. Ward’s group (CK-2–68) and Dr. Rao’s group (RYL-552), respectively.

## Results

A number of synthetic approaches have been developed for the synthesis of 2-substituted 4­(1*H*)-quinolones.^22^ De Souza et al. used the Camps^23,24^ method (Scheme 1a) to synthesize a series of 2-aryl 4­(1*H*)-quinolones.^25^ The Gould-Jacobs^26^ method (Scheme 1b) has also been used to prepare 2-substituted 4­(1*H*)-quinolones. Unfortunately, these methods do not allow the preparation of 2,3-substituted 4­(1*H*)-quinolones (R_2_ must be hydrogen). Ward and O’Neill developed several methods that overcame this limitation including 4­(1*H*)-quinolone-forming cyclizations that involved oxazoline^16,18^ (Scheme 1c) or carboxylic acid^18^ (Scheme 1d) intermediates and a method that required a selective Suzuki reaction^16^ (Scheme 1e). Unfortunately, these cyclization methods limited the available choices of benzenoid substituents X, because: (1) trisubstituted benzenes are available with a limited set of substituents, and (2) tetra-substituted benzenes that would be required for two benzenoid substituents X are essentially unavailable. The scope of the Suzuki reaction method was not explored, even though it would have allowed for both 3-position substitution and multiple benzenoid substituents X. The Rao lab used both the oxazoline and Conrad–Limpach^27,28^ (Scheme 1f) methods to prepare a series of 2-substituted 4­(1*H*)-quinolones.^20,29,30^ HDQ analogs, 2-alkyl substituted 4­(1*H*)-quinolones, prepared using the Conrad–Limpach approach^31–34^ exhibited diverse biological activities, including antimicrobial,^33^ *Pseudomonas* quorum-sensing (PQS) inhibition,^32^ and antibacterial activity against bacteria such as *Neisseria gonorrheae*.^34^ Unfortunately, though the Conrad–Limpach approach methods involved a synthetically tractable β-ketoester, none of them were adapted to produce 2,3-substituted 4­(1*H*)-quinolones. Here we present a new Conrad–Limpach approach, involving an α-carbon substituted (R_2_) β-ketoester, that provides access to 2,3-disubstiuted 4­(1*H*)-quinolones where R_1_ is alkyl or aryl and R_2_ is alkyl. Because simple X-substituted anilines are used, this approach also provides access to a wide range of benzenoid substituents X.

**Scheme 1:**
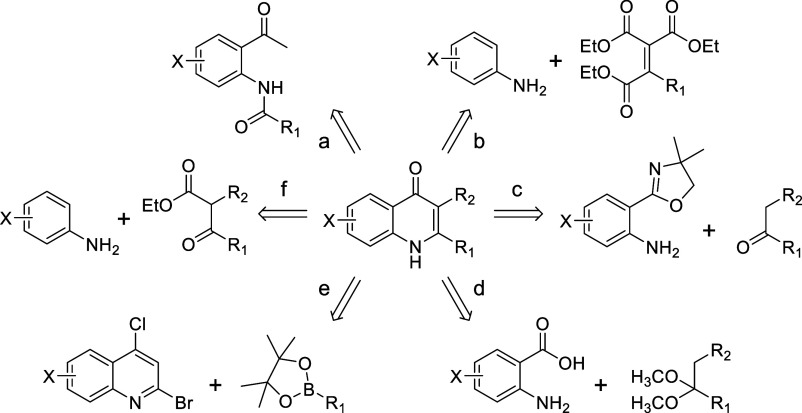
Methods for the Preparation of 2-Substituted 4­(1*H*)-Quinolones

The synthesis of **6** (HLQ-120) was accomplished in 36% yield by condensing ethyl 3-oxodecanoate **4** ^31^ with 4-chloro-6-methoxyaniline **3a** under Dean–Stark conditions in refluxing benzene to form the corresponding Schiff base, which was subsequently cyclized thermally in boiling Dowtherm A via the Conrad–Limpach reaction (Scheme 2). For **9a** (HLQ-119) and **9b** (HLQ-118), the introduction of a methyl group at the 3-position was required. Following an analogous synthetic approach,^35^ ethyl 2-methyl-3-oxodecanoate **7** was first prepared and then condensed with the appropriate aniline (**3a** or **3b**), followed by the Conrad–Limpach reaction to afford the desired products in 19–26% yield. For compound **9a**, a prodrug intermediate was prepared to facilitate purification, which was subsequently hydrolyzed to yield pure **9a** (see Supporting Information).

**Scheme 2:**
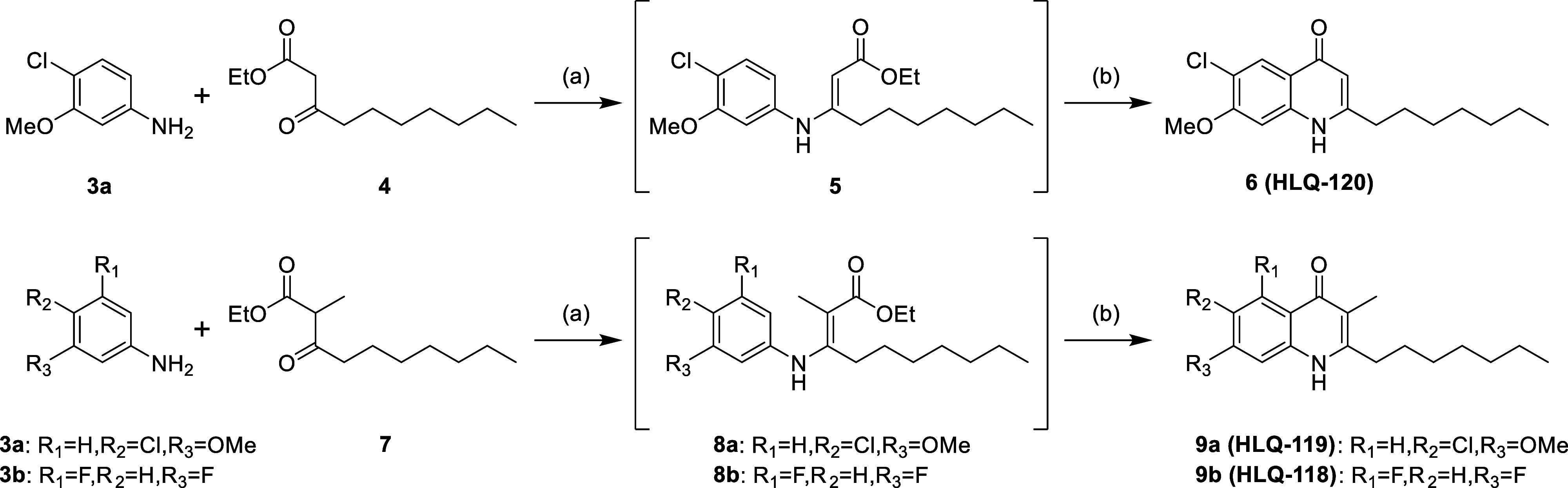
Synthesis of a Series of 2-Alkyl-Quinolones with Variable Substitution of the Benzenoid Ring (R_1_, R_2_, R_3_) from Anilines **3a** and **3b** ^a^

a Reactions (a): catalytic *p-*TsOH·H_2_O, benzene, reflux 22 h-19 days; (b): Dowtherm A, 250 °C, 2–10 min, 19–26%.

The synthesis of the diaryl ether **11a** has been previously reported^36^ using copper iodide and picolinic acid as a catalyst. In our study, we found that **11a** could be synthesized without the need of a catalyst by coupling the activated 4-fluoro acetophenone with the 4-trifluoromethoxy phenol in the presence of potassium carbonate (K_2_CO_3_) in dimethylformamide (DMF) at 125–130 °C for 19 h (Scheme 2). Following a similar reported procedure,^36^ the β-keto ester **12a** ^18^ was obtained as a crude product that was used without further purification. The desired quinolone **14** (**HLQ-101**) was then synthesized via the Conrad–Limpach reaction of the corresponding Schiff base **13** in 42% yield.

To obtain 3-methyl substituted quinolones bearing various substituents on the benzenoid ring, the key intermediate β -keto ester **12b** was required (Scheme 3). Interestingly, **12b** has not been previously reported. Although the preparation of the corresponding diaryl ether **11b** using copper acetate has been described,^18^ we successfully prepared **11b** by coupling 4-fluoropropiophenone **10b** with 4-trifluoromethoxy phenol in 63% yield. Using a similar procedure to that described above, the key intermediate **12b** was obtained in 42% yield. The desired quinolones **1a**–**1k** were then synthesized via Conrad–Limpach reactions of the corresponding Schiff bases **15a**–**15k** in 1–57% yield. Unlike the preparations of Schiff bases for the synthesis of 3-substituted quinolones, which typically proceed within 1–3 days,^37^ the formation of the Schiff bases **15a**–**15k** was notably slower, requiring 3–21 days (see Supporting Information). Moreover, monitoring reaction progress proved challenging, as conventional techniques such as gas chromatography–mass spectrometry (GC–MS), thin-layer chromatography (TLC), and observation of azeotropic water formation in the Dean–Stark apparatus were not reliable indicators. In practice, we found that using a minimal amount of benzene and allowing the reaction to proceed for approximately 3 days generally provided a good yield of intermediate for the subsequent Conrad–Limpach cyclization. Compounds **1f**, **1h**, **1i** and **1k** could not be obtained in pure form after the Conrad–Limpach reaction. Pivalate-protected intermediates were prepared to facilitate purification, which were then subsequently hydrolyzed to yield pure products (see Supporting Information).

**Scheme 3:**
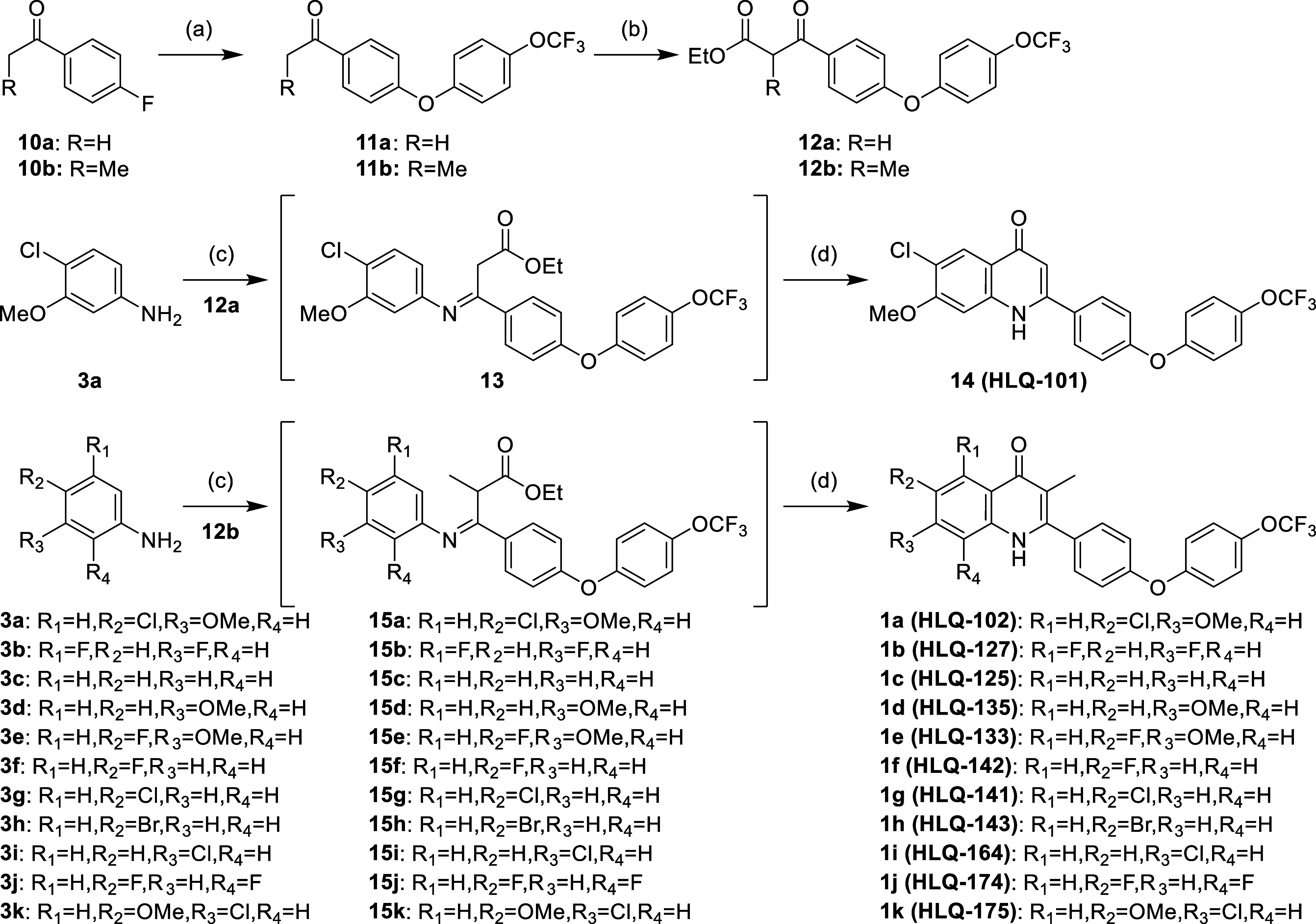
Synthesis of a Series of 2-Diaryl Ether-Quinolones with Variable Substitution of the Benzenoid Ring (R_1_, R_2_, R_3_ and R_4_) from β-Ketoesters **12a** and **12b** ^a^

a Reactions (a): 4-trifluoromethoxy phenol, K_2_CO_3_, DMF, 140 °C, 72 h, 50–63%; (b): 60% oil suspension NaH, diethyl carbonate, THF, 42%; (c): catalytic *p-*TsOH·H_2_O, benzene, reflux 3–14 days; (d): Dowtherm A, 250 °C, 2–10 min, 1–57% over two steps.

For the synthesis of 2-substituted diphenylmethane and biphenyl quinolones, we followed our previously reported strategy^37^ beginning with the preparation of the intermediate 2-(4-bromophenyl)-4-chloroquinolines **20**, **23a**–**23e** and **23i** (Scheme 4). These were then subjected to palladium-catalyzed Suzuki reactions with various boronic acids or esters to provide 2-diphenylmethane- and 2-biphenyl-4-chloro quinolines **24a**–**24c**, **24e**, **24i**, **26**, **28a**–**28e**, **28i** and **29**, followed by hydrolysis to yield the target compounds **2a**–**2e**, **2i**, **25a**–**25c**, **25e**, **25i**, **27** and **30** (Scheme 5).

**Scheme 4:**
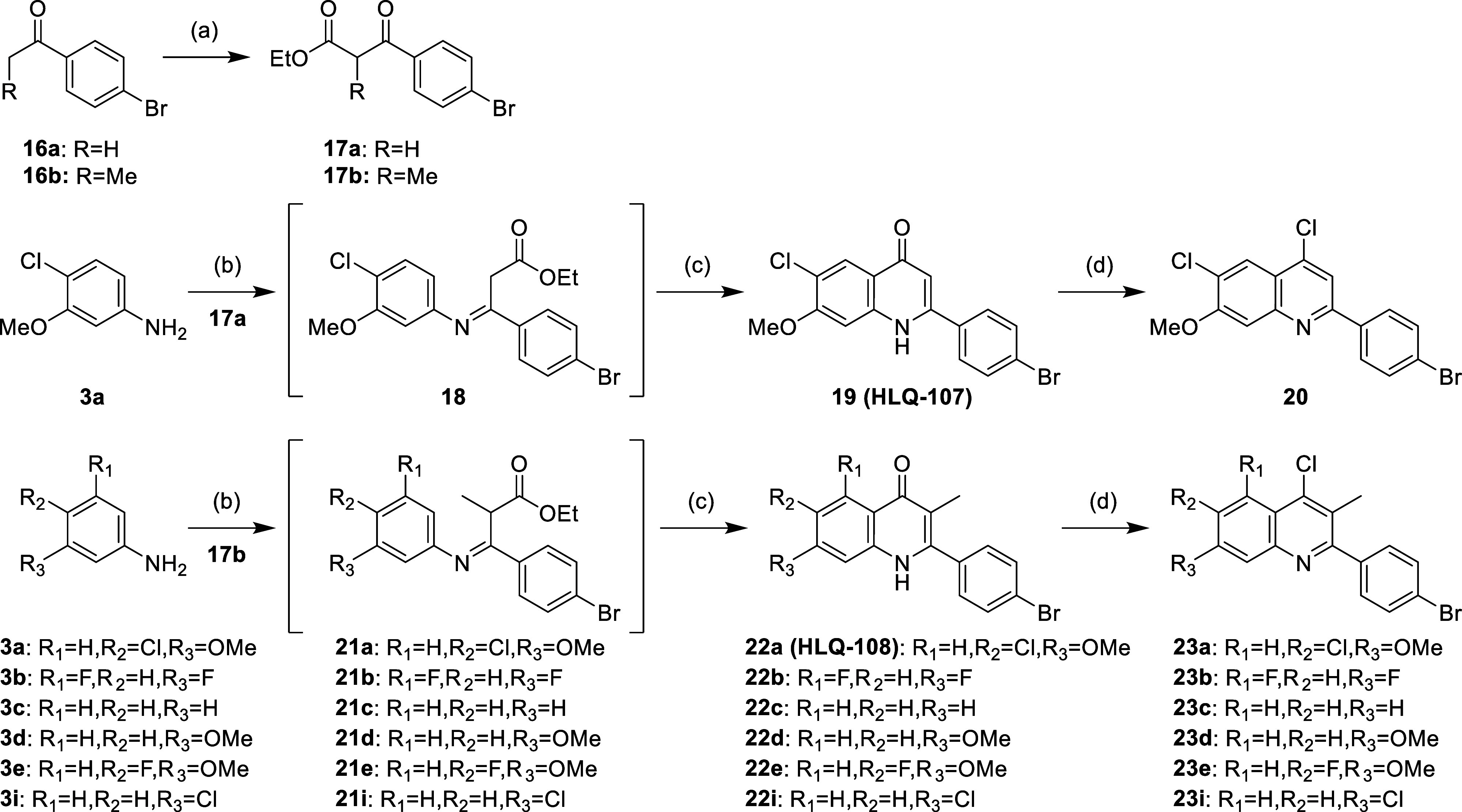
Synthesis of 2-(4-Bromophenyl)-4-Chloroquinolines **20**, **23a**–**e** and **23i** with Variable Substitution of the Benzenoid Ring (R_1_, R_2_, and R_3_) from β-ketoesters **17a** and **17b** ^a^

a Reactions (a): 60% oil suspension NaH, diethyl carbonate, THF, 54%; (b): catalytic *p-*TsOH·H_2_O, benzene, reflux 3–7 days; (c): Dowtherm A, 250 °C, 2–16 min, 19–55%; (d): POCl_3_, CHCl_3_, reflux 2–5 days, 51–97%.

**Scheme 5:**
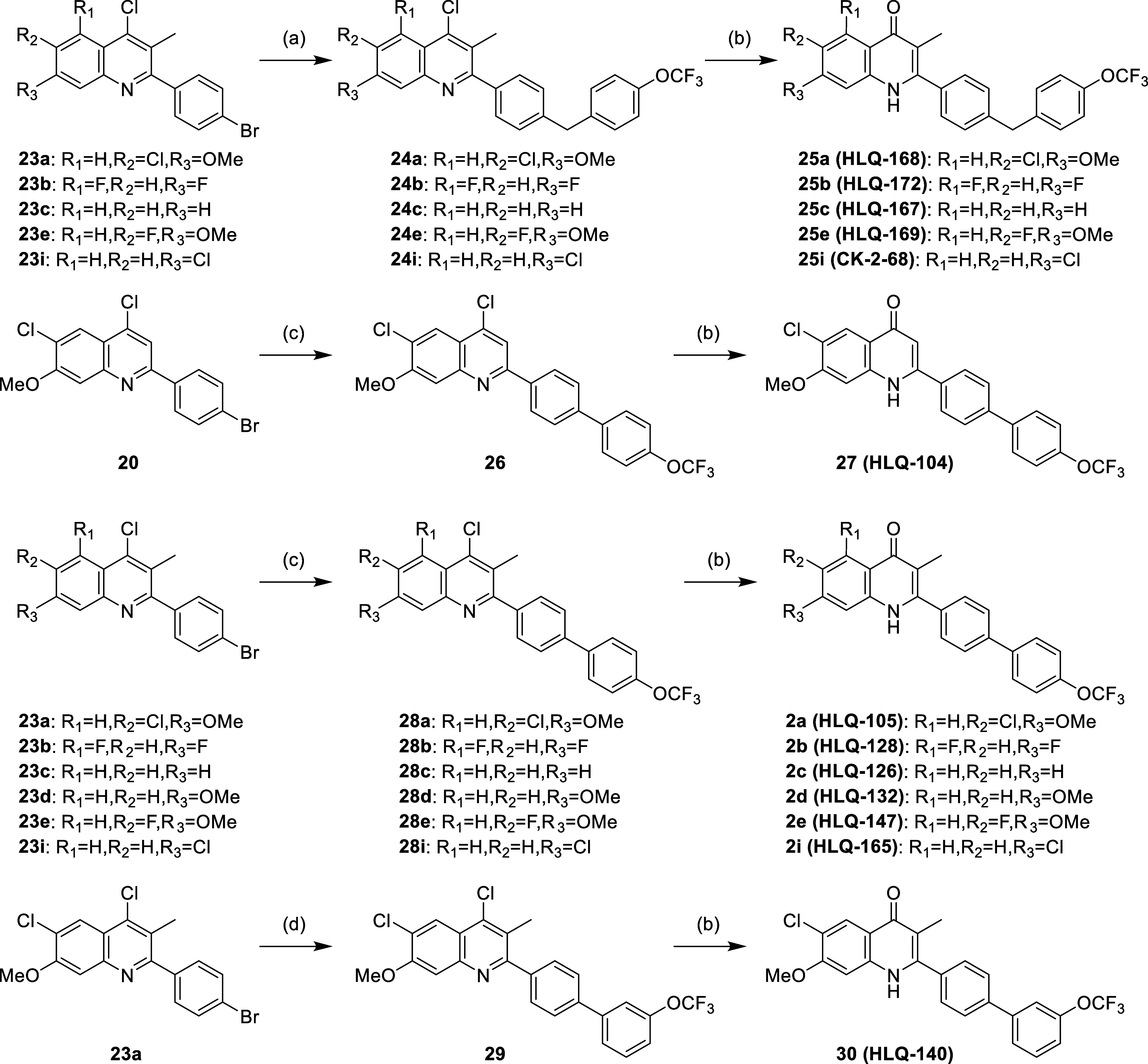
Synthesis of 2-Diphenylmethane and 2-Biaryl Quinolones^a^

a Reaction (a): 4-(trifluoromethoxy)­benzyl boronic acid pinacol ester, Pd­(dppf)­Cl_2_, K_2_CO_3_, toluene/dioxane/water (10:1:1), 90 °C, 24 h, 36–52% yield; (b): KOAc, AcOH, 16–24 h, 110–120 °C, 2–91%; (c): 4-(trifluoromethoxy) phenyl boronic acid, Pd­(dppf)­Cl_2_, K_2_CO_3_, DMF, 80 °C, 18–24 h, 37–72%; (d): 3-(trifluoromethoxy) phenyl boronic acid, Pd­(dppf)­Cl_2_, K_2_CO_3_, DMF, 80 °C, 18–24 h.

The synthesis of the intermediate 2-(4-bromophenyl)-4-chloroquinolines **20**, **23a**–**e** and **23i** is shown in Scheme 4. The key intermediate β-ketoester **17b** has been reported^38^ by reacting **17a** with methyl iodide in the presence of K_2_CO_3_. In our study, we prepared **17b** in 54% yield by reacting 4-bromo-propiophenone **16b** with diethyl carbonate in the presence of NaH. The desired quinolones **19**, **22a**–**22e** and **22i** were then synthesized via Conrad–Limpach reaction of the corresponding Schiff bases **18**, **21a**–**21e** and **21i** in 19–55% yield. For the quinolone **22i** both the 5- and 7-chloro regioisomers were obtained after cyclization, which could not be separated by recrystallization. To facilitate purification, these regioisomers were converted to the corresponding pivalate esters and purified by flash chromatography. The desired regioisomer was then hydrolyzed in the presence of *p-*TsOH·H_2_O to give pure **22i**. Next, these quinolones **19**, **22a**–**22e** and **22i** were treated with POCl_3_ to provide the corresponding 4-chloro quinolines **20**, **23a**–**23e** and **23i** in good yield.

With the 2-(4-bromophenyl)-4-chloroquinoline intermediates **20**, **23a**–**23e** and **23i** in hand, selective Suzuki reaction with 4-(trifluoromethoxy)­benzyl boronic acid pinacol ester, 4-trifluoromethoxyphenylboronic acid and 3-trifluoromethoxyphenylboronic acid in the presence Pd­(dppf)­Cl_2_ and K_2_CO_3_ in DMF afforded the desired 4-chloro-quinolines **24a**–**24c**, **24e**, **24i**, **26**, **28a**–**28e**, **28i** and **29**, which were then converted to their corresponding 2-substituted quinolones **2a**–**2e**, **2i**, **25a**–**25c**, **25e**, **25i**, **27** and **30** using KOAc in AcOH (Scheme 5).

We chose **1a** and **2a** for our in vivo work. For this purpose, we synthesized the corresponding alkoxycarbonate ester prodrugs **31** and **34** using chloromethyl ethyl carbonate, tetra-n-butylammonium iodide (TBAI), and K_2_CO_3_ in DMF according to a previously published method (Scheme 6).^39^ Since *N*-hydroxy quinolones are also active against malaria parasites,^13,18^ we were interested in synthesizing the *N*-hydroxy versions of **1a** and **2a** for further evaluation. For this purpose, we followed our published procedure^40^ by oxidizing **31** and **34** with meta-chloroperbenzoic acid (mCPBA) to the corresponding *N*-oxides **32** and **35**, which were then hydrolyzed with 10% aqueous NaOH in alcohol to afford the desired *N*-hydroxy quinolones **33** and **36** in 82–93% yield.

**Scheme 6:**
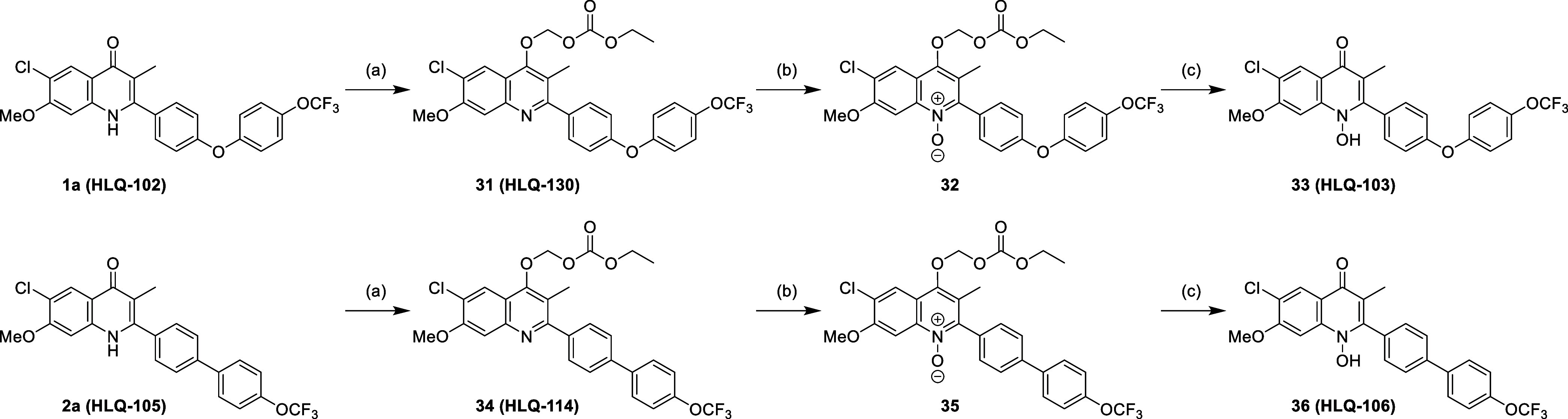
Synthesis of Alkoxy Carbonate Prodrugs **31** and **34** and the Corresponding *N*-Hydroxy Quinolones **33** and **36** ^a^

a Reaction: (a) chloromethyl ethyl carbonate, TBAI, K_2_CO_3_, DMF, 60 °C, 24 h, 57–58%; (b) *m*CPBA, CHCl_3_, 90 °C, 24 h, 49%–63%; (c) MeOH–EtOH/10% NaOH, 2 h-16 h, 82–93%.

### In Vitro Antiplasmodial Activities and Structure–Activity Profiling of HLQs

We assessed the antiplasmodial IC_50_ values vs *P. falciparum* strains D6 (drug sensitive) and Dd2 (multidrug resistant) as well as Tm90–C2B (ATV^R^ clinical isolate) and D1 (ELQ-300^R^). The “C2B” isolate carries a point mutation in the region of the cyt b gene that codes for the Q_o_ site which brings about a Y268S transformation in the associated protein sequence.^41^ For the D1 clone, which was derived from the parental Dd2 strain under ELQ-300 pressure, an I22L transformation occurred through a point mutation in the region of the cyt b gene that codes for the Q_i_ site.^8^ IC_50_ values were determined by the SyBr Green assay as described in 2004.^42,43^

### Design of HLQs for Q_o_ or Q_i_ Targeting of *Pf* cyt *bc*_1_

For these assays we included ATV, ELQ-300, ELQ-596, and ELQ-400 as controls that target either the Q_o_ or Q_i_ sites of *Pf* cyt *bc*_1_ (Tables 1–5). For example, notice that ATV yields subnanomolar IC_50_ values against all strains except the highly resistant Tm90–C2B clinical isolate, whereas ELQ-300 provides low nM IC_50_’s except for the D1 clone that carries a mutation in the Q_i_ site.

**Table 3:**
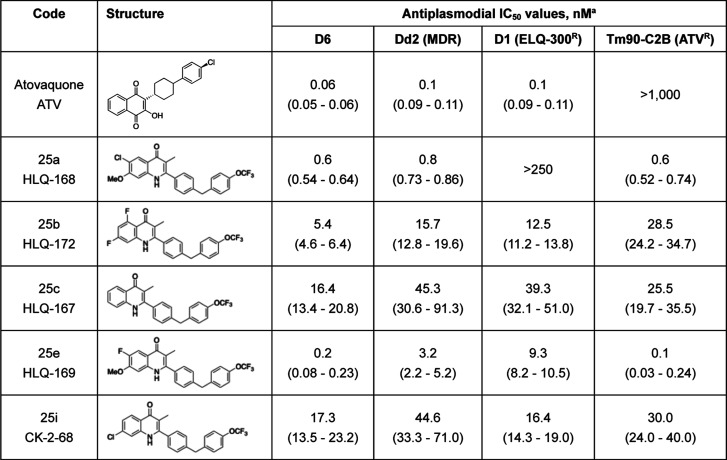
Comparative IC_50_ Values for Select ELQs and HLQ Derivatives Where a Substituted Diphenylmethane Group is at the 2-Position^a^

a IC_50_ values represent the concentration of drug that suppresses parasite growth by 50% relative to controls without addition of drug. For mean IC_50_ values, minimally quadruplicate data were collected for each concentration in the dose–response curves, and error represents the 95% confidence interval of the fit. For each test agent, we show the results from a single representative experiment.

**Table 4:**
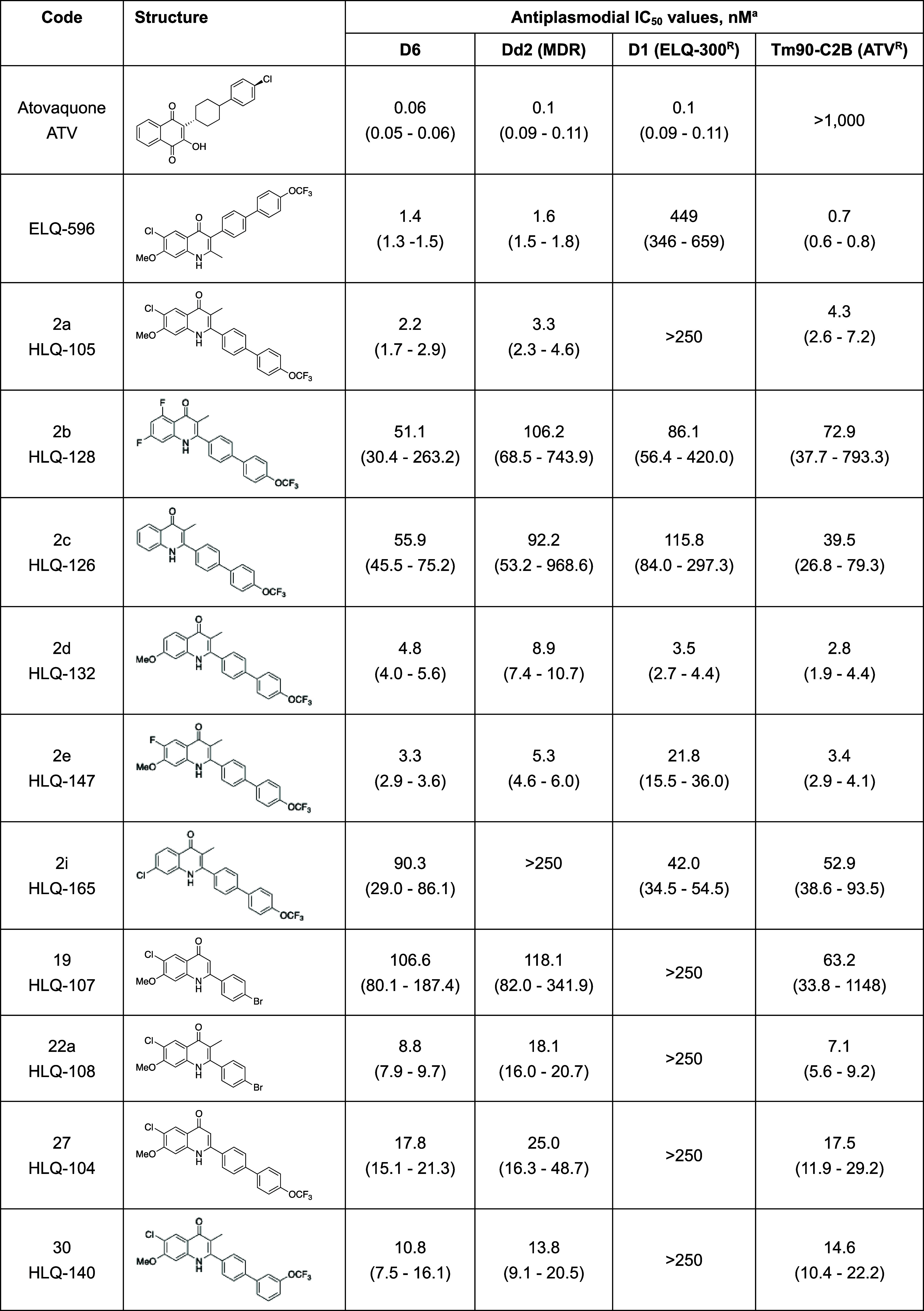
Comparative IC_50_ Values for Select ELQs and HLQ Derivatives Containing a Substituted Aryl or Biaryl Group at the 2- or 3-Position^a^

a IC_50_ values represent the concentration of drug that suppresses parasite growth by 50% relative to controls without addition of drug. For mean IC_50_ values, minimally quadruplicate data were collected for each concentration in the dose–response curves, and error represents the 95% confidence interval of the fit. For each test agent, we show the results from a single representative experiment.

**Table 5:**
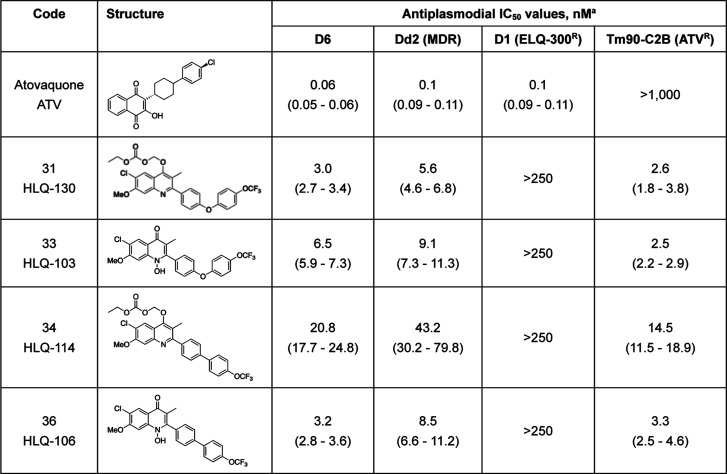
Comparative IC_50_ Values for Select HLQ Prodrugs and *N*-Hydroxy Derivatives Where the Bulky Substituent is at the 2-Position^a^

a IC_50_ values represent the concentration of drug that suppresses parasite growth by 50% relative to controls without addition of drug. For mean IC_50_ values, minimally quadruplicate data were collected for each concentration in the dose–response curves, and error represents the 95% confidence interval of the fit. For each test agent, we show the results from a single representative experiment.

### HDQ and HQ

HDQ was included since it served as the structural lead for Stephen Ward’s group to carry out a preliminary investigation of 2-substituted quinolones for antimalarial activity.^13^ It is noteworthy that HDQ displays relatively modest IC_50_ values against all tested strains, in the ∼30 to ∼80 nM range (Table 1). Prior studies have shown this molecule to target both the Q_o_ and Q_i_ sites of mammalian cyt *bc*_*1*_.^13,44^ Derivative HQ, lacking the *N*-hydroxy component, shows a similar pattern of activity, however, compound potency is improved across all strains.

### SAR: Alkyl Substitutions at the 3-Position (ELQ) and 2-Position (HLQ)

By comparing the IC_50_ values of ELQ-121 and HLQ-118 (**9b**) we see low nM IC_50_ values for both compounds together with strong cross-resistance vs the C2B strain, suggesting Q_o_ targeting for both despite the regional isomery of the extended alkyl group at the 3- and 2-positions, respectively (Table 1). A similar pattern is seen for the isomer pair of ELQ-432 and the 2-position alkyl substituted variant, HLQ-119 (**9a**), with very low nM IC_50_’s across the panel of tested strains except for the D1 clone, from which we infer Q_i_ targeting.

### SAR: Aryl Substitutions (Diphenyl Ether and Biphenyl) at the 3-Position (ELQ) and 2-Position (HLQ)

HLQ derivatives containing the 6-chloro/7-methoxy substitution pattern on the benzenoid ring such as HLQ-119 (**9a**), HLQ-120 (**6**), HLQ-104 (**27**) and HLQ-105 (**2a**) show strong cross resistance vs the D1 strain, indicating that these are Q_i_ targeting (Tables 1 and 2). It is interesting to compare HLQ-104 (**27**), where the 3-CH_3_ group is absent, to the identical analog but containing the 3-methyl group, i.e., HLQ-105 (**2a**). Note that antiplasmodial activity is diminished by 10- to 20-fold in the absence of this methyl group. Here it seems likely that the presence of the 3-CH_3_ group serves to force the innermost ring to rotate out of coplanarity with respect to the quinolone core which decreases pi–pi stacking and crystallinity. The 3-methyl group may also enhance contacts between the side chain and amino acid residues lining the Q_i_ pocket. As shown in Table 1, the enhanced activity of 3-methyl-HLQs vs their des-methyl counterparts even extends to derivatives that merely have an extended alkyl substituent at position 2 (see HLQ-119 (**9a**) vs HLQ-120 (**6**)) so the role of the 3-methyl group appears to go beyond sterics.

Most impressive are the low to sub nM IC_50_ values observed for both HLQ-102 (**1a**) and HLQ-105 (**2a**), the 2-position regioisomers of ELQ-300 and ELQ-596, respectively (Tables 2 and 4). We also observe very similar potencies across all strains tested except for a high level of cross resistance against the ELQ-300^R^ clone D1, strongly suggesting that both HLQ-102 (**1a**) and HLQ-105 (**2a**) inhibit the *P. falciparum* cyt *bc*_*1*_ complex by targeting the Q_i_ site. *N*-Hydroxylation of HLQ-102 (**1a**) to form HLQ-103 (**31**) leads to a significant decrease in potency while the impact of this same chemical transformation is relatively modest when applied to the biaryl HLQ-105 (**2a**) on conversion to HLQ-106 (**34**) (Table 5). Taking these findings together and to our surprise, we find that the 6-halo/7-methoxy substitution pattern on the quinolone core appears to direct the agent toward the Q_i_ site, regardless of whether the bulky diphenyl ether side chain or the rigid biaryl group is at the 2- or the 3-position of the quinolone. Further support for this comes from comparing HLQ-102 (**1a**) and HLQ-175 (**1k**) where the 6-chloro/7-methoxy substituent pattern on the benzenoid ring is reversed, HLQ-175 (**1k**). This structural change is accompanied by a significant loss of potency (>100-fold) (Table 2). We believe that these results show that the 6-chloro/7-methoxy configuration together with the 4-position carbonyl are key to setting the proper docking pose of the molecule within the Q_i_ site and thus for selective targeting of the parasite cytochrome bc_1_ complex.

It is interesting that this pattern does not seem to follow for HLQs with the 5,7-difluoro substitution pattern (Tables 1, 2, 3, and 4). While ELQ-400 exhibits low nanomolar intrinsic potency vs strains D6, Dd2, and D1, there is cross resistance vs the ATV^R^ Tm90–C2B strain—an indication of Q_o_ targeting. For HLQ-127 (**1b**), the 2-position regioisomer of ELQ-400, potency is diminished by over 10-fold and cross-resistance vs either Tm90–C2B or D1 is insignificant. This pattern is repeated for the substituted 2-biaryl HLQ-128 (**2b**). From these results, we speculate that the Q_i_ site has two distinct troughs to accommodate the biaryl and diphenyl ether substituents at the 2- or 3-positions while at the Q_o_ site there may only be a single trough that accommodates the bulky side groups at the 3-position. Future structural biology studies including cryo-EM may help to show whether this speculation has merit.

### SAR: 2-Biaryl vs 2-Diphenyl Ether vs 2-Diphenylmethane Side Chains

Here it is important to highlight CK-2–68 (**23i**) from the Ward and O’Neill lab where a methylene group links the two aromatic rings of the 2-position side chain. In our hands, we found IC_50_ values for this compound in the respectable range of 17.3–44.6 nM vs all tested strains, which is consistent with their earlier report (Table 3). In vitro potencies for CK-2–68 (**23i**) are greater than what is observed for the corresponding 2-biaryl analog, HLQ-165 (**2i**), as well as the diphenyl ether variant, HLQ-164 (**1i**). IC_50_ values are most impressive for HLQ-102 (**1a**) analog, HLQ-168 (**23i**), which contains a substituted diphenylmethane side chain, with subnanomolar values across the panel of test strains except for the D1 clone where extreme cross resistance is observed. Overall, it appears that replacement of the oxygen atom in the diphenyl ether side arm at the 2-position enhances in vitro potency for both Q_i_ and Q_o_ targeting HLQ scaffolds. Despite this finding we chose HLQ-102 (**1a**) and HLQ-105 (**2a**) as lead molecules for the project based on their impressively low nM IC_50_ values, Q_i_ targeting, and ease, low-cost and scalability of the synthetic methods used to prepare them. We also observed that some highly active HLQs, e.g., HLQ-125 **(1c)**, HLQ-132 **(2d)**, and HLQ-135 **(1d)**, lack in vitro cross resistance to either ATV^R^ Tm90–C2B or ELQ-300^R^ D1 (Tables 2 and 4). This phenomenon was also observed for a subseries of ELQs described by Stickles et al. in 2015.^8^ Investigations are underway to identify the associated mechanism(s) of action including Q_o_-targeting, Q_i_-targeting, Q_o_Q_i_-dual site targeting or inhibition of some other element of the parasite electron transport chain.

### In Vitro Liver Stage Assessment of HLQs Against *Plasmodium berghei*

Next, we assessed selected HLQs for in vitro liver stage activity to compare with standards ELQ-300 and atovaquone. Initially we used a high throughput in vitro liver stage system to measure inhibitory potency on the development of hepatic schizonts using a transgenic *P. berghei* strain carrying markers for green fluorescent protein (GFP) and luciferase (PbGFP-Luc_con_). As described in Methods, Hep-G2 cells were infected with PbGFP-Luc_con_ sporozoites followed by addition of test agents and standards. Following incubation for 48 h, parasite burden was quantified by bioluminescence imaging after the addition of D-luciferin substrate. As shown in Figure 3 and Table 6, the standard compounds, atovaquone and ELQ-300, exhibited low nanomolar IC_50_ values against *P. berghei* replication in Hep-G2 cells in vitro with values of 6.7 nM and 2.4 nM, respectively. The mean IC_50_ value for HLQ-102 was 3.6 nM, which is quite similar to the potency recorded for atovaquone and slightly less in comparison with ELQ-300 despite the structural change from a 3-position diarylether to the 2-position isomer. HLQ-105 is somewhat less potent against liver stage *P. berghei* parasites which is consistent with earlier observations for relative inhibitory potency against blood stage *P. falciparum* parasites. Surprisingly, the unsubstituted HLQ-125 does not have an inhibitory effect on parasite growth in this system at concentrations as high as 100 nM whereas the 6-fluoro-7-methoxy analog of HLQ-102, i.e., HLQ-133, has an IC_50_ value of 10 nM.

**Figure 3:**
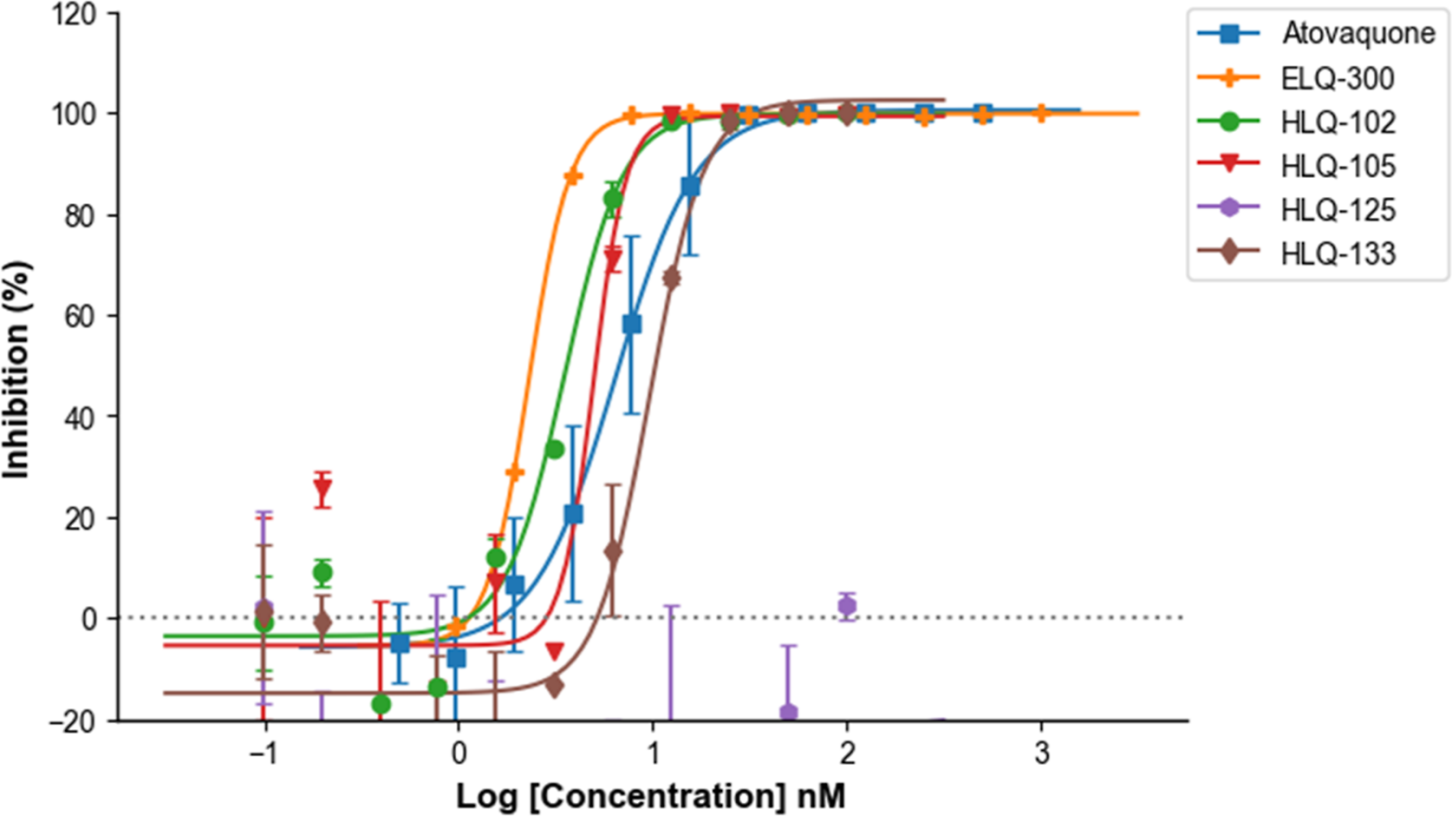
Dose–response in vitro liver stage activity of HLQs and reference controls against *P. berghei* in HepG2 cells. HepG2 monolayers were infected with *P. berghei* sporozoites, compounds were added postinvasion, and parasite burden was quantified at 48 h by measuring the luciferase signal. Data are normalized to uninfected and vehicle controls; curves are representative of 3 independent experiments. Error bars represent SEM. Atovaquone and ELQ-300 are shown as antimalarial comparators. IC_50_ values are in Table 6.

**Table 6:**
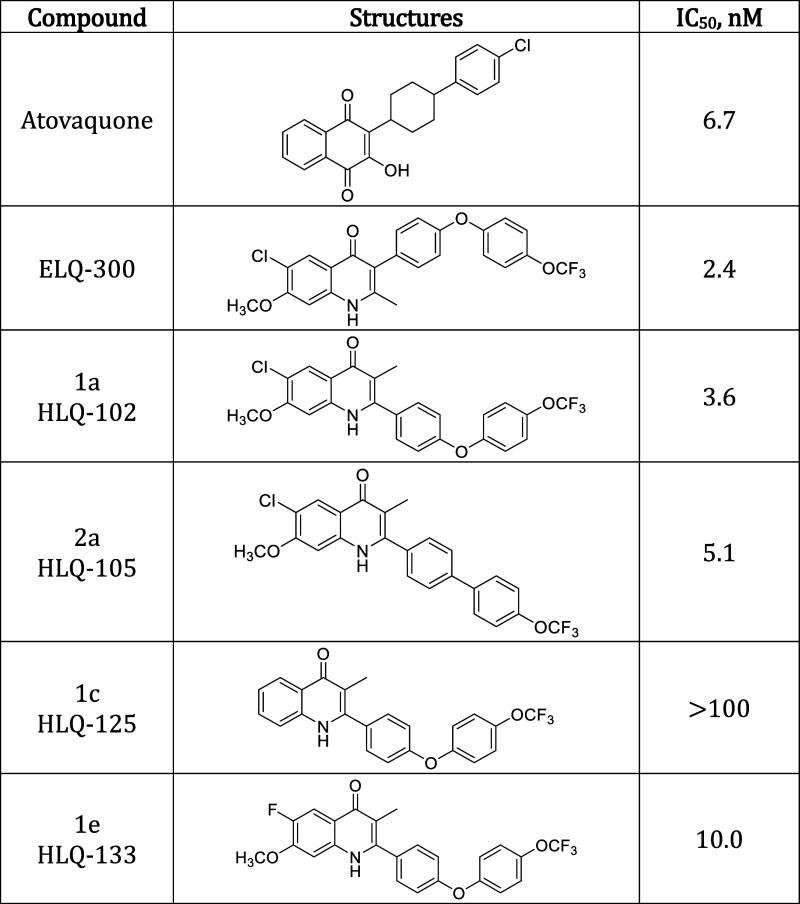
In Vitro Liver Stage Activity of Selected HLQs and Reference Compounds Against *P. berghei* Infection in Hep-G2 Cells

### In Vitro Liver Stage Assessment of HLQs Against *Plasmodium cynomolgi*, Replicating and Dormant Stages

We then evaluated selected HLQs for activity against replicating (schizont) vs dormant (hypnozoite) stages of *P. cynomolgi* development in nonhuman primate hepatocytes. This “anti-relapse” assay uses a 384-well format for high throughput screening of compounds to assess the impact of candidate antimalarials on growth and development of both schizont and hypnozoite forms of *P. cynomolgi*. Briefly, primary NHP hepatocytes were seeded to confluency and then infected with sporozoites obtained upon dissection of infected *Anopheles dirus* mosquitoes. To assess for prophylactic activity, test compounds were added to each well 1 h following sporozoite invasion on Day 0 and subsequent dosing on Days 1 and 2, with readout on Day 8. To assess for radical cure potential, test compound exposure began after establishment of parasite infection with compound addition beginning on Days 4, through Days 5, 6, and 7 post sporozoite infection. In radical cure mode the readout was also on Day 8. Selected HLQs were evaluated for prophylaxis and radical cure potential in this in vitro system alongside reference drugs maduramicin, KDU691, tafenoquine, atovaquone, and ELQ-300 (Table 7). In prophylaxis mode KDU691 and atovaquone potently inhibit formation of *P. cynomolgi* schizonts (IC_50_ = 100 nM and 6.0 nM, respectively) and to a lesser extent formation of hypnozoites (IC_50_ = 130 nM and 380 nM, respectively). The inhibitory activity of atovaquone against schizonts and hypnozoites in radical cure mode is greatly diminished while KDU691 acts selectively against schizonts (IC_50_ = 390 nM). The ionophore antibiotic maduramicin exhibits submicromolar potency against schizonts and hypnozoites in both prophylaxis and radical cure mode. The clinical drug tafenoquine exhibits low to submicromolar potency against hypnozoite and schizont formation in prophylaxis mode while activity is diminished in the selected simian donor (QHO) for radical cure mode, likely because it is known to require extensive metabolism by the host P450 system for full efficacy and monkey donors have variable metabolomes in this regard. And for both HLQ-102 and ELQ-300 their effectiveness in prophylaxis mode is quite similar to that seen for atovaquone, with submicromolar IC_50_’s vs both schizonts and hypnozoite forms but without observable inhibitory effects in radical cure mode. These results suggest that as single agents the antirespiratory compounds are most effective if drug exposure occurs before or soon after sporozoite invasion. For the other HLQ derivatives shown in the table there is a similar pattern of inhibition of liver stage parasite development in prophylaxis mode and lack of activity once the schizonts and hypnozoites have become established.

**Table 7:**
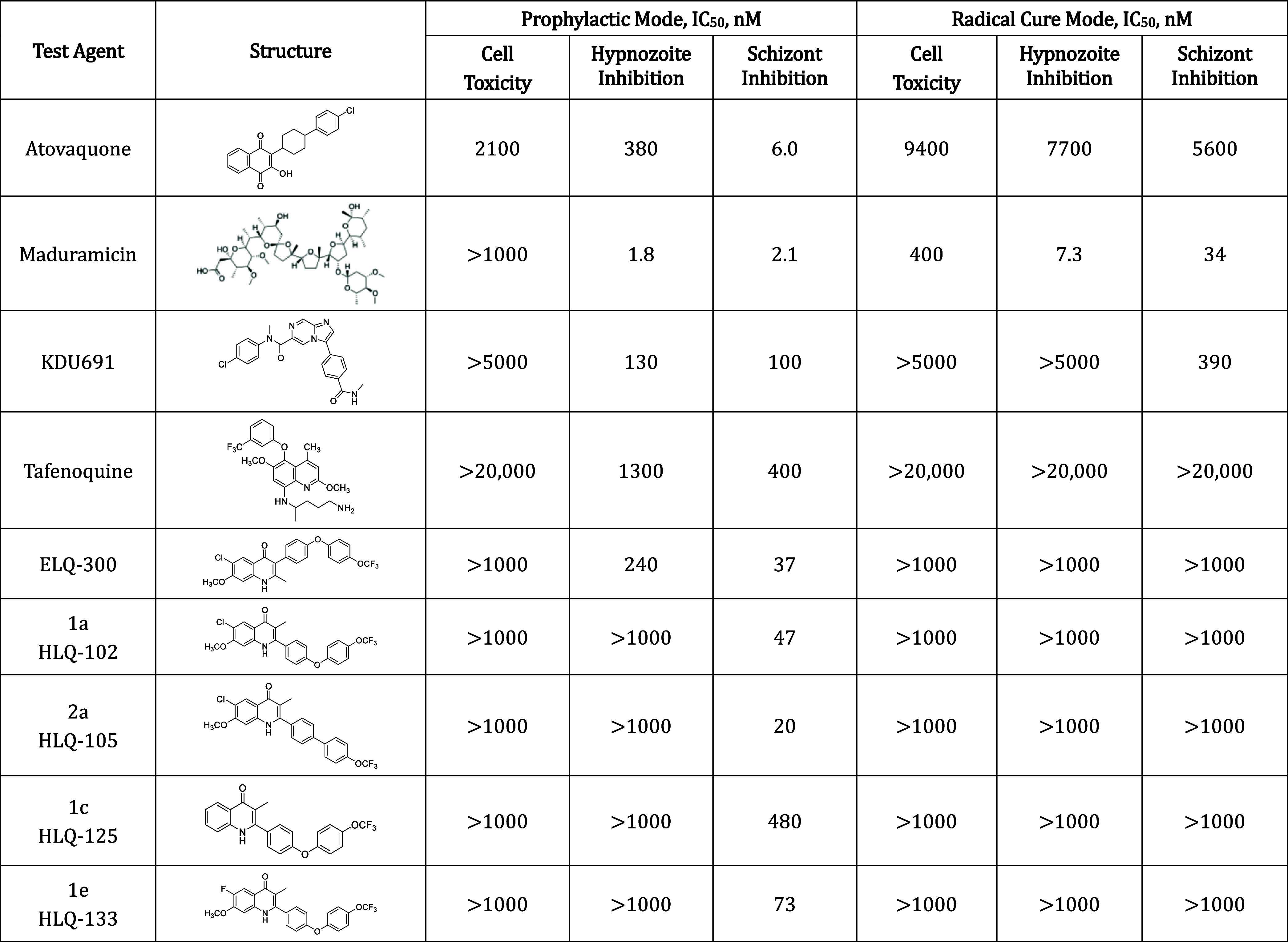
In Vitro Liver Stage Activity of Selected HLQs and ELQs against *P. cynomolgi* Infections (Schizont and Quiescent Forms) in Non-Human Primate Hepatocytes^a^

a IC_50_ curves can be found in Supporting Information.

### Comparative Crystal Structure Information for HLQ-102 (1a), ELQ-300 and HLQ-105 (2a)

An overlay of the X-ray crystal structures of HLQ-102 (**1a**) and HLQ-105 (**2a**) (Figure 4) shows that the quinolone core remains unchanged. In contrast, the angular diaryl ether substituent of HLQ-102 (**1a**) occupies a spatial region distinct from that of the more rigid biphenyl projection in HLQ-105 (**2a**). Significantly, an overlay of HLQ-102 (**1a**) with ELQ-300 (Figure 5) further reveals differences in orientation and spatial occupancy resulting from the diaryl ether substitution at the 2-position versus the 3-position of the 6-chloro-7-methoxy quinolone scaffold. The distance between the planes of the inner and outer phenyl rings of the diaryl ether groups of HLQ-102 (**1a**) and ELQ-300 are 4.382 Å and 11.791 Å, respectively. That both HLQ-102 (**1a**) and ELQ-300 target the same site suggests the possibility that the *P. falciparum* cytochrome bc_1_ Q_i_ site contains two discrete troughs or pockets to accommodate their bulky side chains adopting distinctly different orientations. We speculate that the existence of two such troughs might have evolved over time to accommodate the binding of both orientations of coenzyme Q for efficiency as shown in Figure 6. For reference we also include the 3D structures of HLQ-102 (**1a**), HLQ-105 (**2a**) and ELQ-300 (previously unpublished) obtained by X-ray diffraction in the Supporting Information.

**Figure 4:**
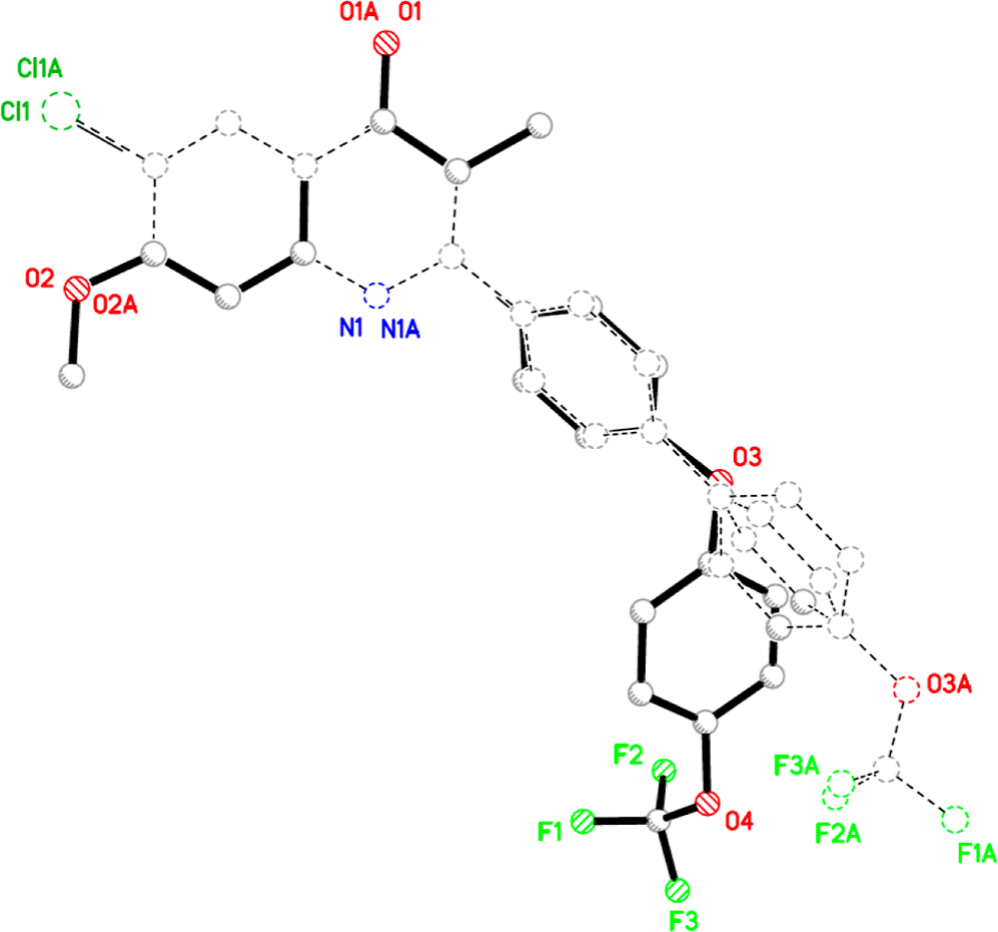
An overlay of the ORTEP (Oak Ridge Thermal Ellipsoid Plot) diagram of HLQ-102 (bold line) and HLQ-105 (dashed line). Ellipsoids are drawn at the 30% probability level.

**Figure 5:**
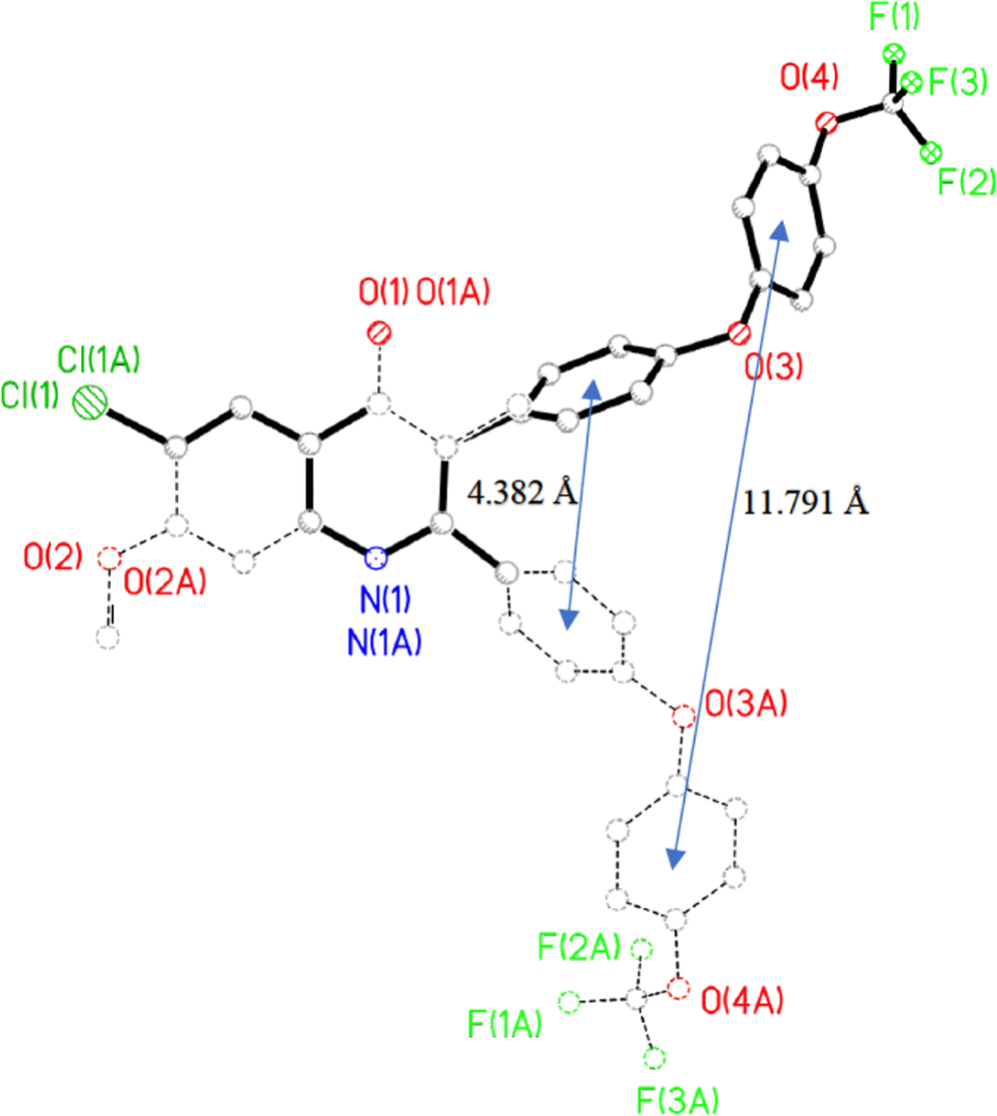
An overlay of the ORTEP diagram of ELQ-300 (bold line) and HLQ-102­(dashed line). Ellipsoids are drawn at the 30% probability level.

**Figure 6:**
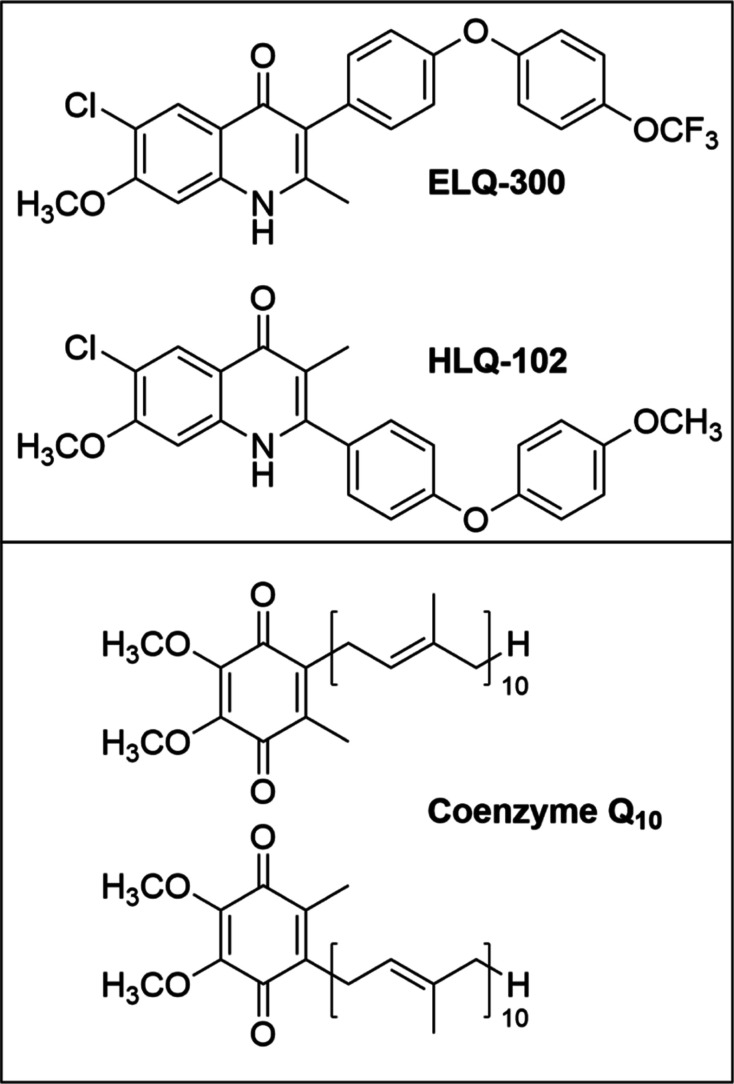
Chemical structures of **ELQ-300** and its 2-position isomer, **HLQ-102** (**1a**), along with the chemical structure of coenzyme Q in two different orientations.

### Structural Biology and Modeling of Isomers HLQ-102 and ELQ-300 into *P. falciparum* cyt bc_1_

As described in the Materials and Methods section, we homology-modeled the three-dimensional structure of *P. falciparum* cytochrome *b* using the *Toxoplasma gondii* bc_1_ crystal structure as a template for the primary sequence of the *Plasmodium* cytochrome *b*. Our model takes advantage of the knowledge of ELQ-300 bound to the Q_i_ site of the *T. gondii* cryoEM crystal structure.^45^ The docking scores of ELQ-300 and HLQ-102 are summarized in Table S1. The GOLD docking scores for ELQ-300 were largely unaffected by the presence of lipid molecules, indicating minimal lipid dependence for pose selection or binding fitness. In contrast, the docking score for HLQ-102 showed a marked and statistically significant increase when lipids were included in the template-derived model, supporting a key role for lipid molecules in stabilizing the HLQ inhibitor series. This lipid-mediated effect was particularly pronounced for inhibitors containing the biphenyl ether tail, suggesting enhanced lipid sensitivity of this pharmacophoric feature within the HLQ scaffold.

The 3D binding poses and 2D protein–ligand interaction maps for the paired inhibitors ELQ-300 vs HLQ-102 are provided in Figure 7. For this inhibitor pairing, the quinolone core adopted a shared conserved binding orientation within the Q_i_ cavity. Specifically, the ring –NH and carbonyl (>C=O) groups of the quinolone moiety in ELQ-300 and HLQ-102 formed hydrogen bonds with Asp218 and His192, respectively. In the ELQ-300 complex, the diphenylether side chain was predominantly stabilized through hydrophobic packing with Phe185, Phe188, Phe189, Leu13, and His12, with no contributions from neighboring lipid molecules that reinforce inhibitor pose stability. Conversely, in the HLQ-102 complex, while the quinolone ring occupied the same cavity as for ELQ-300, the diphenylether group was primarily stabilized by Val9, Leu13, as well as lipid contacts, indicating a stronger lipid contribution for HLQ-102 relative to ELQ-300. Taken together, these results suggest that lipid molecules contribute significantly to the stabilization of HLQ-102 binding at the Q_i_ site, and our homology-guided docking model predicts that separate troughs accommodate the diphenylether side chains of ELQ-300 and HLQ-102 within the *Pf*cytbc_1_ complex. It is noteworthy that X-ray crystallography shows that the distance between the innermost aromatic rings at the 2- and 3-positions of HLQ-102 and ELQ-300, respectively, is 4.382 A (Figure 5), and the docking model shown here in Figure 7 and in Supporting Information (Figures S3–S6) finds that the two troughs are separated by 4 Å as well. This docking model also places the I22 residue of cytochrome *b* in close proximity to the 6-position chlorine atom of both compounds which is consistent with prior work by Stickles et al.,^8^ showing that a mutation at this site leads to ELQ-300 resistance in *P. falciparum*. Definitive proof of this docking mode for HLQ-102 awaits further investigation using cryo-EM technology to establish the precise HLQ-102 docking poise.

**Figure 7:**
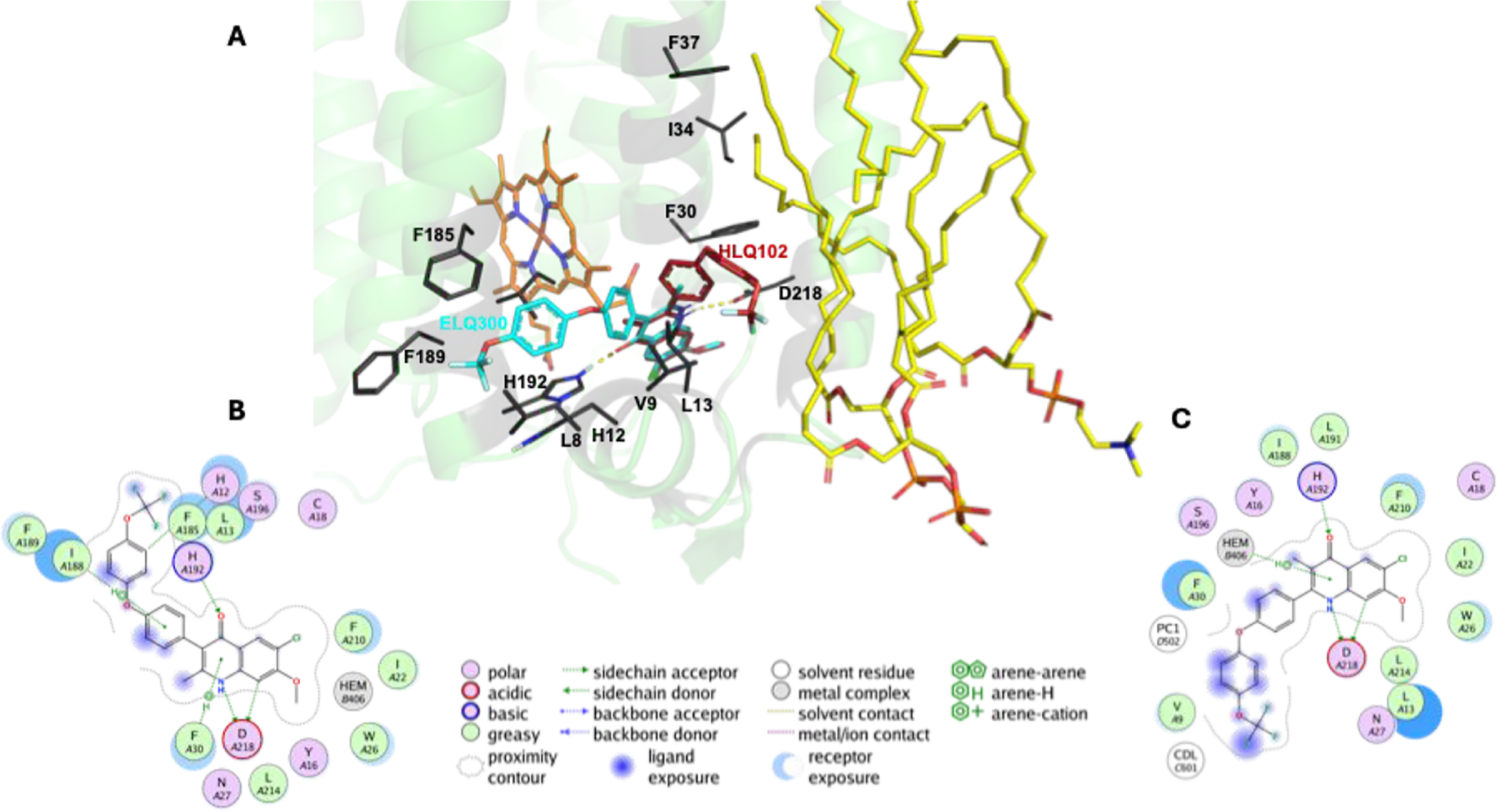
Structural analysis of ligand binding modes in *P. falciparum* cytochrome bc_1_ complex (PfCytbc_1_). Binding poses were predicted by in silico docking. The protein is represented as ribbons and colored green. Key residue side chains, ligands and lipid molecules are depicted as sticks (H, white; O, red; N, blue). Hydrogen bonds and other polar interactions are indicated by yellow dashed lines. (A) Three-dimensional binding pose of ELQ-300 (cyan) and HLQ-102 (brick red) in the PfCytbc_1_ Q_i_ site. Part of the HLQ-102 is stabilized by the lipid molecules. (B,C) Two-dimensional ligand interaction diagrams for (B) ELQ-300 and (C) HLQ-102. Residues are colored by chemical type, and interaction types are defined in the legend. PC1 and CDL indicates phosphatidylcholine and cardiolipin lipids, respectively. The *T. gondii* crystal structure of cytochrome bc_1_ complex was used to create a homology model of the *P. falciparum* enzyme for these ligand binding studies (PDB ID: 9I4X).

### Cytotoxicity of HLQ-102 (1a) and HLQ-105 (**2a**) vs Mammalian Cells in Vitro

To test toxicity against human cells, confluent monolayers of human foreskin fibroblasts (HFFs) were exposed to serial dilutions of HLQ-102 (**1a**), HLQ-105 (**2a**), and rotenone from 19 nM to 10 μM. Compounds were tested in both glucose medium and glucose-free medium supplemented with galactose. Glucose medium consisted of DMEM with 25 mM glucose, 1 mM sodium pyruvate, and 1x GlutaMAX. Galactose medium consisted of DMEM supplemented with 25 mM galactose, 1 mM sodium pyruvate, and 1xGlutaMAX. Importantly, use of galactose rather than glucose forces the cells to produce ATP through oxidative phosphorylation (effectively reversing the “Crabtree Effect”), which then makes them sensitive to mitochondrial toxicants.^46^ Briefly, HFF cells were allowed to proliferate in each well of a 384-well plate until a confluent monolayer had been reached across the entire plate. At this time the culture medium was removed and replaced with complete medium consisting of DMEM-Glucose or DMEM-Galactose as described above and with or without test agent or positive control. After 72 h of exposure, ATP levels of the HFF cells were quantified using a CellTiter-Glo kit (Promega) by luminescence. 50% cytotoxic concentrations (CC_50_) were calculated by nonlinear regression analysis using Prism software.^47^ As shown in Table 8, the CC_50_ was >10 μM for HLQ-102 (**1a**) and **HLQ-105** (**2a**) in both glucose and galactose media. The CC_50_ of rotenone, a complex 1 inhibitor used as a positive control, was >10 μM in glucose media and <19 nM in galactose media. ATP levels were not significantly different between control HFFs (drug-free condition) cultured for 72 h in glucose media or galactose media. The data combine to show that HLQ-102 (**1a**) and HLQ-105 (**2a**) are not cytotoxic toward a reference human cell line, HFF, under conditions requiring an intact and functioning mitochondrial electron transport chain at concentrations as high as 10 μM.

**Table 8:** Cytotoxicity of **HLQ-102** (**1a**) and **HLQ-105** (**2a**) Against Human Foreskin Fibroblast Cells (HFF) in Both Glucose- and Galactose-Containing Medium to Address the Impact of a “Crabtree Effect”^b^

compound	cytotoxicity, IC_50_ value, μM^a^	
	+glucose	+galactose
rotenone	>10 μM	≪19 nM
HLQ-102 (1a)	>10 μM	>10 μM
HLQ-105 (2a)	>10 μM	>10 μM

a Compounds were tested in DMEM medium supplemented with 10% Fetal Clone 1 serum (Hyclone), 50Units/mL penicillin (Gibco), 50 μL/mL streptomycin (Gibco), and 1x GlutaMAX (Gibco). Test plates were incubated in a humidified incubator at 37 °C and 5% carbon dioxide for 72 h before final assessment using the TiterGlo test reagent. Assays were performed in quadruplicate.

b Rotenone Served as the positive control inhibitor.

### Lack of Inhibition of the Bovine Cytochrome bc_1_ Complex by HLQ-102 (**1a**) and HLQ-105 **(2a)**

We used the bovine system for assessment of inhibition of mammalian cytochrome bc_1_ activity by selected test HLQs. Mitochondria were purified from bovine heart tissue by following a published procedure^48^ as detailed in the Methods section. Inhibition assays were performed as described previously,^6,49^ substituting azoxystrobin for antimycin A as a positive control. Test agents were dissolved in DMSO to create 1 mM stock solutions. Final protein concentration in each assay was adjusted to 1.5 μg in a total volume of 200 μL. Rates of cytochrome bc_1_ reduction for each compound were measured by taking the slope of (Abs_550_-Abs_542_/time across a 7-point dilution series (2-fold from 20 μM) with an eighth control condition containing no test compound. Azoxystrobin (10 μM) was used to demonstrate full inhibition of the cytochrome bc_1_ complex with a slope of ∼0. In contrast, **HLQ-102** (**1a**) and **HLQ-105** (**2a**) displayed a slope of between 0.0017 and 0.0020, matching that of the uninhibited control condition and indicating no inhibition of bovine cytochrome bc_1_ activity at **HLQ-102** (**1a**) concentrations up to 10 μM.

### Metabolic Stability of HLQ-102 (**1a**) and HLQ-105 (**2a**) in the Presence of Murine and Human Microsomes

We then evaluated HLQ-102 (**1a**) and HLQ-105 (**2a**) for metabolic stability in the presence of pooled murine hepatic derived microsomes. Because of their close structural similarity to ELQ-300 and ELQ-596, we expected both HLQs to be stable under the assay conditions. The drugs were incubated in the presence of pooled murine microsomes (0.5 mg/mL) at 37 °C in the presence of NADPH to test for P450 drug dependent metabolism. Samples were taken over the interval of 45 min and analyzed by LC–MS/MS for the presence of parent compound. Ketanserin served as the internal standard for the metabolic rate of a drug with known intermediate stability (*T*_1/2_ = 9.07 min). As shown in Table 9, tests demonstrate moderately high stability for HLQ-105 (**2a**) with a measured *T*_1/2_ of 84.62 min while extreme microsomal stability for HLQ-102 (**1a**) was observed, with negligible breakdown over the course of 45 min of incubation, yielding a *T*_1/2_ of >4000 min. The superior metabolic stability of HLQ-102 (**1a**) in murine microsomes prompted us to evaluate this drug for stability in microsomes from other preclinical species, including humans. These experiments showed that **HLQ-102** (**1a**) exhibits a high degree of stability toward microsomal attack in the presence of rat and human microsomes as well.

**Table 9:** Metabolic Stability of **HLQ-102** and **HLQ-105** In Vitro in the Presence of Pooled Murine Hepatic Microsomes

test article	species	percent remaining (%)					*T*_1/2_ (min)	Cl_int_ (mL/min/kg)
		0 min	5 min	15 min	30 min	45 min		
Ketanserin (control)	mouse	100.00	65.43	28.37	8.94	3.26	9.07	601.36
HLQ-105 (2a)	mouse	100.00	85.20	102.24	98.48	60.18	84.62	64.49
HLQ-102 (1a)	mouse	100.00	105.41	103.15	104.05	104.95	>4000	0.00
HLQ-102 (1a)	rat	100.00	97.63	106.04	105.23	101.08	>4000	0.00
HLQ-102 (1a)	human	100.00	101.15	112.12	111.47	102.65	>4000	0.00

### Metabolic Stability of HLQ-102 (**1a**) in the Presence of Viable Hepatocytes

Given that the highest level of microsomal stability was observed for HLQ-102 (**1a**), we next evaluated it for metabolic stability in the presence of viable murine and human hepatocytes. HLQ-102 (**1a**) appears to be completely stable in the presence of human hepatocytes for the full duration of the 120 min incubation (Table 10). The compound was also quite stable in the presence of murine hepatocytes with a *T*_1/2_ of 559 min.

**Table 10:** Metabolic Stability of **HLQ-102** (**1a**) in the Presence of Viable Human and Murine Hepatocytes

test article	species	percent remaining (%)					*T*_1/2_ (min)	clint (mL/min/kg)
		0 min	15 min	30 min	60 min	120 min		
testosterone	human	100.00	18.61	9.72	1.01	BQL	6.18	285.21
	mouse	100.00	3.98	BQL	BQL	BQL	3.22	2539.57
7-Hydroxy-coumarin	human	100.00	36.03	25.50	7.65	5.61	10.18	173.17
	mouse	100.00	13.01	2.40	1.78	1.24	5.10	1605.80
HLQ-102 (1a)	human	100.00	98.08	103.08	99.62	100.38	∞	0.00
	mouse	100.00	95.66	93.14	93.91	84.52	559.12	14.64

### HLQ-102 (**1a**) Metabolite Identification in the Presence of Murine Hepatocytes

Curious to understand the metabolic fate of HLQ-102 (**1a**) in the presence of mouse hepatocytes, we then pursued metabolite identification. To conduct this assessment, HLQ-102 (**1a**) (10 μM) was incubated with mouse hepatocytes at 37 °C in a humidified incubator with 5% CO_2_ in hepatocyte maintenance medium. Samples were taken at 0 and 240 min and were quenched by addition of acetonitrile and analyzed by UPLC-MS. The LC-UV-MS extracted ion chromatograms (EIC) of the *T*_240_ and *T*_0_ time points were compared to identify the major putative metabolites (Table 11). At 240 min a single metabolite exhibited a mass shift of −14 atomic mass units (amu) which we infer to be the 7-*O*-demethylated product (Figure 8). Another metabolite was detected with a mass shift of +65.94 amu which we infer to result from sequential demethylation and sulfation occurring at position 7. The primary metabolite detected following 240 min of incubation (1.48% of total abundance) exhibited a mass shift of +162.01 amu which is inferred to result from demethylation of the methoxy group at position 7 followed by glucuronidation.

**Table 11:** Major Metabolites of HLQ-102 Incubated with Mouse Hepatocytes

peak ID	found m/*z*	mass shift	biotransformation	R.T. (min)	mouse hepatocytes(240 min)	
					UV relative Abundance^a^	MS peak area
parent (*T*_0_)	476.0844	N/A	N/A	5.98	100.00%	8.46 × 10°^6^
parent (*T*_240_)	476.0844	N/A	N/A	5.98	95.69%	8.42 × 10°^6^
M461	462.0734	–14.0121	demethylation	5.06	+	4.32 × 10°^3^
M541	542.0290	65.9435	demethylation + sulfation	5.58	+	6.83 × 10°^3^
M637	638.1028	162.0173	demethylation + glucuronidation	4.07	1.48%	3.34 × 10°^4^

a All percentages were calculated based on the detected UV (λ = 310–320 nm) absorption relative to that of parent in T0 sample (normalized as 100%), +: Only detected in MS.

**Figure 8:**
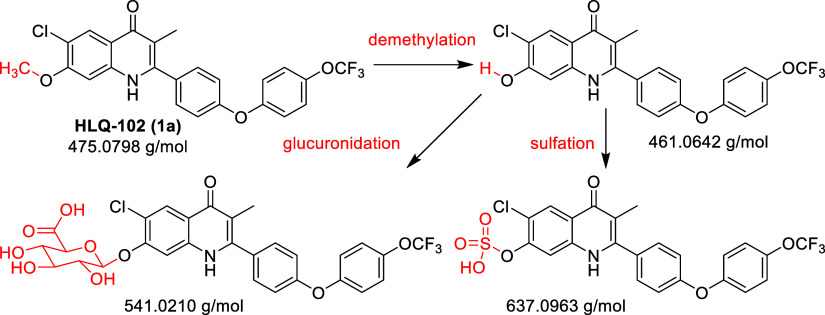
Metabolism of **HLQ-102** (**1a**) in the presence of murine hepatocytes.

### Reduced Crystallinity of the HLQs vs the ELQs

The physical chemical properties of a molecule play an important role in oral bioavailability. For example, a high melting point reflects a higher lattice energy in the crystalline solid which in turn leads to lower drug concentrations in intestinal fluids and reduced absorption. For this reason, the high crystal lattice strength of **ELQ-300** (with a melting point of ∼317 °C) has proven to be a challenge in the clinical development of an acceptable oral formulation for clinical use. Thus, we were interested to compare the crystal lattice energies of HLQ-102 (**1a**) and HLQ-105 (**2a**) vs the corresponding ELQ isomers, i.e., ELQ-300 and ELQ-596, respectively. We compared melting points for these molecules to assess the relative degree of crystallinity in association with changes in chemical structure (Table 12). Both HLQ-102 (**1a**) and HLQ-105 (**2a**) exhibit reduced crystallinity compared to the corresponding isomeric ELQs, i.e., ELQ-300.

**Table 12:** Comparison of the Melting Points for Selected HLQ and ELQ Derivatives^a^

compound	melting point, °C
ELQ-300	317–318^a^
ELQ-596	348.1–351.9^a^
HLQ-102 (1a)	273.5–274.8
HLQ-105 (2a)	303.4–304.5

a Note that these values were taken from the published literature.

### Kinetic Solubility of ELQ-300, HLQ-102 (**1a**) and Prodrugs ELQ-331 and HLQ-130 (29)

Reference compounds were dissolved at 100 μM in the test conditions and the ELQs and HLQs were dissolved at 50 μM in each test condition and these samples were shaken at 1000 rpm for an hour at room temperature. After incubation, the samples were centrifuged to pellet precipitated material, and the supernatant was analyzed by LC–MS to quantitate the amount of dissolved substance using a pre-established standard curve for each molecule. As shown in Table 13, standard control drugs, propranolol, ketoconazole, and tamoxifen exhibited solubilities that ranged from excellent to intermediate to poor aqueous solubility under the conditions of the assay. ELQ-300 exhibited relatively poor aqueous solubility in PBS, but solubility was measurable, albeit low, in simulated intestinal fluids. For reference, the solubility of prodrug ELQ-331 ranged from good to intermediate in media simulating the fasted and fed state. Interestingly, HLQ-102 (**1a**) exhibited an intermediate level of solubility in both fed- and fasted-simulated intestinal fluids (FeSSIF and FaSSIF, respectively) which was slightly improved over these same measures for its prodrug HLQ-130 (**29**). In summary, the solubility of HLQ-102 (**1a**) is improved over its 3-position isomer ELQ-300 in aqueous conditions simulating the fed and fasted state as assessed in vitro at room temperature. The solubility of both compounds in human plasma was roughly the same and was assessed at ∼36 μM.

**Table 13:** Comparison of the Kinetic Solubility of Selected ELQs and HLQs as Well as Three Reference Control Compounds in Standardized Aqueous Conditions at Room Temperature

test article	test system	solubility (μM)	
		mean	RSD
propranolol	PBS (pH 7.4)	**85.15**	0.09
ketoconazole	PBS (pH 7.4)	**36.75**	0.06
tamoxifen	PBS (pH 7.4)	**<0.02**	N/A
ELQ-300	FaSSGF (pH 1.6)	**3.03**	0.04
	FeSSIF (pH 5.8)	**3.26**	0.01
	FaSSIF (pH 6.5)	**1.59**	0.04
	PBS (pH 7.4)	**<0.02**	N/A
	human plasma	**36.05**	0.01
ELQ-331	FaSSGF (pH 1.6)	**7.49**	0.01
	FeSSIF (pH 5.8)	**30.35**	0.11
	FaSSIF (pH 6.5)	**7.57**	0.07
	PBS (pH 7.4)	**<0.02**	N/A
HLQ-102 (1a)	FaSSGF (pH 1.6)	**0.87**	0.04
	FeSSIF (pH 5.8)	**25.55**	0.10
	FaSSIF (pH 6.5)	**29.20**	0.06
	PBS (pH 7.4)	**<0.02**	N/A
	Human plasma	**36.40**	0.04
HLQ-130 (29)	FaSSGF (pH 1.6)	**1.90**	0.16
	FeSSIF (pH 5.8)	**27.90**	0.07
	FaSSIF (pH 6.5)	**11.80**	0.07
	PBS (pH 7.4)	**<0.02**	N/A

### In Vivo Efficacy of HLQ-102 (**1a**) and HLQ-105 (2a) as Well as Two Prodrugs against Murine Malaria

Next, we were interested in testing selected HLQs in vivo against malaria in mice and as before with the ELQs we prepared and tested an alkoxycarbonate prodrug ester of each. First, we assessed the in vivo efficacy of these molecules using a modified Peters 4 Day test. For these experiments the mice were infected on Day 0 with 35,000 infected red cells from a *Plasmodium yoelii* infected donor animal. On Day 1 and for three additional days, each animal received a dose of test agent, dissolved in PEG-400, by oral gavage. The initial dose range was from 0 to 10 mg/kg/day which was narrowed in subsequent experiments to bracket the range needed to accurately determine ED_50_ and ED_90_, as well as the nonrecrudescence dose (NRD). On Day 5, a drop of blood was collected from the tail vein, and a blood smear was prepared, fixed with methanol, and stained with Giemsa. The operator examined each smear microscopically to determine percent parasitemia. Typically, control animals exhibit a level of parasitemia on Day 5 of ∼20%. ED_50_ and ED_90_ values for ELQ-300 and prodrug **ELQ-331** were taken from prior published work for comparison purposes. From two separate experiments (4 mice per group), the estimated ED_50_ and ED_90_ values for HLQ-102 were 0.01 mg/kg/day and 0.075 mg/kg/day which are similar to previously reported values for ELQ-300 (slightly lower for the ED_50_ value and a slightly higher for the ED_90_ value with respect to ELQ-300, Table 14). We also evaluated both **HLQ-102** (**1a**) and its alkoxycarbonate ester prodrug, HLQ-130 (**29**), in the single dose cure (SDC) model in mice also against *P. yoelii*. For both compounds we observed initial parasite clearance by Day 5 following a single oral dose of up to 10 mg/kg; however, the reappearance of parasites in the bloodstream by Day 11 indicated recrudescence.

**Table 14:** Comparative In Vivo Efficacy of **ELQ-300** and **HLQ-102** (**1a**) and Their Methylenoxy-Carbonate Prodrugs^a^

compound	ED50 mg/kg/day	ED90 mg/kg/day	NRD mg/kg/day	single dose cure mg/kg/day
ELQ-300^a^	0.02	0.04	1	>10
ELQ-331^a^	0.02	0.05	1	3
HLQ-102 (1a)	0.01	0.075	3	>10
HLQ-130 (29)	0.01	0.075	3	>10

a These data were taken from a prior publication.^39^

### Pharmacokinetics of HLQ-102 (1a) following a Single Dose of Prodrug HLQ-130 **(29)**

**Mouse PK.** Initially, the pharmacokinetics (PK) of HLQ-102 (**1a**) and HLQ-130 (**29**) were assessed following a single dose administration of HLQ-130 (**29**) at 10 mg/kg by gavage (PO) and 0.5 mg/kg intravenously (IV) to mice (equivalent to a HLQ-102 (**1a**) doses of 8.23 mg/kg (PO) and 0.41 mg/kg (IV)). The levels of HLQ-130 (**29**) are below the lowest limit of quantification (LLOQ) after 1 h of IV administration and 0.25 h of PO administration. These results indicate that HLQ-130 (**29**) is rapidly converted to HLQ-102 (**1a**) in vivo. Plasma concentration versus time curves for HLQ-102 (**1a**) after the administration of HLQ-130 (**29**) are shown in Figure 9 panel A, and the resulting PK parameters are summarized in Table 15. The plasma concentration of HLQ-102 (**1a**) reached a maximum level (*C*_max_) of 345 ng/mL 0.25 h after IV dosing and exhibited an elimination half-life (*T*_1/2_) of 3.16 h. After 10 mg/kg PO the concentration of HLQ-102 (**1a**) peaked at 1513 ng/mL at 8 h after dosing, and the elimination half-life *T*_1/2_ was 4.95 h. The percent bioavailability of HLQ-102 (**1a**) in mice is calculated to be ∼ 85%, if all HLQ-130 (**29**) is converted to only HLQ-102 (**1a**).

**Figure 9:**
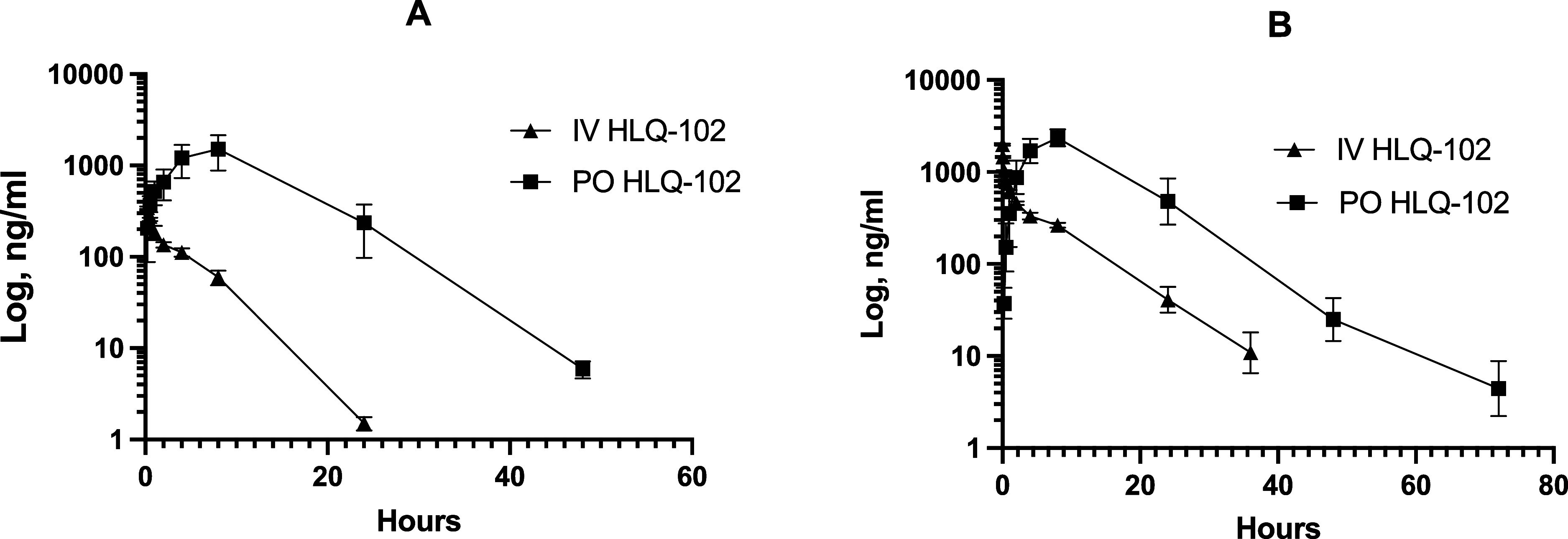
Mean **HLQ-102** (**1a**) (Panel A) 10 mg/kg PO dose 0.5 mg/kg IV dose administered as **HLQ-130** (**29**) in mice, and (Panel B) 10 mg/kg PO dose administered as **HLQ-130** (**29**), 0.5 mg/kg IV dose administered as **HLQ-102** (**1a**) in rats.

**Table 15:** Selected PK Parameters for **HLQ-102** (**1a)** ^a^

PK Parameter Estimate	HLQ-102 (1a) (Dose given as prodrug HLQ-130 (29) in mice)		HLQ-102 (1a) (Dose given as HLQ-102 (1a) in rats)	HLQ-102 (1a) (Dose given as HLQ-130 (29) in rats)
	IV	PO	IV	PO
Dose (mg/kg)	0.41*	8.23*	0.5	8.23*
*T*_max_ (hr)	0.25	8.00	ND	8.00
*C*_max_ (ng/mL)	345	1513	ND	2393
*T*_1/2_ (hr)	3.16	4.95	6.22	7.34
AUC_0‑inf_ (ng*hr/ml)	1485	25116	6360	42314
Vd_ss_ (l/kg)	ND	ND	0.602	ND
CL (l/h/kg)	ND	ND	0.0789	ND
MRT_0‑inf_(hr)	4.87	10.2	7.67	11.9
F (%)	100	85	100	40

a The formulations of **HLQ-130** (**29**) and **HLQ-102** (**1a**) are described in the experimental part. ND, not determined.

**Rat PK.** Given the high level of stability of **HLQ-102** (**1a**) in the presence of rat-derived hepatic microsomes, we also assessed the PK of HLQ-102 (**1a**) and HLQ-130 (**29**) in rats. For these experiments, rats were dosed with HLQ-102 (**1a**) at 0.5 mg/kg IV and HLQ-130 (**29**) at 10 mg/kg PO. Giving the IV dose as the parent drug rather than the prodrug allows for the determination of volume of distribution (Vdss) and clearance (CL) of HLQ-102 (**1a**). Plasma concentration versus time curves for HLQ-102 (**1a**) are shown in Figure 9 panel B, and the resulting PK parameters are summarized in Table 15. After IV administration, the elimination half-life *T*_1/2_ was 6.22 h, with an exposure (AUC_0‑inf_) of 6360 ng*hr/ml, a CL of 0.0789 L/h/kg, and a Vdss of 0.602 L/kg. After 10 mg/kg PO administration, HLQ-102 (**1a**) levels reached a *C*_max_ of 2393 ng/mL 8 h after dosing, with a calculated *T*_1/2_ of 7.34 h, and with an AUC_0‑inf_ of 42314 ng*hr/ml. The percent oral bioavailability of HLQ-102 (**1a**) in rats is 40% with the assumption that all HLQ-130 (**29**) is converted to only **HLQ-102** (**1a**) in the PO arm.

Taking these results together, HLQ-102 (**1a**) exhibits a longer bloodstream elimination half-life in rats (6.22 h IV arm, 7.34 h PO arm) compared to mice (3.16 h IV arm, 4.95 h PO arm), suggesting species-specific differences in elimination kinetics. The extended *T*_max_ observed following orally dosed prodrug HLQ-130 in rats suggests a slow rate of absorption, which may be driven by solubility-limited absorption from the formulation vehicle.

## Discussion and Conclusions

The clinical development of atovaquone (ATV), which targets the Q_o_ site of the *P. falciparum* cyt *bc*_*1*_, validates this enzyme as a target for therapeutic exploitation.^50^ The finding that quinolones such as ELQ-300, which target the distant Q_i_ site of the same enzyme complex, kill malaria parasites at all three life cycle stages, i.e., liver, blood, and vector stages, identifies the cyt *bc*_*1*_ complex as an ideal target since inhibitors of it can be used for prevention and treatment as well as for transmission blocking. Another fact that bolsters its consideration as an ideal target is that resistance mutations in the Cytochrome *b* gene extract a fitness cost on transmission efficiency that renders such parasites essentially biologically dead-end as they mutate into resistance and fall into a well-laid genetic trap.^41,51^ For these various reasons, we believe that the arsenal of agents active against this vital target needs to be expanded to realize the full potential of a strategy that focuses chemotherapy and chemoprevention measures on cyt *bc*_*1*_ to support the worldwide eradication of malaria.

For this study we continued the exploration of 2-position substituted quinolones that was initiated by Ward and O’Neill in 2012.^14^ Here we introduce new streamlined methods for their synthesis that may help to accelerate the investigation of these molecules for broadly different applications. We identify HLQ-102 (**1a**), the 2-position isomer of ELQ-300, as an intriguing molecule from the series. HLQ-102 (**1a**) exhibits greater intrinsic antiplasmodial activity compared to ELQ-300 in vitro but somewhat weaker antimalarial efficacy in vivo in a murine model of malaria infection. The molecule exhibits reduced crystallinity compared to its predecessor and slightly improved solubility in physiologically relevant aqueous media, suggesting that oral bioavailability may be further enhanced through formulation and/or prodrug optimization. HLQ-102 (**1a**) exhibits good metabolic stability in the murine system and excellent stability in the presence of human hepatic microsomes. Bloodstream pharmacokinetics of **HLQ-102** (**1a**) are respectable in both mice (*T*_1/2_ ∼3 to 5 h) and rats (*T*_1/2_ ∼6 to 7 h), albeit not as extended as for ELQ-300 (mice = *T*_1/2_ 15 h; rats = 24 h)^6^ which may relate to differences in volume of distribution, clearance, or altered metabolism in vivo in mice. Further work to modify the chemical structure of HLQ-102 as well as exploring different routes of delivery together with variations in the delivery formulation are being pursued to optimize the in vivo performance of this series in preclinical animal species.

That HLQ-102 (**1a**) and ELQ-300 both target the Q_i_ site of the parasite’s cytochrome bc_1_ complex suggests that the enzyme may have two separate troughs for accommodating the same bulky diphenyl ether side chain at two separate but adjacent positions on the quinolone ring. Like ELQ-300 and the next generation ELQ-596,^49^ HLQ-102 (**1a**) is not cytotoxic toward a reference primary human cell line (HFF) in vitro, even under conditions in which the cells are forced to rely on their mitochondria for survival. And like the other two molecules, HLQ-102 (**1a**) does not inhibit a reference mammalian (bovine) cytochrome bc_1_ complex at concentrations up to 10 μM. The fact that HLQ-102 (**1a**) exhibits low to subnanomolar IC_50_ values against a panel of drug-resistant *P. falciparum* parasites and yet is nontoxic toward HFF cells at concentrations up to 10 μM, indicates an in vitro therapeutic index (IVTI) of over 10,000-fold. Taking these findings together, we believe that HLQ-102 (**1a**), along with potential prodrugs, deserves more in-depth study to optimize its performance in vivo and to evaluate whether its unique physical chemical and ADME properties could be used to advantage in combating malaria.

## Materials and Methods

### Chemical Synthesis Procedures

Unless otherwise stated all chemicals and reagents were from Sigma-Aldrich Chemical Co. in St. Louis, MO (USA), Combi-Blocks, San Diego (CA), or TCI America, Portland (OR) and were used as received. Melting points were obtained in the Optimelt Automated Melting point system from Stanford Research Systems, Sunnyvale, CA (USA). Analytical TLC utilized DC Kieselgel 60F_254_ precoated silica gel plates and spots were visualized under 254 nm UV light. GC–MS was obtained using an Agilent Technologies 7890B gas chromatograph (30 m, DBS column set at 100 °C, 125 °C, 150 °C, or 200 °C for 2 min, then at 30 °C/min to 300 °C with inlet temperature set at 250 °C) with an Agilent Technologies 5977A mass-selective detector operating at 70 eV. Flash chromatography over silica gel column was performed using an Isolera One flash chromatography system from Biotage, Uppsala, Sweden. Unless otherwise noted, the default TLC method as implemented by the machine was used in the gradient of the eluting solvent. ^1^H NMR spectra were obtained using a Bruker 400 MHz Avance NEO NanoBay NMR spectrometer operating at 400.14 MHz. The NMR raw data were analyzed using the iNMR Spectrum Analyst software. ^1^H chemical shifts are reported in parts per million (ppm) relative to internal tetramethylsilane (TMS) standard or residual solvent peak. Coupling constant values (*J*) are reported in hertz (Hz). Decoupled ^19^F operating at 376 MHz was also obtained for compounds containing fluorine (data not shown). HPLC analyses were performed using an Agilent 1260 Infinity instrument with detection at 254 nm and a Phenomenex, Luna 5 μm C8(2) 100 Å reverse phase LC column 150 × 4.6 mm at 40 °C, and eluted with a gradient of A/B at 25%: 75% to A/B at 10%: 90% (A:0.05% formic acid in Milli-Q water, B: 0.05% formic acid in methanol). High-resolution mass spectrometry (HRMS) was performed using a high-resolution (30,000) Thermo LTQ-Orbitrap Discovery hybrid mass spectrometry instrument (San Jose, CA) equipped with an electrospray ionization source operating in the positive or negative ion mode. The Orbitrap was externally calibrated prior to data acquisition allowing accurate mass measurements for [M + H]^+^ ions to be obtained within 4 ppm. All compounds were at least >95% pure for in vitro testing and >98% pure for in vivo testing as determined by GC–MS, ^1^H NMR, and HPLC. For individual synthetic methods, please see Supporting Information.

### Modeling

The structure of the *P. falciparum* cytochrome bc_1_ complex (PfCyt bc_1_) was homology modeled using the experimentally determined cryo-electron microscopy (cryo-EM) structure of the *T. gondii* cytochrome bc_1_ complex (PDB ID: 9I4X)^52^ as a structural template. Lipid molecules resolved in the template structure, including phosphatidylcholine (PC1) and cardiolipin (CDL) located adjacent to the Q_i_ inhibitor binding cavity, were spatially mapped and incorporated into the corresponding positions of the PfCyt bc_1_ model to preserve the native lipid microenvironment surrounding the inhibitor binding site. Homology model construction and structural preparation were performed using molecular operating environment software (MOE;^53^ Chemical Computing Group). Ionizable residues were assigned appropriate protonation states at physiological pH (7.0) based on predicted p*K*_a_ values and local interaction networks, with acidic and basic side chains selectively protonated or deprotonated accordingly. Structure-based protein–ligand docking was conducted using the genetic algorithm (GA) search strategy implemented in Genetic Optimization for Ligand Docking software (GOLD; Cambridge Crystallographic Data Centre, CCDC).^54^ For each ligand, 50 initial conformers were generated to sample conformational space and subsequently energy-minimized using the AMBER ff10 force field.^55^ The resulting docked protein–ligand complexes were scored using the default GOLD empirical scoring function, which estimates binding fitness using interaction terms optimized for GA-driven pose selection. To evaluate the contribution of lipids to inhibitor stabilization at the Q_i_ site, docking simulations were performed under two conditions: (i) in the presence of PC1 and CDL, and (ii) after removal of lipid molecules from the template-derived model. This comparative strategy was designed to assess the role of lipids in modulating inhibitors pose stability and binding fitness at the Q_i_ cavity.

### In Vitro Metabolic Stability Assay

Metabolic stability of selected HLQs was investigated. The compounds were incubated at 37 °C and 1 μM concentration in liver microsomes from multiple species (Corning) for 45 min at a protein concentration of 0.5 mg/mL in potassium phosphate buffer at pH 7.4 containing 1.0 mM EDTA. The metabolic reaction was initiated by addition of NADPH and quenched with ice-cold acetonitrile at 0, 5, 15, 25, and 45 min. The progress of compound metabolism was followed by LC–MS/MS (ESI positive ion, LC–MS/MS-034­(API-6500+) using a C_18_ stationary phase (ACQUITY UPLC BEH C_18_ (2.1 Å∼ 50 mm, 1.7 μm)) and a MeOH/water mobile phase containing 0.25% formic acid and 1 mM ammonium acetate. Imipramine was used as internal standard, and ketanserin was used as a control drug with intermediate stability. Concentration versus time data for each compound were fitted to an exponential decay function to determine the first-order rate constant for substrate depletion, which was then used to calculate the degradation half-life (t_1/2_) and predicted intrinsic clearance value (Cl_int_) from an assumed murine hepatic blood flow of 90 mL/min/kg.

### *P. falciparum* Culture

Laboratory strains of *P. falciparum* were cultured in human erythrocytes by standard methods. The parasites were grown in culture medium with fresh human erythrocytes, maintained at 2% hematocrit at 37 °C in low-oxygen conditions (5% O_2_, 5% CO_2_, 90% N_2_). The culture medium was RPMI 1640 supplemented with 25 mM Hepes buffer, gentamicin sulfate (25 mg/Liter), hypoxanthine (45 mg/Liter), 10 mM glucose, 2 mM glutamine, and 0.5% Albumax II (complete medium). Cultures were maintained at less than 10% parasitemia by transfer of infected cells to fresh erythrocytes and culture medium every 3 or 4 days. The *P. falciparum* strains used in these experiments include the following: D6 (MRA-285/BEI Resources, deposited by Dr. Dennis Kyle) with modest resistance to mefloquine but generally drug sensitive; Dd2 (MRA-150/BEI Resources, deposited by Dr. David Walliker) with resistance to chloroquine, mefloquine and pyrimethamine; D1 is a subclone of Dd2 that was selected for resistance to ELQ-300, and Tm90–C2B was isolated from a patient enrolled in an atovaquone clinical trial in Thailand upon recrudescence after cessation of drug treatment (obtained from Drs. Dennis Kyle and Victor Melendez, WRAIR). Note that the human red cells used for culture of malaria parasites was purchased (without identifying information) from Lampire Biological Laboratories, Pipersville, PA 18947.

### In Vitro Drug Susceptibility Assays–*P. falciparum*

In vitro antiplasmodial activity was assessed using a published SYBR Green I fluorescence-based method.^43^ The drugs were added to 96-well plates using 2-fold serial dilutions in complete medium. The initial range was from 250 nM to 0.25 nM. Asynchronous *P. falciparum* parasites were diluted with uninfected erythrocytes and added to the wells to give a final culture volume of 100 μL at 2% hematocrit and 0.2% parasitemia. The plates were incubated for 72 h at 37 °C. The parasites were then lysed by adding 100 μL of SYBR green I lysis buffer containing 0.2 μL/ml SYBR green I dye (10,000X) in 20 mM Tris (pH 7.5), 5 mM EDTA, 0.008% (wt/vol) saponin, and 0.08% (v/v) Triton X-100. The plates were incubated at room temperature for an hour in the dark. The fluorescence signal, correlating to parasite DNA, was measured using a SpectraMax iD3 iD5Multi-Mode Microplate Reader, with excitation and emission wavelength bands centered at 497 and 520 nm, respectively. The 50% inhibitory concentrations (IC_50_) were determined by nonlinear regression analysis using GraphPad Prism software. Test agents were assayed in quadruplicate, and the results were averaged during analysis to give a final IC_50_ value together with standard deviations and 95% confidence intervals. Atovaquone and ELQ-300 were used as internal controls to verify cross-resistance and parasite strain integrity. If the IC_50_ value fell outside of the initially tested range, then the range was adjusted up or down and the assay was repeated twice.

### In Vitro Plasmodium Liver-Stage Drug Assays

In vitro liver-stage antimalarial activity was evaluated using both a rodent malaria model (*P. berghei* inhibition of liver-stage development assay, ILSDA) and a nonhuman primate malaria model (*P. cynomolgi* liver-stage assay), enabling assessment of prophylactic, schizonticidal, and hypnozonticidal activity.

### Sporozoite Production and Isolation

*P. berghei* sporozoites were obtained from salivary glands of infected Anopheles mosquitoes provided by WRAIR Entomology. Salivary glands were dissected under sterile conditions, mechanically disrupted, and sporozoites were suspended in hepatocyte culture medium immediately prior to infection. *P. cynomolgi* bastianellii (B strain) sporozoites were obtained from *A. dirus* mosquitoes infected at the Armed Forces Research Institute of Medical Sciences (AFRIMS, Thailand). Salivary glands were dissected aseptically, and sporozoites were released by gentle mechanical disruption. Freshly isolated sporozoites were used immediately for hepatocyte infection to preserve viability and infectivity.^56–58^

### Hepatocyte Culture and Sporozoite Infection

For *P. berghei* assays, white 96-well plates (Nunc, Cat. No. 136101) were coated with ECL Cell Attachment Matrix (Millipore, Cat. No. 08-110) diluted 1:50 in HBSS (Gibco, Cat. No. 14175-095). HepG2 cells were cultured in MEM (Gibco, Cat. No. 11900-099) supplemented with sodium bicarbonate, heat-inactivated fetal bovine serum, l-glutamine, nonessential amino acids, insulin, bovine serum albumin, and antibiotics, and seeded at 2.5 × 10^4^ cells per well. *P. berghei* sporozoites were added at 10,000 sporozoites per well and allowed to invade hepatocytes for 3 h, after which extracellular parasites were removed by washing.^59–61^ For *P. cynomolgi* assays, primary cryopreserved simian hepatocytes (donor lots QHO, NLN, CHR; BioIVT, Baltimore, MD, USA) were thawed according to the manufacturer’s instructions and seeded into collagen-coated 384-well plates. Cells were allowed to form confluent monolayers for 24–48 h prior to infection. *P. cynomolgi* sporozoites were added at an approximately 1:1 hepatocyte-to-sporozoite ratio within 48 h postseeding. Automated liquid-handling systems were used for media exchanges and compound additions to minimize variability.^56–58^

### Compound Treatment Regimens

Test compounds were prepared as 11-point, 2-fold serial dilutions, with assay-specific maximum concentrations. For the *P. berghei* ILSDA, compounds were tested at final concentration ranging from 100 nM to 0.098 nM where the compounds were added immediately after invasion and maintained for 48 h [6]. Atovaquone, provided by the Riscoe laboratory, was included as a positive control. For the *P. cynomolgi* liver-stage assay, compounds were tested at final concentrations ranging from 1000 nM to 0.98 nM and were evaluated under two treatment modalities: (i) prophylactic mode, with daily dosing initiated 1 h postinfection and continued for 3 days to assess activity against developing liver schizonts, and (ii) radical-cure mode, with dosing initiated on day 4 postinfection and continued for 4 days to assess activity against dormant hypnozoites. Tafenoquine, atovaquone, KDU691, and maduramicin were included as assay controls as appropriate. Media were replenished daily with fresh compound-containing hepatocyte culture medium.^56–58^

### Fixation, Imaging, and Data Analysis

For *P. cynomolgi* assays, infected cultures were fixed with 4% paraformaldehyde and stained with species-specific anti-*Plasmodium* antibodies followed by fluorescent secondary antibodies, with nuclei counterstained using Hoechst 33342 (Invitrogen). Plates were imaged using an Operetta CLS high-content imaging system, and parasites were classified as schizonts or hypnozoites based on size, morphology, and fluorescence intensity using Harmony software and in-house Python analysis pipelines. Hepatocyte viability was assessed by nuclear counts.^56–58^ For *P. berghei* assays, parasite burden was quantified using a luciferase-based luminescence readout following addition of D-luciferin substrate (Caliper Life Sciences), and luminescence was measured on the PerkinElmer EnSight microplate reader. Both assays were performed with in-plate technical duplicates. *P. berghei* experiments were conducted with two independent biological replicates, whereas *P. cynomolgi* experiments were conducted with three independent biological replicates. For both assays, percent inhibition values were calculated relative to DMSO-treated controls, and half-maximal inhibitory concentrations (IC_50_) were determined by fitting dose–response curves to a four-parameter logistic model using a Python-adapted grid algorithm.^62^

### In Vivo Efficacy against Murine Malaria

The *P. yoelii* 4 day test monitors suppression of patent infection in female CF1 mice, 28–30 g in weight and 8 to 10 weeks of age at the start of each experiment. The test began with the inoculation (iv) of parasitized erythrocytes (3.5 × 10^4^/*Plasmodium yoelli*) (from a donor animal) on the first day of the experiment (D0). After 24 h, test agents (including HLQ-102 (**1a**) and prodrug HLQ-130 (**29**)) were administered daily by gavage for 4 successive days. Initially the 3-biaryl-ELQs were tested at doses of 0.0025, 0.005, 0.01, 0.03, 0.1, 0.3, 1.0, and 10 mg/kg/day, including a vehicle-only (PEG-400) control. After completion of drug treatment, a blood sample was collected (by pricking the tail vein) for determination of parasite burden beginning on the day after the final dose (D5). Percent parasitemia was assessed by direct microscopic analysis of Giemsa-stained blood smears. Drug activity was recorded as % suppression of parasite burden relative to drug-free controls. Animals with observable parasitemia following the experiment were euthanized; animals cleared of parasites from the bloodstream were observed daily with assessment of parasitemia performed weekly until day 30, at which point the animal(s) were scored as cured of infection. Typically, the percentage parasitemia in untreated control animals on Day 5 of the 4 day test is between 20 and 25%. Nonlinear regression analysis was used for objective determination of ED_50_’s and ED_90_’s from the accumulated data as well as the Non-Recrudescence Dose (NRD). The 4 day test protocol was reviewed and approved by the local IACUC board at the Portland VA Medical Center (IRBNET #1860997-9/Local IACUC #6387). Experiments were performed with 4 mice per group to ensure statistical accuracy. Control compounds for these experiments included ELQ-300 and prodrug ELQ-331.

### In Vivo Single-Dose Efficacy against Murine Malaria

The effectiveness of selected HLQ-102 (**1a**) and prodrug HLQ-130 (**29**) was assessed vs the blood stage infection for single dose cures. Mice were infected iv with 3.5 x10^4^ *P. yoelii* infected RBCs as described for the 4 day test above. Drug administration occurred on the day after inoculation (Day 1). Test agents were dissolved in PEG-400 and administered *ig* once. On the fifth day blood films were prepared and % parasitemia was assessed. Animals remaining parasite-free for 30 days after drug administration were considered cured. The initial dosing range was: 0.25, 0.5, 1, 3, 10 mg/kg, including a control. Experiments were performed with 4 mice per group to ensure statistical accuracy. The reported parameter for these studies is the lowest single dose that provides a cure to all 4 animals in the group. ELQ-331 served as a positive control in these studies to directly compare with prodrug HLQ-130 (**29**).

### Purification of Bovine Mitochondria for Assessment of the Inhibition of Bovine Cytochrome bc_1_ Activity

Mitochondrial isolation from bovine heart was carried out using the protocol described in^48^ with slight modifications. Briefly, a heart was obtained at slaughter and kept on ice during transport. It was cleaned of connective tissue, cut into 3 cm cubes, washed with washing solution (0.15 M KCl), and passed through a commercial meat grinder. Ground tissue was suspended in sucrose buffer (0.25 M sucrose, 10 mM Tris, pH 7.6) at a ratio of 200 mL buffer–100 g tissue. The suspension was further homogenized in a Waring blender (30s on, 10 s off, 30s on, high speed), after which the pH of the suspension was adjusted to 7.5 with 1 M Tris. The homogenate was centrifuged to remove cell debris (1000 g, 10 min, 4 °C in Beckman Allegra 6R, GH-3.8 rotor) and the supernatant filtered through two layers of cheesecloth. The filtrate was centrifuged (26,000*g*, 30 min, 4 °C, Beckman L7 ultracentrifuge, Ti70 rotor), the pellet resuspended in 45 mL sucrose buffer and then lysed by two passes of nitrogen cavitation (Parr Instrument Company 4639 cell disruption vessel, 2300 psi). The lysate was again centrifuged (26,000*g*, 30 min, 4 °C) and the supernatant removed. The resulting pellet was resuspended in storage buffer (50 mM tricine, 100 mM KCl, 2 mM NaN_3_, 2% *N*-dodecyl-β-D-maltoside, 30% glycerol, pH 8.0) and the protein concentration determined by the bicinchoninic acid method (ThermoScientific) prior to aliquoting, rapid-freezing in an ethanol/dry ice bath, and storing at −80 °C. The stock protein concentration was 3.85 mg/mL.

### Cytochrome bc_1_ Reduction Assays

These were performed as described previously,^6,49^ substituting azoxystrobin for antimycin A. Compounds were dissolved in DMSO to make 1 mM stock concentrations. Final protein concentration was 1.5 mg in a total volume of 200 μL. Rates of cytochrome bc_1_ reduction for each compound were measured by taking the slope of (Abs_550_-Abs_542_/time over a 7-point dilution series (2-fold from 20 μM) with an eighth control point containing no test compound. Azoxystrobin (10 μM) was used to demonstrate full inhibition of the cytochrome bc_1_ complex.

### Mitochondrial Toxicity of HLQ-102 (**1a**) and HLQ-105 **(2a)**

Human Foreskin Fibroblasts (Primary Dermal Fibroblast Normal/HFF; Human Neonatal, ATCC #PCS-201-010) were acquired from ATCC (PCS-201-010). HFF cells were routinely cultured in complete in Dulbecco’s Modified Eagle Media (DMEM) with 4.5 g/L d-glucose, l-glutamine, and 110 mg/mL sodium pyruvate (Gibco) and supplemented with 10% Fetal Clone 1 serum (Hyclone), 50U/mL penicillin (Gibco), 50 μL/mL streptomycin (Gibco), and 1x GlutaMAX (Gibco). For studies to investigate the potential for mitochondrial toxicity by selected test agents the medium contained galactose rather than glucose to force the cells to produce ATP through oxidative phosphorylation, which in turn makes them sensitive to mitochondrial toxicants. HFF cells were added to each well of a 384-well plate with complete medium and the cells were incubated at 37 °C in a humidified atmosphere of 95% humidity and 5% CO_2_ until a confluent monolayer had been reached in each well across the plate. After this period the culture medium was removed, and the cells were shifted to either glucose- or galactose-containing medium with test agents at concentrations ranging from 0 to 10 μM. Compound dilutions were prepared from DMSO stocks. All plates were incubated for 72 h at 37 °C. After incubation, the contents of each well were removed and 20 μL of TiterGlo 2.0 reagent mix containing luciferase, detergent, and luciferin was added to each well along with 25 μL of culture medium. The luminescence signal was allowed to develop and stabilize at room temperature for 15 min. At this point the plates were read on a luminometer (SpectraMax iD3 iD5Multi-Mode Microplate Reader) and the data processed to yield the percent decrease in ATP production relative to no-drug control values. These values reflect the potential damage caused by mitochondrial toxicants due to reduced intracellular ATP levels caused by interference with the human mitochondrial electron transport chain. Assays were set up in quadruplicate. Rotenone was used as a positive control for mitochondrial toxicity.

### Experimental for Pharmacokinetic Studies

The pharmacokinetics (PK) of **HLQ-102** (**1a**) and HLQ-130 (**29**) was investigated in mice and in rats. The mouse experiment used male CD1 mice between 28 and 30 g and 6–8 weeks old, and the rat experiment used male SD rats between 220 and 230 g and 6–8 weeks old at the start of the study. For mice, the in vivo PK properties of prodrug HLQ-130 (**29**) and its active metabolite, HLQ-102 (**1a**), were evaluated following PO dosing (10 mg/kg of HLQ-130 (**29**), an equivalent dose of 8.23 mg/kg of HLQ-102 (**1a**)) and IV dosing (0.5 mg/kg of HLQ-130 (**29**), an equivalent dose of 0.41 mg/kg of HLQ-102 (**1a**)). For rats, the in vivo PK properties of prodrug HLQ-130 (**29**) and its active metabolite, HLQ-102 (**1a**), were evaluated following PO dosing (10 mg/kg of HLQ-130 (**29**), an equivalent dose of 8.23 mg/kg of HLQ-102 (**1a**)) and IV dosing (0.5 mg/kg of HLQ-102 (**1a**)). The vehicle used for PO dosing was PEG-400 for both species. The formulation used for IV administration in mice was composed of 10% DMSO, 10% Kolliphor Solutol, and 80% saline while for rats it was composed of 10% DMSO, 10% Solutol HS15, and 80% saline. Animals had free access to food and water during the study. Following compound administration, the animals were manually restrained at the designated time points and approximately 110 μL of blood was withdrawn from the facial vein for semiserial sampling into K_2_EDTA tubes. Blood samples were placed on dry ice and centrifuged (3000*g*, 5 min, 4 °C) within 15 min to obtain plasma. An aliquot of 60 μL of plasma was mixed with 6 μL of 10% formic acid (FA) in water [plasma:10% FA (v/v = 10:1)]. Samples were taken at 15, 30 min, and 1, 2, 4, 8, 24, 48, and 72 h. At the outset of the study 18 animals were randomly separated into 2 different groups (PO and IV) of 9 animals each. Blood samples were taken from 3 animals at each time point such that the same group of 3 animals were sampled at every fourth time point throughout the experiment for both PO and IV arms. The plasma concentrations of HLQ-130 (**29**) and HLQ-102 (**1a**) were analyzed by a validated LC–MS/MS method using a Siex Triple Quad 6500 instrument equipped with a Waters AQUITY UPLC BEH C18 column (2.1 × 50 mm, 1.8 μm). Separation was performed by reverse phase chromatography with the following conditions: Mobile Phase A:H_2_O/acetonitrile (ACN)(95%)-0.1%FA-5 mM NH_4_OAc and Mobile Phase B: ACN/H2O(95%)-0.1%FA-5 mM NH_4_OAc with a gradient (%B) of 10% (0.15 min), 50% (0.8 min), 95% (1.80 min), 95% (2.40 min), 10% (10 min) and stop (2.80 min). Diclofenac was used as an internal standard. The flow rate was 0.60 mL/min and the column separation temperature was set to 60 °C. Under these conditions the retention time for HLQ-130 (**29**) was 2.18 min, for HLQ-102 (**1a**) it was 1.71 min, and for diclofenac it was 1.53 min with an LLOQ (lower limit of quantification) of 1 ng/mL. Concentration vs time profiles were analyzed using Phoenix WinNonLin version 8.3.5.340 software as implemented by Certara (Princeton, NJ) to derive the PK values. The dose input of HLQ-130 (**29**) was 10 mg/kg for the PO arm and 0.5 mg/kg for the IV arm. Based on MW and assuming all HLQ-130 (**29**) was converted to **HLQ-102** (**1a**), the estimated dose input of HLQ-102 (**1a**) was 8.23 mg/kg for the PO arm and 0.41 mg/kg for the IV arm. The absolute bioavailability (F) is calculated according to the following equation

$$F=\frac{{AUC}_{0-\inf PO}}{{AUC}_{0-\inf IV}}.\frac{{Dose}_{IV}}{{Dose}_{PO}}$$

## Supplementary Material







## Notes

Molecular formula strings (CSV)
X-ray distance measurements (PDB)
Synthetic methods, ^13^C NMR data, HPLC data, X-ray data, docking scores, liver stage activity (PDF)

## Abbreviations USED

AcOH: acetic acid
ADME: absorption distribution metabolism excretion
AMU: atomic mass unit
ATP: adenosine triphosphate
ATV: atovaquone
AUC_0‑inf_: area under the concentration–time curve, to infinity
CC_50_: concentration that kills 50% of cells
CDL: cardiolipin;
CHCl_3_: chloroform
CL: clearance
Cl_int_: intrinsic clearance
*C*_max_: maximum concentration
cryo-EM: cryo electron microscopy
cyt bc_1_: cytochrome bc_1_
D1: ELQ-300 resistant *Plasmodium falciparum*
D6: drug sensitive *Plasmodium falciparum*
Dd2: multidrug resistant *Plasmodium falciparum*
DMF: dimethylformamide
DMSO: dimethylsulfoxide
ED_50_: dose that decreases parasite load by 50%
ED_90_: dose that decreases parasite load by 90%
EDTA: ethylenediaminetetraacetic acid
EIC: extracted ion chromatograms
ELQ: endochin-like quinolone
ESI: electrospray ionization
EtOH: ethanol
% F: percent bioavailability
FA: formic acid
FaSSIF: fasted simulated intestinal fluid
FeSSIF: fed simulated intestinal fluid
GC-MS: gas chromatography- mass spectrometry
GFP: green fluorescent protein
GOLD: Genetic Optimization for Ligand Docking
HDQ: 1-hydroxy-2-dodecyl-4­(1H)-quinolone
HFF: human foreskin fibroblast
HLQ: HDQ-like quinolone
HPLC: high performance liquid chromatography
HQ: 2-dodecyl-4­(1H)-quinolone
HRMS: high resolution mass spectrometry
IC_50_: concentration that inhibits 50% of cell growth
ILSDA: inhibition of liver-stage development assay
IV: intravenous
IVTI: in vitro therapeutic index
K_2_CO_3_: potassium carbonate
KOAc: potassium acetate
LC-MS: liquid chromatography-mass spectrometry
LLOQ: lower limit of quantification
mCPBA: meta-chloro peroxybenzoic acid
MeOH: methanol
MOE: molecular operating environment
MRT_0‑inf_: mean residence time, infinity
NADPH: nicotinamide adenine dinucleotide phosphate
NaH: sodium hydride
NaOH: sodium hydroxide
ND: not determined
NHP: nonhuman primate
NMR: nuclear magnetic resonance
NRD: nonrecrudescence dose
PBS: phosphate-buffered saline
PC1: phosphatidylcholine;
Pd­(dppf)­Cl_2_: [1′-Bis­(diphenylphosphino)­ferrocene]­dichloropalladium­(II)
Pf: *Plasmodium falciparum*
PfGFP-Luc_con_: *Plasmodium berghei* green fluorescent protein luciferase construct
PK: pharmacokinetics
PO: per oral;
POCl_3_: phosphorus oxychloride
ppm: parts per million
PQS: *Pseudomonas* quorum-sensing
p-TsOH: para-toluene sulfonic acid
Q_i_-site: quinolone reduction site
Q_o_-site: quinolone oxidation site
SAR: structure-activity relationship
SDC: single dose cure
*T*_1/2_: half-life
TBAI: tetra-butyl ammonium iodide
THF: tetrahydrofuran
TLC: thin layer chromatography;
Tm90–C2B: atovaquone-resistant clinical isolate of *Plasmodium falciparum*
*T*_max_: time of maximum concentration
TMS: tetramethyl silane
Vdss: volume of distribution.

## References

[1] WHO World Malaria Report: Addressing Inequity in the Global Malaria Response; World Health Organization: Geneva, Switzerland, 2024.

[2] Bhatt S., Weiss D. J., Cameron E., Bisanzio D., Mappin B., Dalrymple U., Battle K., Moyes C. L., Henry A., Eckhoff P. A. (2015). The effect of malaria control on *Plasmodium
falciparum* in Africa between 2000 and 2015. Nature.

[3] Hemingway J., Ranson H., Magill A., Kolaczinski J., Fornadel C., Gimnig J., Coetzee M., Simard F., Roch D. K., Hinzoumbe C. K. (2016). Averting a malaria disaster:
will insecticide resistance derail malaria control?. Lancet.

[4] Luth M. R., Godinez-Macias K. P., Chen D., Okombo J., Thathy V., Cheng X., Daggupati S., Davies H., Dhingra S. K., Economy J. M. (2024). Systematic
in vitro evolution in *Plasmodium
falciparum* reveals key determinants of drug resistance. Science.

[5] Salzer W., Timmler H., Andersag A. (1948). Über einen neuen, gegen Vogelmalaria
wirksamen Verbindungstypus. Chem. Ber..

[6] Nilsen A., LaCrue A. N., White K. L., Forquer I. P., Cross R. M., Marfurt J., Mather M. W., Delves M. J., Shackleford D. M., Saenz F. E. (2013). Quinolone-3-diarylethers:
a new class of antimalarial
drug. Sci. Transl. Med..

[7] Nilsen A., Miley G. P., Forquer I. P., Mather M. W., Katneni K., Li Y., Pou S., Pershing A. M., Stickles A. M., Ryan E. (2014). Discovery, synthesis,
and optimization of antimalarial 4­(1*H*)-quinolone-3-diarylethers. J. Med.
Chem..

[8] Stickles A.
M., de Almeida M. J., Morrisey J. M., Sheridan K. A., Forquer I. P., Nilsen A., Winter R. W., Burrows J. N., Fidock D. A., Vaidya A. B. (2015). Subtle changes in endochin-like quinolone structure
alter the site of inhibition within the cytochrome bc_1_ complex
of *Plasmodium falciparum*. Antimicrob.
Agents Chemother..

[9] Srivastava I. K., Morrisey J. M., Darrouzet E., Daldal F., Vaidya A. B. (1999). Resistance
mutations reveal the atovaquone-binding domain of cytochrome b in
malaria parasites. Mol. Microbiol..

[10] Srivastava I. K., Rottenberg H., Vaidya A. B. (1997). Atovaquone, a broad spectrum antiparasitic
drug, collapses mitochondrial membrane potential in a malarial parasite. J. Biol. Chem..

[11] Stickles A. M., Smilkstein M. J., Morrisey J. M., Li Y., Forquer I. P., Kelly J. X., Pou S., Winter R. W., Nilsen A., Vaidya A. B. (2016). Atovaquone and ELQ-300 Combination Therapy
as a Novel Dual-Site Cytochrome bc_1_ Inhibition Strategy
for Malaria. Antimicrob. Agents Chemother..

[12] Winter R., Kelly J. X., Smilkstein M. J., Hinrichs D., Koop D. R., Riscoe M. K. (2011). Optimization of
endochin-like quinolones for antimalarial
activity. Exp. Parasitol..

[13] Vallieres C., Fisher N., Antoine T., Al-Helal M., Stocks P., Berry N. G., Lawrenson A. S., Ward S. A., O’Neill P. M., Biagini G. A. (2012). HDQ,
a potent inhibitor of *Plasmodium
falciparum* proliferation, binds to the quinone reduction
site of the cytochrome bc_1_ complex. Antimicrob. Agents Chemother..

[14] Biagini G. A., Fisher N., Shone A. E., Mubaraki M. A., Srivastava A., Hill A., Antoine T., Warman A. J., Davies J., Pidathala C. (2012). Generation of quinolone antimalarials targeting
the *Plasmodium falciparum* mitochondrial respiratory
chain for the treatment and prophylaxis of malaria. Proc. Natl. Acad. Sci. U. S. A..

[15] David
Hong W., Leung S. C., Amporndanai K., Davies J., Priestley R. S., Nixon G. L., Berry N. G., Samar Hasnain S., Antonyuk S., Ward S. A. (2018). Potent Antimalarial
2-Pyrazolyl Quinolone bc (1) (Q­(i)) Inhibitors with Improved Drug-like
Properties. ACS Med. Chem. Lett..

[16] Leung S. C., Gibbons P., Amewu R., Nixon G. L., Pidathala C., Hong W. D., Pacorel B., Berry N. G., Sharma R., Stocks P. A. (2012). Identification,
design and biological evaluation
of heterocyclic quinolones targeting *Plasmodium falciparum* type II NADH:quinone oxidoreductase (PfNDH2). J. Med. Chem..

[17] Nixon G. L., Pidathala C., Shone A. E., Antoine T., Fisher N., O’Neill P. M., Ward S. A., Biagini G. A. (2013). Targeting the mitochondrial
electron transport chain of *Plasmodium falciparum*: new strategies towards the development of improved antimalarials
for the elimination era. Future Med. Chem..

[18] Pidathala C., Amewu R., Pacorel B., Nixon G. L., Gibbons P., Hong W. D., Leung S. C., Berry N. G., Sharma R., Stocks P. A. (2012). Identification,
design and biological evaluation
of bisaryl quinolones targeting *Plasmodium falciparum* type II NADH:quinone oxidoreductase (PfNDH2). J. Med. Chem..

[19] Amporndanai K., Pinthong N., O’Neill P. M., Hong W. D., Amewu R. K., Pidathala C., Berry N. G., Leung S. C., Ward S. A., Biagini G. A. (2022). Targeting the Ubiquinol-Reduction (Q­(i)) Site
of the Mitochondrial Cytochrome bc(1) Complex for the Development
of Next Generation Quinolone Antimalarials. Biology (Basel).

[20] Yang Y., Yu Y., Li X., Li J., Wu Y., Yu J., Ge J., Huang Z., Jiang L., Rao Y. (2017). Target
Elucidation by Cocrystal Structures of NADH-Ubiquinone Oxidoreductase
of *Plasmodium falciparum* (PfNDH2) with Small Molecule
To Eliminate Drug-Resistant Malaria. J. Med.
Chem..

[21] Lane K. D., Mu J., Lu J., Windle S. T., Liu A., Sun P. D., Wellems T. E. (2018). Selection of *Plasmodium falciparum* cytochrome B mutants by putative PfNDH2 inhibitors. Proc. Natl. Acad. Sci. U. S. A..

[22] Gach-Janczak K., Piekielna-Ciesielska J., Waskiewicz J., Krakowiak K., Wtorek K., Janecka A. (2025). Quinolin-4-ones: Methods
of Synthesis
and Application in Medicine. Molecules.

[23] Camps R. (1899). Synthese von
α- und γ-Oxychinolinen. Ber. Dtsch.
Chem. Ges..

[24] Fisyuk A. S., Kostyuchenko A. S., Goncharov D. S. (2020). Camps Reaction and Related Cyclizations. Russ. J. Org. Chem..

[25] de
Souza J. O., Almeida S. M., Souza G. E., Zanini C. L., da Silva E. M., Calit J., Bargieri D. Y., Amporndanai K., Antonyuk S., Hasnain S. S. (2021). Parasitological profiling
shows 4­(1*H*)-quinolone derivatives as new lead candidates
for malaria. Eur. J. Med. Chem. Rep..

[26] Gould R. G., Jacobs W. A. (1939). The Synthesis of Certain Substituted
Quinolines and
5,6-Benzoquinolines. J. Am. Chem. Soc..

[27] Conrad M., Limpach L. (1887). synthesen von Chinolinderivaten
mittelst Acetessigester. Ber. Dtsch. Chem. Ges..

[28] Pou S., Dodean R. A., Frueh L., Liebman K. M., Gallagher R. T., Jin H., Jacobs R. T., Nilsen A., Stuart D. R., Doggett J. S. (2021). A New
Scalable Synthesis of ELQ-300, ELQ-316, and other Antiparasitic
Quinolones. Org. Process Res. Dev..

[29] Yang Y., Cao L., Gao H., Wu Y., Wang Y., Fang F., Lan T., Lou Z., Rao Y. (2019). Discovery, Optimization, and Target
Identification of Novel Potent Broad-Spectrum Antiviral Inhibitors. J. Med. Chem..

[30] Yang Y., Tang T., Li X., Michel T., Ling L., Huang Z., Mulaka M., Wu Y., Gao H., Wang L. (2021). Design, synthesis, and biological evaluation
of multiple
targeting antimalarials. Acta Pharm. Sin. B.

[31] Somanathan R., Smith K. M. (1981). Synthesis of some 2-alkyl-4-quinolone
and 2-alkyl-4-methoxyquinoline
alkaloids. Journal of Heterocyclic Chemistry.

[32] Szamosvári D., Reichle V. F., Jureschi M., Böttcher T. (2016). Synthetic
quinolone signal analogues inhibiting the virulence factor elastase
of *Pseudomonas aeruginosa*. Chem. Commun..

[33] Piochon M., Coulon P. M. L., Caulet A., Groleau M.-C., Déziel E., Gauthier C. (2020). Synthesis and Antimicrobial Activity of Burkholderia-Related
4-Hydroxy-3-methyl-2-alkenylquinolines (HMAQs) and Their N-Oxide Counterparts. J. Nat. Prod..

[34] Mix A. K., Nguyen T. H. N., Schuhmacher T., Szamosvari D., Muenzner P., Haas P., Heeb L., Wami H. T., Dobrindt U., Delikkafa Y. O. (2025). A quinolone N-oxide
antibiotic selectively targets *Neisseria gonorrhoeae* via its toxin-antitoxin system. Nat. Microbiol..

[35] Reen F. J., Clarke S. L., Legendre C., McSweeney C. M., Eccles K. S., Lawrence S. E., O’Gara F., McGlacken G. P. (2012). Structure-function analysis of the C-3 position in
analogues of microbial behavioural modulators HHQ and PQS. Org. Biomol. Chem..

[36] Yang Y., Yu Y., Li X., Li J., Wu Y., Yu J., Ge J., Huang Z., Jiang L., Rao Y. (2017). Target
Elucidation by Cocrystal Structures of NADH-Ubiquinone Oxidoreductase
of *Plasmodium falciparum* (PfNDH2) with Small Molecule
To Eliminate Drug-Resistant Malaria. J. Med.
Chem..

[37] Pou S., Winter R. W., Dodean R. A., Liebman K., Li Y., Mather M. W., Nepal B., Nilsen A., Handford M. J., Riscoe T. M. (2024). 3-Position Biaryl Endochin-like Quinolones
with Enhanced Antimalarial Performance. ACS
Infect. Dis..

[38] Ton T. M. U., Tejo C., Tiong D. L. Y., Chan P. W. H. (2012). Copper­(II) Triflate
Catalyzed Amination and Aziridination of 2-Alkyl Substituted 1,3-Dicarbonyl
Compounds. J. Am. Chem. Soc..

[39] Frueh L., Li Y., Mather M. W., Li Q., Pou S., Nilsen A., Winter R. W., Forquer I. P., Pershing A. M., Xie L. H. (2017). Alkoxycarbonate Ester Prodrugs of Preclinical Drug Candidate ELQ-300
for Prophylaxis and Treatment of Malaria. ACS
Infect. Dis..

[40] Pal A. C., Renard I., Singh P., Vydyam P., Chiu J. E., Pou S., Winter R. W., Dodean R., Frueh L., Nilsen A. C. (2022). *Babesia duncani* as a Model Organism to Study the
Development, Virulence, and Drug Susceptibility of Intraerythrocytic
Parasites In Vitro and In Vivo. J. Infect. Dis..

[41] Balta V. A., Stiffler D., Sayeed A., Tripathi A. K., Elahi R., Mlambo G., Bakshi R. P., Dziedzic A. G., Jedlicka A. E., Nenortas E. (2023). Clinically
relevant atovaquone-resistant human
malaria parasites fail to transmit by mosquito. Nat. Commun..

[42] Bennett T. N., Paguio M., Gligorijevic B., Seudieu C., Kosar A. D., Davidson E., Roepe P. D. (2004). Novel,
rapid, and inexpensive cell-based
quantification of antimalarial drug efficacy. Antimicrob. Agents Chemother..

[43] Smilkstein M., Sriwilaijaroen N., Kelly J. X., Wilairat P., Riscoe M. (2004). Simple and
inexpensive fluorescence-based technique for high-throughput antimalarial
drug screening. Antimicrob. Agents Chemother..

[44] Esser L., Zhou F., Zeher A., Wu W., Huang R., Yu C. A., Lane K. D., Wellems T. E., Xia D. (2023). Structure
of complex III with bound antimalarial agent CK-2–68 provides
insights into selective inhibition of *Plasmodium* cytochrome
bc(1) complexes. J. Biol. Chem..

[45] MacLean A. E., Shikha S., Ferreira
Silva M., Gramelspacher M. J., Nilsen A., Liebman K. M., Pou S., Winter R. W., Meir A., Riscoe M. K. (2025). Structure,
assembly
and inhibition of the *Toxoplasma gondii* respiratory
chain supercomplex. Nat. Struct. Mol. Biol..

[46] Marroquin L. D., Hynes J., Dykens J. A., Jamieson J. D., Will Y. (2007). Circumventing
the Crabtree effect: replacing media glucose with galactose increases
susceptibility of HepG2 cells to mitochondrial toxicants. Toxicol. Sci..

[47] Crouch S. P., Kozlowski R., Slater K. J., Fletcher J. (1993). The use of ATP bioluminescence
as a measure of cell proliferation and cytotoxicity. J. Immunol. Methods.

[48] Liao P. C., Bergamini C., Fato R., Pon L. A., Pallotti F. (2020). Isolation
of mitochondria from cells and tissues. Methods
Cell Biol..

[49] Pou S., Winter R. W., Dodean R. A., Liebman K., Li Y., Mather M. W., Nepal B., Nilsen A., Handford M. J., Riscoe T. M. (2024). 3-Position
Biaryl Endochin-like Quinolones
with Enhanced Antimalarial Performance. ACS
Infect. Dis..

[50] Hudson A. T. (1993). Atovaquone
- a novel broad-spectrum anti-infective drug. Parasitol. Today.

[51] Goodman C. D., Siregar J. E., Mollard V., Vega-Rodriguez J., Syafruddin D., Matsuoka H., Matsuzaki M., Toyama T., Sturm A., Cozijnsen A. (2016). Parasites resistant to the antimalarial atovaquone fail to transmit
by mosquitoes. Science.

[52] MacLean A. E., Shikha S., Ferreira Silva M., Gramelspacher M. J., Nilsen A., Liebman K. M., Pou S., Winter R. W., Meir A., Riscoe M. K. (2025). Structure,
assembly
and inhibition of the *Toxoplasma gondii* respiratory
chain supercomplex. Nat. Struct. Mol. Biol..

[53] Molecular Operating Environment (MOE) . 02 Chemical Computing Group ULC, 1010; Sherbooke St. West, Suite #910: Montreal, QC, Canada, 2022 H3A 2R7, 2023.

[54] Verdonk M. L., Cole J. C., Hartshorn M. J., Murray C. W., Taylor R. D. (2003). Improved
protein–ligand docking using GOLD. Proteins:
Struct., Funct., Bioinf..

[55] Wang J., Wolf R. M., Caldwell J. W., Kollman P. A., Case D. A. (2004). Development
and testing of a general amber force field. J. Comput. Chem..

[56] Dodean R. A., Li Y., Zhang X., Caridha D., Madejczyk M. S., Jin X., Dennis W. E., Chetree R., Kudyba K., McEnearney S. (2025). Development of Next-Generation Antimalarial Acridones with Radical
Cure Potential. J. Med. Chem..

[57] Pottenger A. E., Roy D., Srinivasan S., Chavas T. E. J., Vlaskin V., Ho D. K., Livingston V. C., Maktabi M., Lin H., Zhang J. (2024). Liver-targeted
polymeric prodrugs delivered subcutaneously improve
tafenoquine therapeutic window for malaria radical cure. Sci. Adv..

[58] Roth A., Maher S. P., Conway A. J., Ubalee R., Chaumeau V., Andolina C., Kaba S. A., Vantaux A., Bakowski M. A., Thomson-Luque R. (2018). A comprehensive model for assessment of liver
stage therapies targeting *Plasmodium vivax* and *Plasmodium falciparum*. Nat. Commun..

[59] Janse C. J., Franke-Fayard B., Mair G. R., Ramesar J., Thiel C., Engelmann S., Matuschewski K., Gemert G. J. v., Sauerwein R. W., Waters A. P. (2006). High efficiency transfection of *Plasmodium
berghei* facilitates novel selection procedures. Mol. Biochem. Parasitol..

[60] Kumar A., Li Y., Zhang X., Gaddam M., Caridha D., Madejczyk M. S., Jin X., Dennis W. E., Chetree R., Blount C. (2025). Optimization
of Prodiginines as Single-Dose Curative Antimalarials. J. Med. Chem..

[61] Ploemen I. H., Prudencio M., Douradinha B. G., Ramesar J., Fonager J., van Gemert G. J., Luty A. J., Hermsen C. C., Sauerwein R. W., Baptista F. G. (2009). Visualisation and quantitative analysis of
the rodent malaria liver stage by real time imaging. PLoS One.

[62] Wang Y., Jadhav A., Southal N., Huang R., Nguyen D. T. (2010). A grid
algorithm for high throughput fitting of dose-response curve data. Curr. Chem. Genomics.

