# Supplementary material for: Revisiting 2‑Substituted-4(1H)‑Quinolones for Targeting the Plasmodium falciparum Cytochrome bc1 Complex
Source: J Med Chem. 2026 Jun 10;69(12):14114–39. doi: 10.1021/acs.jmedchem.5c03295 (PMC13312456; doi:10.1021/acs.jmedchem.5c03295)
Supplement: Supplementary file 3 [file jm5c03295_si_003.pdf]

Supporting Information

Title: Revisiting 2-Substituted-4(1*H*)-Quinolones for Targeting the *Plasmodium falciparum* Cytochrome bc_1 Complex

Authorship: Sovitj Pou,[1,*] Katherine M. Liebman,[1] Rolf W. Winter,[1] Aaron Nilsen,[1,2] Yuexin Li,[1] Isaiah N. Lyche,[1] Teresa M. Riscoe,[1,3] Jemma Montgomery,[1] Max J. Gramelspacher,[1] Rozalia A. Dodean,[1] Lev N. Zakharov,[4] Binod Nepal,[5] Jane X. Kelly,[1] Martin J. Smilkstein,[1] Sandhya Kortagere,[5] Akhil Vaidya,[5] P. Holland Alday,[1,6] J. Stone Doggett,[1,6] Karl Kudyba,[7,8] Norma Roncal,[7,8] Kutub Ashraf,[7,8] Susan Leed,[7,8] Patricia J. Lee,[7] Michael S. Madejczyk,[7] Alison Roth,[7,8] and Michael K. Riscoe[1,3]*

Affiliations:

[1]VA Portland Healthcare System, 3710 SW US Veterans Hospital Road, Portland, Oregon 97239

[2]Department of Chemical Physiology and Biochemistry, Oregon Health & Science University, 3181 SW Sam Jackson Park Road, Portland, Oregon 97239

[3]Department of Microbiology and Molecular Immunology, Oregon Health & Science University, 3181 SW Sam Jackson Park Road, Portland, Oregon 97239

[4]Center for Advanced Materials Characterization in Oregon (CAMCOR), Eugene, Oregon, 97403, United States

[5]Department of Microbiology and Immunology, Drexel University College of Medicine, 2900 Queen Lane, Philadelphia, PA 19129

[6]School of Medicine Division of Infectious Diseases, Oregon Health & Science University, 3181 SW Sam Jackson Park Road, Portland, Oregon 97239

[7]Experimental Therapeutics Branch, CIDR, Walter Reed Army Institute of Research, Silver Spring, Maryland 20910, USA

[8]Integrated Pathogen Therapeutics Department, CIDR, Walter Reed Army Institute of Research, Silver Spring, Maryland 20910, USA

Corresponding Authors:

Sovitj Pou, Ph.D., PharmD, VA Medical Center, 3710 SW US Veterans Hospital Rd., Portland, Oregon 97239; USA, sovitjpou@gmail.com

Michael Riscoe, Ph.D., VA Medical Center, 3710 SW US Veterans Hospital Rd., Portland, Oregon 97239; USA, riscoem@ohsu.edu

Table of Contents:

Synthetic Methods……………………………………………………………………………………………………Page S3

NMR Data……………………………………………………………………………………………………Page S29

HPLC Data……………………………………………………………………………………………………Page S37

X-RAY Data……………………………………………………………………………………………………Page S41

Table S1. GOLD docking scores……………………………………………………………………………Page S45

Figure S1. Dose–response prophylactic in vitro liver stage activity……………………………Page S46

Figure S2. Dose–response radical cure in vitro liver stage activity……………………………Page S47

Figure S3. Distance measurements……………………………………………………………………Page S48

Figure S4. Distance measurements……………………………………………………………………Page S48

Figure S5. Distance measurements……………………………………………………………………Page S49

Figure S6. Distance measurements……………………………………………………………………Page S49

References……………………………………………………………………………………………………Page S50

Synthetic Methods:

Unless otherwise stated, all chemicals and reagents were from Sigma-Aldrich Chemical Company in St. Louis, MO (USA), Combi-Blocks, San Diego (CA), or TCI America, Portland (OR) and were used as received. *meta*-Chloroperoxybenzoic acid (mCPBA) was purchased from Sigma-Aldrich Chemical Company in St. Louis, MO (USA) and was purified and stored at 0°C before use according to the method of Armarego and Lin Chai[1]. Ethyl 3-ketocaprate (**4**) was prepared by published methods[2, 3]. Ethyl 3-keto-2-methylcaprate (**7**) was obtained by generating the anion of **4** in anhydrous THF with NaH, followed by methylation with 1.0 equivalent of methyl iodide, in a manner analogous to reported methods[4]. These esters were used as crude products without further purification. Melting points were obtained using an Optimelt Automated Melting Point system from Stanford Research Systems (Sunnyvale, CA, USA). Analytical TLC utilized Merck 60F-254 250 micron precoated silica gel plates and spots were visualized under 254 nm UV light. GC-MS spectra were obtained using an Agilent Technologies 7890B gas chromatograph (30 m, DBS column set to temperatures between 75°C–200°C for 2 min, then increased by 30°C/min to 300°C with inlet temperature set at 250°C) with an Agilent Technologies 5977A mass-selective detector operating at 70 eV. Flash chromatography was performed using an Isolera One flash chromatography system from Biotage (Uppsala, Sweden), using silica gel. For purifications where the eluting solvent gradient is not specified below, the eluting solvent gradient used for chromatography was calculated by the instrument based on TLC methods. ^{1}H-NMR spectra were obtained using a 400 MHz Avance NEO NanoBay NMR spectrometer from Bruker (Billerica, MA, USA) operating at 400.14 MHz. NMR raw data were analyzed using the iNMR Spectrum Analyst software or Bruker TopSpin. ^{1}H chemical shifts are reported in parts per million (ppm) relative to internal tetramethylsilane (TMS) standard or residual solvent peak. Coupling constant values (*J*) are reported in hertz (Hz). Decoupled ^{19}F operating at 376 MHz was also obtained for compounds containing fluorine. HPLC analyses were performed using an Agilent 1260 Infinity instrument with detection at 254 nm and a Phenomenex, Luna® 5 µm C8(2) 100 Å reverse phase LC column 150 x 4.6 mm at 40°C, and eluted with a gradient of A/B at 25%:75% to A/B at 10%:90% (A: 0.05% formic acid in milliQ water, B: 0.05% formic acid in methanol). High-resolution accurate mass (HRAM) mass spectrometry was performed using a Orbitrap IQ-X Tribrid Mass Spectrometer from Thermo Fisher Scientific (Waltham, MA, USA) with electrospray ionization (ESI) under full scan data acquisition from *m/z* 200-2000. Either electrospray negative ionization mode [M-H]- ions or positive ionization mode [M+H]+ ions were performed, allowing mass measurements to be obtained within 4 ppm. All compounds were at least >95% pure for in vitro and in vivo testing as determined by ^{1}H-NMR and HPLC, as well as GC-MS when appropriate.

General procedure A for the preparation of Schiff bases (5, 8a, 8b, 13, 15a-15k, 18, 21a-e and 21i) and for their cyclization via the Conrad-Limpach reaction (1a-1k, 6, 9a, 9b, 14, 19, 22a-e and 22i): An aniline with the desired substitution was combined with an appropriate 3-aryl or 3-alkyl substituted β-keto ester and a catalytic amount of *para*-toluenesulfonic acid monohydrate (*p*-TsOH•H_2O) in benzene or cyclohexane. This mixture was stirred and heated at reflux using a Dean-Stark trap for a period ranging from three to twenty-one days, monitored by GC-

MS. The solvent was removed under reduced pressure with warming to afford the crude Schiff base, which was used without purification or analysis in the ensuing Conrad-Limpach cyclization reaction. The Schiff base, sometimes diluted with hot Dowtherm A (a eutectic mixture of diphenyl ether and biphenyl) to render it less viscous (see individual syntheses), was added to boiling Dowtherm A (255°C) over five to eight minutes. (Note: Dowtherm A expands significantly upon heating. Volumes of Dowtherm A given below are the volumes measured when cold.) After addition was complete, the reaction mixture was boiled for a seven to ten minutes and allowed to cool, with stirring, to room temperature. Hexanes was added, followed by stirring, to aid precipitation. In some cases, this resulted in a filterable solid, which was recovered by vacuum filtration, rinsing with ethyl acetate and/or acetone. In other cases, a semisolid or oil formed. In those instances, the hexane was decanted (after settling) and the residue was then stirred with ethyl acetate, resulting in a solid precipitate that was recovered by vacuum filtration, rinsing with ethyl acetate and/or acetone. It was usually necessary to recrystallize the resulting products to obtain the pure compounds, as noted below. For several compounds, it was necessary to purify using chromatography (see individual syntheses).

General Procedure B for the preparation of 4-chloro quinolines (20, 23a-e and 23i). To the appropriate 4(1H)-quinolone (1 eq) suspended in chloroform was added phosphorus oxychloride ($POCl_3$, 3.0 eq), and the resulting mixture was stirred at reflux for two to five days. After cooling, the reaction mixture was poured into an ice bath (sometimes diluting with additional chloroform, see individual syntheses) and stirred vigorously to mix the layers. The mixture was then made basic with 50% aqueous sodium hydroxide and further stirred to mix the layers, at the end of which time the pH was confirmed to still be basic. The layers were separated, and the aqueous layer was further extracted with chloroform. The pooled organic layers were rinsed with brine, dried ($MgSO_4$), and evaporated under reduced pressure with warming. In general, the desired products thus afforded were sufficiently pure for use in the next reaction, but if necessary, they were recrystallized from ethyl acetate.

General Procedure C for Suzuki Coupling (24a-24e and 24i). The appropriate 4-chloroquinoline (1 eq), 4-(trifluoromethoxy) benzylboronic acid pinacol ester (2 eq), and K_2CO_3 (2 eq) were combined in toluene/dioxane/water (10:1:1). The reaction mixture was stirred while bubbled with argon for 15 minutes, and $Pd(dppf)Cl_2$ (0.1 eq) was added. Argon was bubbled for another 15 minutes at room temperature, and then the reaction was capped and heated to 90°C for 35 minutes under positive argon pressure. Argon pressure was removed and the reaction mixture was stirred for 24 hours at 90°C. The reaction was cooled to room temperature, extracted with ethyl acetate and water, dried over $MgSO_4$ and filtered. Solvent removed in vacuo, and the product was isolated using flash chromatography with a gradient of 0% to 20% ethyl acetate in hexanes.

General procedure D for Suzuki Coupling (26, 28a-e, 28i and 29). The appropriate 4-chloroquinoline (1 eq) and *para- or meta*-(trifluoromethoxy)phenyl)boronic acid (1.05-1.2 eq) were combined in *N*,*N*-dimethylformamide (DMF), followed by the addition of 2 M aqueous potassium carbonate (2 eq). The stirred reaction mixture was then degassed by bubbling argon through a glass tube inserted under the liquid surface for 20-30 minutes at room

temperature. [1,1'-bis(Diphenylphosphino)ferrocene]-dichloropalladium (II) (Pd(dppf)Cl_2, 0.05 eq) was added, and the reaction was heated at 80°C under an atmosphere of argon for a period of time ranging from 20 hours to 4 days. The cooled reaction mixture was vacuum filtered to remove solids, followed by concentration of the filtrate under reduced pressure with heating. The resulting solid or semisolid was taken up in dichloromethane (DCM) and again vacuum filtered. The evaporated filtrate was purified by automated flash chromatography on silica gel, eluting with a gradient of hexanes/ethyl acetate as described below, to afford the desired product.

General Procedure E for deprotection of 4-chloroquinolines to form 4(1H)-quinolone final products (2a-2e, 2i, 25a-25e, 25i, 27 and 30). The appropriate 4-chloroquinoline was stirred with anhydrous potassium acetate (KOAc) (7.2-10 eq) and glacial acetic acid (AcOH) at 110-120°C in a loosely capped reaction vial for 20-22 hours. The reaction mixture was poured into water, generally without cooling (except as noted). The resulting precipitate was collected by vacuum filtration, rinsing with excess water followed by acetone, DCM, and/or hexanes, followed by air drying to afford the desired products, which were obtained in satisfactory purity without the need for recrystallization.

General Procedure F for the synthesis of alkoxy ester pro-drugs (31 and 34): The appropriate 4(1H)-quinolone was combined with tetrabutylammonium iodide (TBAI) (2.0 eq) and anhydrous K_2CO_3 (2.0 eq) in DMF, followed by the addition of chloromethyl ethyl carbonate (2.0 eq). The reaction was allowed to stir at 60°C, sealed with a needle-vented septum, for 21 hours to 2 days. The cooled reaction mixture was vacuum filtered to remove solids, and the filtrate was concentrated under reduced pressure with heating. The residue was swirled with ethyl acetate, resulting in the formation of a precipitate, which was removed by vacuum filtration. The filtrate (containing the product) was evaporated under reduced pressure and purified by automated flash chromatography on silica, eluting with gradients of hexanes/ethyl acetate, to afford the desired products. In one case it was necessary to effect further purification by crystallization from hexanes (see **34** below).

General Procedure G for the synthesis of quinoline *N*-oxides (32 and 35): To a stirred solution of the appropriate 4-O-alkoxy ester quinoline (**31** or **34**) in chloroform ($CHCl_3$), *meta*-chloroperbenzoic acid (*m*CPBA) was added. The solution was heated at 90°C for 18-24 hours. After cooling to room temperature, the yellow solution was evaporated to dryness under reduced pressure and purified by flash chromatography eluting with gradients of hexane/ethyl acetate.

General Procedure H for the hydrolysis of quinoline *N*-oxide alkoxy ester quinolines to *N*-hydroxy 4(1H)-quinolones (33 and 36): A stirred solution of the appropriate 4-O-alkoxy carbonate quinoline *N*-oxide in methanol/10% aqueous NaOH (4/1) was heated for 2 hours. The mixture was concentrated to dryness under reduced pressure, water was added, and the mixture was filtered. The resulting solid was washed with water and DCM and then air-dried.

Ethyl 2-methyl-3-oxo-3-(4-(4-(trifluoromethoxy)phenoxy)phenyl)propanoate (1a, HLQ-102). Following General Procedure A, the Schiff base **15a** was obtained by refluxing **12b** (11.46 g, 30 mmol, 1.0 eq), 4-chloro-3-methoxyaniline **3a** (4.74 g, 30 mmol, 1.0 eq), and *p*-TsOH·H_2O (285 mg, 1.5 mmol, 0.05 eq) in benzene (50 ml) for 72 hours. The residual black oil (**15a**, 15.2 g) diluted in Dowtherm A (5 ml), was added with stirring to Dowtherm A (75 ml) at boiling temperature for over 2 minutes; then kept at boiling temperature for another 7 minutes. After cooling, hexanes (400 ml) was added while stirring to precipitate the product. Stirring was continued for another 0.5 hours. The solid was filtered, rinsed with ethyl acetate (10 ml), and air-dried to give **1a** (**HLQ-102**) as a yellowish solid (4.30 g, 31%, mp 273.5-274.8 °C (dec)), ^{1}H-NMR (400 MHz; DMSO-d_6): δ 11.57 (s, 1H), 8.04 (s, 1H), 7.62-7.59 (m, 2H), 7.48-7.44 (m, 2H), 7.24-7.19 (m, 5H), 3.92 (s, 3H), 1.90 (s, 3H). ^{13}C-NMR (101 MHz; DMSO-d_6): δ 175.7, 157.7, 157.0, 155.5, 147.2, 144.6, 140.2, 131.5, 130.7, 126.2, 123.68, 123.62, 121.0, 118.9, 118.5, 118.1, 114.9, 100.2, 56.7, 12.6. HRMS calculated for C24H17ClF3NO4 [M + H]+ = 476.08709, observed for [M + H]+ = 476.08733.

5,7-Difluoro-3-methyl-2-(4-(4-(trifluoromethoxy)phenoxy)phenyl)quinolin-4(1*H*)-one (1b, HLQ-127). Following General Procedure A, the Schiff base **15b** was obtained by refluxing **12b** (11.46 g, 30 mmol, 1.0 eq), 3,5-difluoroaniline **3b** (4.06 g, 31.5 mmol, 1.05 eq), and *p*-TsOH·H_2O (570 mg, 3.0 mmol, 0.1 eq) in benzene (50 ml) for 72 hours. The resulting black oil (**15b**, 15.4 g) was diluted with 5 ml of Dowtherm A and added to 80 ml boiling Dowtherm A over 3 minutes, heating for a total of 8 minutes. The cooled reaction mixture was stirred briefly with hexanes (150 ml), and the precipitate was recovered by filtration, washing with hexanes (3 x 50 ml). Obtained was 8.55 g of the pure product **1b** (**HLQ-127**) as a beige powder (57% yield), ^{1}H-NMR (400 MHz; DMSO-d6): δ 11.66 (s, 1H), 7.62-7.58 (m, 2H), 7.48-7.45 (m, 2H), 7.25-7.20 (m, 4H), 7.15 (ddd, *JH* = 2.4, *JF* = 10.0, 1.3 Hz, 1H), 7.03 (ddd, *JH* = 2.5 Hz, *JF*=12.1, 9.6, 1H), 1.85 (s, 3H); ^{19}F-NMR (376 MHz; DMSO): δ -57.1 (s), -105.5 (m), -108.7 (m). HRMS calculated for C23H14F5NO3 [M + H]+ = 448.09666, observed for [M + H]+ = 448.09687.

3-Methyl-2-(4-(4-(trifluoromethoxy)phenoxy)phenyl)quinolin-4(1H)-one (1c, HLQ-125). Using General Procedure A, a mixture of 5.00 g (13.1 mmol) **12b**, 1.21 g of aniline **3c** (1.00 eq), and TosOH·H_2O (50 mg) in benzene (75 ml) was heated at reflux for 14 days. The Schiff base **15c** was diluted in Dowtherm A (5 ml) and added to boiling Dowtherm A (75 ml) over 2 minutes, and then kept at a boil for another 7 minutes, after which time it was allowed to cool with stirring maintained. The cooled solution was diluted with hexanes (to a total volume of 500 ml) and stirred for 15 minutes. The precipitate was filtered off by suction, washing with 50 ml 4:1 hexanes/ethyl acetate (50 ml). A beige powder (2.23 g) was obtained. It was heated with ethyl acetate (20 ml), and acetone (0.5 ml) was added to achieve complete dissolution. The solution was covered and set aside for 1.5 hours, when the product **1c** (**HLQ-125**) was filtered off. It was obtained as cream-colored shiny plates (1.63 g, 30% yield), ^{1}H-NMR (400 MHz; DMSO-d_6): δ 11.58 (s, 1H), 8.14-8.10 (m, 1H), 7.63-7.60 (m, 4H), 7.48-7.46 (m, 2H), 7.30 (ddd, *J*

= 8.1, 5.1, 3.0 Hz, 1H), 7.25-7.21 (m, 4H), 1.92 (s, 3H); ^{19}F-NMR (376 MHz; DMSO): δ -57.1 (s). HRMS calculated for C23H16F3NO3 [M + H]+ = 412.11550, observed for [M + H]+ = 412.11568.

7-Methoxy-3-methyl-2-(4-(4-(trifluoromethoxy)phenoxy)phenyl)quinolin-4(1*H*)-one (1d, HLQ-135). Using General Procedure A, *meta*-anisidine **3d** (1.61 g, 0.013 mol), **12b** ethyl 2-methyl-3-oxo-3-(4-(4-(trifluoromethoxy)phenoxy)phenyl)propanoate (5.00 g, 0.013 mole, 1.0 eq) and a catalytic amount of *p*-TsOH•H_2O (about 0.23 g) were heated in benzene (70 ml) for six days. The crude Schiff base **15d** (an inhomogeneous ochre oil) was taken up in hot Dowtherm A (10 ml, then 5 ml to rinse) and added to boiling Dowtherm A (100 ml) over the course of 7 minutes, heating for a total of 15 minutes. The cooled reaction mixture was stirred 5 minutes with hexanes (300 ml). After settling, the supernatant was decanted from the resulting brown, thick oil, which was swirled with ethyl acetate (75 ml) and recovered by vacuum filtration, rinsing with ethyl acetate (125 ml) followed by acetone (8 ml). The crude product (a yellowish cream powder, 0.65 g) was recrystallized from DMF (4 ml), affording the desired product **1d** (**HLQ-135**) as pale, dull yellow crystals (0.31 g, 5.4% yield over two steps from *meta*-anisidine), ^{1}H-NMR (400 MHz; DMSO-d_6): δ 11.39 (s, 1H), 8.02 (d, *J* = 9.0 Hz, 1H), 7.61-7.58 (m, 2H), 7.48-7.45 (m, 2H), 7.24-7.21 (m, 4H), 7.02 (d, *J* = 2.4 Hz, 1H), 6.90 (dd, *J* = 9.0, 2.5 Hz, 1H), 3.83 (s, 3H), 1.89 (s, 3H). ^{19}F-NMR (376 MHz; DMSO): δ -57.1. HRMS calculated for C24H18F3NO4 [M + H]+ = 442.12606, observed for [M + H]+ = 442.12619.

6-Fluoro-7-methoxy-3-methyl-2-(4-(4-(trifluoromethoxy)phenoxy)phenyl)quinolin-4(1*H*)-one (**1e, HLQ-133**). Using General Procedure A, 4-fluoro-3-methoxyaniline **3e** (1.83 g, 0.013 mol), **12b** (5.00 g, 0.013 mole, 1.0 eq), and a catalytic amount of *p*-TsOH•H_2O (about 0.23 g) were heated in benzene (70 ml) for seven days. The crude Schiff base **15e**, a brown oil, was taken up in hot Dowtherm A (8 ml, then 7 ml to rinse) and added to boiling Dowtherm A (100 ml) over 5 minutes, heating for a total of 15 minutes. The cooled reaction mixture was stirred for 5 minutes with hexanes (300 ml). After settling, the supernatant was decanted from the resulting shaggy, tan solid, which was swirled with ethyl acetate (100 ml) and recovered by vacuum filtration, rinsing with ethyl acetate (100 ml) followed by acetone (150 ml). The crude product (an off-white solid, 1.51 g) was recrystallized from DMF (8 ml), affording the desired product **1e** (**HLQ-133**) as straw-colored needles (0.92 g, yield 15% over two steps from 4-fluoro-3-methoxyaniline), ^{1}H-NMR (400 MHz; DMSO-d6): δ 11.54 (s, 1H), 7.74 (d, J_F = 11.9 Hz, 1H), 7.62-7.58 (m, 2H), 7.48-7.45 (m, 2H), 7.25-7.21 (m, 5H), 3.91 (s, 3H), 1.91 (s, 3H). ^{19}F-NMR (376 MHz; DMSO): δ -57.1 (s, 3F), -139.3 (dd, 1H, J_H = 11.82, 7.59). HRMS calculated for C24H17F4NO4 [M + H]+ = 460.11664, observed for [M + H]+ = 460.11681.

6-Fluoro-3-methyl-2-(4-(4-(trifluoromethoxy)phenoxy)phenyl)quinolin-4(1*H*)-one (1f, HLQ-142). Following General Procedure A, the Schiff base **15f** was obtained by refluxing **12b** (11.46 g, 30 mmol, 1.0 eq), 4-fluoroaniline **3f** (3.50 g, 31.5 mmol, 1.05 eq) and *p*-TsOH·H_2O (570 mg, 3.0 mmol, 0.1 eq) in benzene (50 ml) for 72 hours. The resulting black oil (**15f**) was added over 2 minutes to 60 ml Dowtherm A, heating a total of 7 minutes. The cooled

reaction mixture was stirred briefly with hexanes (350 ml) followed by filtration and washing with hexanes (4 x 50 ml). The resulting 7.73 g of crude product required further purification. Neither crystallization from ethyl acetate and DMF nor from ethyl acetate alone produced a pure sample. Hence, the pivalate was prepared from a portion of the crude **1f** (3.20 g, i.e. 41% of the crude material) in the same manner as described for **1i** (**HLQ-164**). The crude pivalate was then suspended in 10 ml of methylene chloride, warmed and filtered, and the intensely yellow solution was purified by automated flash chromatography on silica with a gradient of 5% to 35% ethyl acetate in hexanes. Pure material was collected (1.25 g). GC-MS: M^+ = 513 (12%), 57 (100%), ^{1}H-NMR (400 MHz; $CDCl_3$): δ 8.13 (ddd, *J* = 9.2, 5.3, 0.3 Hz, 1H), 7.61-7.57 (m, 2H), 7.46 (ddd, *J* = 9.2, 8.3, 2.8 Hz, 1H), 7.31 (dd, *J* = 9.1, 2.6 Hz, 1H), 7.23-7.21 (m, 2H), 7.15-7.11 (m, 2H), 7.10-7.06 (m, 2H), 2.27 (s, 3H), 1.54 (s, 9H); ^{19}F-NMR (376 MHz; CDCl3): δ -58.2, -111.9. To obtain pure **1f** (**HLQ-142**), a portion of the pivalate (0.60 g, 1.17 mmol, i.e. 47% of the total amount of purified pivalate) was heated at 70°C for 20 min in a solution of 0.40 g NaOH in 7.5 ml of methanol; TLC showed the complete consumption of the pivalate. The solution was poured into 15 ml of water with stirring and the precipitate was filtered off, washed with water (3 x 5 ml.), vacuum and air-dried. The product **1f** (**HLQ-142**) was obtained as a pale beige powder (0.49 g, 98% of theory from the pivalate, representing a 20% yield over several steps from the original starting material **12b** after accounting for use of only 41% and 47% of the material in the pivalate formation and saponification steps, respectively), ^{1}H-NMR (400 MHz; DMSO-d_6): δ 11.75 (s, 1H), 7.76 (dd, *J* = 9.5, 3.0 Hz, 1H), 7.69 (dd, *J* = 9.1, 4.7 Hz, 1H), 7.64-7.61 (m, 2H), 7.55 (ddd, *J* = 9.1, 8.3, 3.0 Hz, 1H), 7.49-7.46 (m, 2H), 7.26-7.20 (m, 4H), 1.93 (s, 3H); ^{19}F-NMR (376 MHz; DMSO): δ -57.1 (s, 3F), -118.6 (m, 1F). HRMS calculated for C23H15F4NO3 [M + H]+ = 430.10608, observed for [M + H]+ = 430.10616.

6-Chloro-3-methyl-2-(4-(4-(trifluoromethoxy)phenoxy)phenyl)quinolin-4(1*H*)-one (1g, HLQ-141). Following General Procedure A, the Schiff base **15g** was obtained by refluxing **12b** (11.46 g, 30 mmol, 1.0 eq), 4-chloroaniline **3g** (4.0 g, 31.5 mmol, 1.05 eq), and *p*-TsOH·H_2O (570 mg, 3.0 mmol, 0.1 eq) in benzene (50 ml) for 72 hours. The resulting black oil **15g** (14.6 g) was added over 2 minutes to 75 ml boiling Dowtherm A, followed by 5 minutes further heating. After cooling, the reaction mixture was stirred for 30 minutes with hexanes (300 ml). The solid was filtered off, washed with ethyl acetate (3 x 20 ml) and dried. The crude product **1g** (6.33 g) was obtained. It was heated with 30 ml of ethyl acetate, and at the boiling point DMF was added dropwise (~1.5 ml) until a clear solution was obtained. A mass of crystals formed overnight at room temperature. The crystals were filtered off, washed with several small volumes of ethyl acetate and dried. The product was obtained as a soft, pale yellow solid **1g** (**HLQ-141**, 1.80 g, 14% yield), ^{1}H-NMR (400 MHz; DMSO-d_6): δ 11.77 (s, 1H), 8.05 (d, *J* = 2.0 Hz, 1H), 7.68-7.65 (m, 2H), 7.64-7.60 (m, 2H), 7.48-7.45 (m, 2H), 7.24-7.21 (m, 4H), 1.92 (s, 3H); ^{19}F-NMR (376 MHz; DMSO): δ - 57.1(s). HRMS calculated for C23H15ClF3NO3 [M + H]+ = 446.07653, observed for [M + H]+ = 446.07691.

Ethyl 3-((4-bromophenyl)amino)-2-methyl-3-(4-(4-(trifluoromethoxy)phenoxy)phenyl)acrylate (1h, HLQ-143): Following General Procedure A, the Schiff base **15h** was obtained by refluxing **12b** (11.46 g, 30 mmol, 1.0 eq), 4-bromoaniline **3h** (5.42 g, 31.5 mmol, 1.05 eq), and *p*-TsOH·H_2O (570 mg, 3.0 mmol, 0.1 eq) in benzene (50 ml) for

72 hours. The resulting black oil (**15h**, 17.0 g) was taken up in 5 ml hot Dowtherm A and added to 75 ml boiling Dowtherm A over 2 minutes, followed by 3 minutes further heating. Darkening occurred rapidly and soon, grey fumes appeared. The cooled reaction was stirred with 300 ml of hexanes, and the supernatant was decanted. The sticky residue was stirred with 50 ml of ethyl acetate and again diluted with 300 ml of hexanes, producing a flocculent precipitate that was filtered off and dried for 3 days under vacuum, leaving 5.6 g of a tarry residue. This material was converted to the pivalate for purification by chromatography, using the same method as described for **1i** (**HLQ-164**). The resulting black mass was subjected to automated flash chromatography using a gradient of 5% to 35% ethyl acetate in hexanes. Obtained were 1.77 g of yellowish crystals, which were re-crystallized once from 9:1 hexanes/ethyl acetate, yielding 0.77 g of white, shiny crystals (the pure pivalate), ^{1}H-NMR (400 MHz; $CDCl_3$): δ 7.99 (dd, *J* = 9.0, 0.3 Hz, 1H), 7.86 (d, *J* = 2.0 Hz, 1H), 7.75 (dd, *J* = 9.0, 2.2 Hz, 1H), 7.62-7.58 (m, 2H), 7.23-7.21 (m, 2H), 7.15-7.11 (m, 2H), 7.10-7.06 (m, 2H), 2.27 (s, 3H), 1.55 (s, 9H). A portion of the pivalate (0.65 g, 0.0011 mole, i.e. 84% of the total amount of obtained pivalate) was heated for 10 minutes at 50°C in 7.5 ml of methanol and 0.50 g of 50% NaOH. TLC showed complete absence of the pivalate. After cooling, 25 ml of water was added and the bright white precipitate was vacuum filtered, washed with water (4 x 5 ml), and dried. The desired product **1h** (**HLQ-143**) was obtained as a white powder (0.53 g, 96% from the pivalate, representing an overall yield of 4.3% over several steps from **12b** after correcting for the use of only 84% of pivalate in the saponification reaction), ^{1}H-NMR (400 MHz; DMSO-d6): δ 11.77 (s, 1H), 8.20 (dd, *J* = 2.4, 0.3 Hz, 1H), 7.77 (dd, *J* = 8.9, 2.4 Hz, 1H), 7.63-7.60 (m, 2H), 7.58 (dd, *J* = 8.9, 0.3 Hz, 1H), 7.48-7.45 (m, 2H), 7.25-7.21 (m, 4H), 1.92 (s, 3H); ^{19}F-NMR (376 MHz; DMSO): δ -57.1. Note: High heating during the Conrad Limpach cyclization likely resulted in extensive loss of bromine and replacement by hydrogen, determined by observation of the pivalate of this debrominated side product during GC-MS of the pivalate of the crude cyclization product. HRMS calculated for C23H15BrF3NO3 [M + H]+ = 490.02601, observed for [M + H]+ = 490.02645.

7-Chloro-3-methyl-2-(4-(4-(trifluoromethoxy)phenoxy)phenyl)quinolin-4(1*H*)-one (1i, HLQ-164). Using General Procedure A, *meta*-chloroaniline **3i** (4.01 g, 0.031 mol), **12b** (12.00 g, 0.031 mole, 1.0 eq) and a catalytic amount of *p*-TsOH•H_2O (about 0.59 g) were heated in benzene, 80 ml, for six days. The crude Schiff base **15i**, an inhomogeneous golden-brown oil, was taken up in hot Dowtherm A (10 ml, then 10 ml to rinse) and added to boiling Dowtherm A (100 ml) over the course of 5 minutes, heating for a total of 15 minutes. The cooled reaction mixture was stirred 2 hours with hexanes (300 ml). After standing 12 hours, a yellowish tan solid was recovered by vacuum filtration, rinsing with ethyl acetate (200 ml) followed by acetone (2 x 5 ml). NMR showed that this crude product (a very pale yellow powder) contained both the 7- and 5-chloro regioisomers in an approximate ratio of 1.0:0.15 by mole. To allow chromatographic separation, this mixture was converted to a pivalate ester: sodium hydride (1.05 g of a 60% w/w dispersion in paraffin, thus 0.63 g, 0.026 mole, 2.5 eq of NaH) was deparaffinated by rinsing with hexanes (3 x 5 ml) and taken up in anhydrous tetrahydrofuran (80 ml). The regioisomeric mixture from above (4.65 g, 0.010 mole including both regioisomers) was added in portions over 2 minutes, and the reaction was then stirred at 60°C (sealed with a needle-vented septum) for 40 minutes. After

removing from the heat, pivaloyl chloride (2.0 eq, 2.56 ml, 0.021 mole) was added over 1 minute, followed by stirring at room temperature for 26 hours. The reaction mixture was poured cautiously into ice water (100 ml) while stirring, then partially evaporated under reduced pressure with warming to remove THF. The resulting biphasic mixture was extracted with ethyl acetate (40 ml, then 2 x 30 ml), and the combined organic layers were rinsed with brine (20 ml), dried ($MgSO_4$), and evaporated under reduced pressure with warming. The residue was separated by automated flash chromatography on silica, eluting with a gradient of 100:0 to 90:10 v/v hexanes/ethyl acetate to obtain the purified 7-chloro regioisomer (7-chloro-3-methyl-2-(4-(4-(trifluoromethoxy)phenoxy)phenyl)quinolin-4-yl pivalate, the pivalate ester of **1i**, R_f = 0.51, 93:7 v/v hexanes/ethyl acetate) as a colorless oil (3.66 g, 22% over several steps from the starting aniline), ^{1}H-NMR (400 MHz; $CDCl_3$): δ 8.12 (dd, *J* = 2.0, 0.5 Hz, 1H), 7.66 (dd, *J* = 8.9, 0.4 Hz, 1H), 7.65-7.62 (m, 2H), 7.49 (dd, *J* = 8.9, 2.0 Hz, 1H), 7.48-7.45 (m, 2H), 1.53 (d, *J* = 4.5 Hz, 9H). To obtain **1i**, this oil (3.66 g, 0.0069 mole) was dissolved in 80 ml absolute ethanol by warming. Water (5 ml) and *p*-TsOH•H_2O (3.0 eq, 0.021 mole, 3.94 g) were added, followed by heating at reflux for 19 hours, then overnight standing at room temperature. The crystals that formed in the reaction mixture were recovered by vacuum filtration, rinsing with 3 x 5 ml ethanol, then 1 ml acetone (Product A, 2.02 g cream crystals; the desired product **1i**). The filtrate was partially evaporated under reduced pressure with warming, then poured into water (100 ml) with stirring. After 10 minutes, the resulting white precipitate was recovered by vacuum filtration, rinsing with excess water followed by 2 x 9 ml acetone (Product B, 0.67 g of a white powder, also the desired product **1i**; combined product **1i** (**HLQ-164**) 2.69 g, 87% from the pivalate, 19% over several steps from the starting aniline). ^{1}H-NMR (400 MHz; DMSO-d_6): δ 11.64 (s, 1H), 8.12 (d, *J* = 8.7 Hz, 1H), 7.64-7.60 (m, 3H), 7.48-7.46 (m, 2H), 7.32 (dd, *J* = 8.7, 2.0 Hz, 1H), 7.26-7.20 (m, 4H), 1.91 (s, 3H), ^{19}F-NMR (376 MHz; DMSO): δ -57.1). HRMS calculated for C23H15ClF3NO3 [M + H]+ = 446.07653, observed for [M + H]+ = 446.07688.

6,8-Difluoro-3-methyl-2-(4-(4-(trifluoromethoxy)phenoxy)phenyl)quinolin-4(1*H*)-one (1j, HLQ-174): Using General Procedure A, 3,5-difluoroaniline **3j** (4.06 g, 31.5 mmol, 1.05 eq), **12b** (11.46g, 30 mmol, 1eq), and *p*-TsOH•H_2O (570 mg, 3.0 mmol, 0.1 eq) were heated in benzene (50 ml) for 3 days. The crude Schiff base **15j** (14.8 g) was taken up in hot Dowtherm A (5 ml) and added to boiling Dowtherm A (80 ml) over 5 minutes, heating for a total of 12 minutes. After cooling to 50ºC, hexanes was added to a volume of 400 ml, followed by 5 minutes stirring. The precipitate was filtered off, washing with hexanes (3 x 25 ml), leaving 6.63 g of a beige powder. This powder was crystallized by heating in 40 ml of ethyl acetate to which was added DMF dropwise at the boiling point till a nearly clear solution was obtained, which was then filtered while hot and set aside for crystallization. After 3 hours, the product (3.20 g) was filtered off. An additional 1.32 g were obtained from the filtrate by another re-crystallization, in all 4.52 g (35% yield) of pure product **1j** (**HLQ-174**). ^{1}H-NMR (400 MHz; DMSO-d_6): δ 11.73 (s, 1H), 7.73-7.67 (m, 1H), 7.66-7.63 (m, 1H), 7.59-7.56 (m, 2H), 7.48-7.45 (m, 2H), 7.23-7.18 (m, 4H), 1.89 (s, 3H), 19-F NMR (376 MHz; DMSO): δ -57.1 (3F, s), -116.1 (1F, m), -122.9 (1F, m). HRMS calculated for C23H14F5NO3 [M + H]+ = 448.09666, observed for [M + H]+ = 448.09698.

7-Chloro-6-methoxy-3-methyl-2-(4-(4-(trifluoromethoxy)phenoxy)phenyl)quinolin-4(1*H*)-one (1k, HLQ-175). Using General Procedure A, 3-chloro-4-methoxyaniline **3k** (9.96 g, 63.5 mmol, 1.05 eq), **12b** (22.92 g, 60 mmol), and *p*-TsOH•H_2O (1.14 g, 6.0 mmol, 0.1 eq) were heated in benzene (50 ml), for 3 days. The crude Schiff base **15k** (33.4 g) was taken up in hot Dowtherm A (5 ml) and added to boiling Dowtherm A (135 ml) over 4 minutes, heating for a total of 10 minutes. After cooling, hexanes was added to a total volume of 550 ml, followed by 30 minutes of stirring. The precipitate was filtered off, washing with hexanes (25 ml). The resulting solid was stirred 25 minutes with ethyl acetate (100 ml), diluted with hexanes (100 ml), and again filtered, washing with hexanes (100 ml), to afford a yellow solid (15.3 g). GC-MS analysis of a pivalate derivative made from this crude product showed a mixture of the two positional isomers (5-chloro-6-methoxy and 7-chloro-6-methoxy). The crude product was therefore converted to the pivalate for purification by chromatography, using the same method as described for **1i** (**HLQ-164**). Reaction with NaH in THF was accompanied by the formation of a voluminous mass of firm foam that collapsed only slowly. Of the resulting crude pivalate mixture (a total of 9.40 g), 67% (6.30 g) was subjected to automated flash chromatography using a gradient of 5% to 31% ethyl acetate in hexanes. The pure desired pivalate (7-chloro-6-methoxy-3-methyl-2-(4-(4-(trifluoromethoxy)phenoxy)phenyl)quinolin-4-yl pivalate) was obtained as a glass (1.49 g). ^{1}H-NMR (400 MHz; $CDCl_3$): δ 8.17 (s, 1H), 7.60-7.56 (m, 2H), 7.23-7.21 (m, 2H), 7.14-7.10 (m, 2H), 7.09-7.06 (m, 2H), 7.00 (s, 1H), 3.99 (s, 3H), 2.25 (s, 3H), 1.55 (s, 9H); ^{19}F-NMR (376 MHz; CDCl3): δ -58.2; GC-MS: single band, m^+= 85 (100%), 559 (14 %), 561 (5 %) (molecular ion corresponding to ^{35}Cl, ^{37}Cl= 3:1). To obtain pure **1k** (**HLQ-175**), a portion of this pivalate (1.25 g, 0.0022 mole, i.e. 84% of the total amount of obtained pivalate) was heated for 30 minutes at a boil in 75 ml of methanol with 2.0 g of 50% NaOH. TLC showed complete conversion. The solution was poured into 400 ml of water with vigorous stirring. After 10 minutes, the solid was collected by filtration and washed with water (3 x 25 ml) to afford a dull white solid (1.05 g, 99% from the pivalate, representing an overall yield of 6.5% over several steps from **12b** after correcting for the use of only 67% of crude product in the pivalate-forming reaction and 84% of pivalate in the saponification reaction). ^{1}H-NMR (400 MHz; DMSO-d_6): δ 11.59 (s, 1H), 7.71 (s, 1H), 7.64 (s, 1H), 7.62-7.60 (m, 2H), 7.48-7.46 (m, 2H), 7.25-7.20 (m, 4H), 3.94 (s, 3H), 1.92 (s, 3H); ^{19}F-NMR (376 MHz; DMSO): δ -57.1. HRMS calculated for C24H17ClF3NO4 [M + H]+ = 476.08709, observed for [M + H]+ = 476.08726.

6-Chloro-7-methoxy-3-methyl-2-(4'-(trifluoromethoxy)-[1,1'-biphenyl]-4-yl)quinolin-4(1*H*)-one (2a, HLQ-105). Using General Procedure E, a mixture of **28a** (325 mg, 0.68 mmol, 1.0 eq), anhydrous potassium acetate (666 mg, 6.8 mmol, 10 eq) and glacial acetic acid (5 ml) was heated at 120°C for 18 hours. After cooling to room temperature, the reaction mixture was poured into ice water (30 ml). The resulting precipitate was filtered and washed with water (3 x 20 ml), acetone (3 x 10 ml), DCM (3 x 10 ml), and hexanes (3 x 10 ml) and air-dried to give pure **2a** (**HLQ-105**) as a white solid (205 mg, 65% yield, mp 303.4-304.5 ^{o}C (dec)). ^{1}H-NMR (400 MHz; DMSO-d_6): δ 11.63 (s, 1H), 8.06 (s, 1H), 7.93-7.90 (m, 4H), 7.68 (d, *J* = 8.1 Hz, 2H), 7.52 (d, *J* = 8.0 Hz, 2H), 7.20 (s, 1H), 3.93 (s, 3H), 1.93 (s, 3H). ^{13}C-NMR (101 MHz; DMSO-d_6): δ 176.4, 157.7, 149.4, 148.2, 140.99, 140.81, 139.7, 135.5, 130.8,

129.9, 128.1, 126.95, 126.95, 122.7, 119.25, 119.23, 118.8, 115.6, 100.9, 57.4, 13.3. HRMS calculated for C24H17ClF3NO3 [M + H]+ = 460.09218, observed for [M + H]+ = 460.09250.

5,7-Difluoro-3-methyl-2-(4'-(trifluoromethoxy)-[1,1'-biphenyl]-4-yl)quinolin-4(1*H*)-one (2b, HLQ-128). Using General Procedure E, a mixture of **28b** (0.77 g, 0.0017 mole), anhydrous potassium acetate (1.67 g, 0.017 mole, 10 eq), and glacial acetic acid (10 ml) was heated at 115°C in a loosely capped reaction vial for 22 hours. The hot reaction mixture was poured into water (110 ml). The resulting precipitate was collected by vacuum filtration, rinsing with excess water followed by acetone (3 x 2 ml) to afford a white powder, the desired product **2b** (**HLQ-128**, 0.67 g, 91% yield). ^{1}H-NMR (400 MHz; DMSO-d$_6$): δ 11.72 (s, 1H), 7.92-7.89 (m, 4H), 7.68-7.66 (m, 2H), 7.55-7.50 (m, 2H), 7.16 (ddd, J_F = 10.0, 1.2 Hz, J_H = 2.2 Hz, 1H), 7.04 (ddd, J_F = 12.0, 9.6 Hz, J_H = 2.4 Hz, 1H), 1.88 (s, 3H); ^{19}F-NMR (376 MHz; DMSO): δ -56.7 (s, 3F), -105.5 (m, 1F), -108.6 (m, 1F). HRMS calculated for C23H14F5NO2 [M + H]+ = 432.10174, observed for [M + H]+ = 432.10187.

3-Methyl-2-(4'-(trifluoromethoxy)-[1,1'-biphenyl]-4-yl)quinolin-4(1*H*)-one (2c, HLQ-126). Using General Procedure E, a mixture of **28c** (1.03 g, 0.0025 mole), anhydrous potassium acetate (1.77 g, 0.018 mole, 7.2 eq), and glacial acetic acid (10 ml) was heated at 120°C for 22 hours. After cooling slightly, the reaction mixture was poured into water (100 ml). The resulting precipitate was collected by vacuum filtration, rinsing with excess water followed by acetone (3 x 2 ml) to afford a white powder, the desired product **2c** (**HLQ-126**, 0.80 g, 81% yield). ^{1}H-NMR (400 MHz; DMSO-d$_6$): δ 11.64 (s, 1H), 8.15-8.12 (m, 1H), 7.92-7.90 (m, 4H), 7.70-7.68 (m, 2H), 7.63-7.62 (m, 2H), 7.55-7.50 (m, 2H), 7.31 (ddd, J = 8.1, 5.0, 3.1 Hz, 1H), 1.95 (s, 3H), ^{19}F-NMR (376 MHz; DMSO): δ -56.7). HRMS calculated for C23H16F3NO2 [M + H]+ = 396.12058, observed for [M + H]+ = 396.12062.

7-Methoxy-3-methyl-2-(4’-(trifluoromethoxy)-[1,1’-biphenyl]-4-yl)12uinoline-4(1*H*)-one (2d, HLQ-132). Using General Procedure E, a mixture of **28d** (1.08 g, 0.0024 mole), anhydrous potassium acetate (2.39 g, 0.024 mole, 10 eq), and glacial acetic acid (12.5 ml) was heated at 120°C for 22 hours. The hot reaction mixture was poured into water (115 ml). The resulting precipitate was collected by vacuum filtration, rinsing with excess water followed by acetone (10 ml, then 2 x 2 ml) to afford an off-white powder, the desired product **2d** (**HLQ-132**, 0.93 g, 91% yield). ^{1}H-NMR (400 MHz; DMSO-d$_6$): δ 11.44 (s, 1H), 8.03 (d, J = 9.0 Hz, 1H), 7.92-7.88 (m, 4H), 7.68-7.66 (m, 2H), 7.52-7.50 (m, 2H), 7.03 (d, J = 2.4 Hz, 1H), 6.91 (dd, J = 9.0, 2.4 Hz, 1H), 3.83 (s, 3H), 1.92 (s, 3H), ^{19}F-NMR (376 MHz; DMSO): δ -56.7. HRMS calculated for C24H18F3NO3 [M + H]+ = 426.13115, observed for [M + H]+ = 426.13139.

6-Fluoro-7-methoxy-3-methyl-2-(4'-(trifluoromethoxy)-[1,1'-biphenyl]-4-yl)quinolin-4(1*H*)-one (2e, HLQ-147). Using General Procedure E, a mixture of **28e** (0.80 g, 0.0017 mole), anhydrous potassium acetate (1.67 g, 0.017 mole, 10 eq), and glacial acetic acid (10 ml) was heated at 115°C for 22 hours. The hot reaction mixture was poured into water (100 ml) followed by 20 minutes stirring. The resulting precipitate was collected by vacuum filtration, rinsing with excess water followed by acetone (3 x 2 ml) to afford a cream powder, the desired product **2e** (**HLQ-**

147, 0.63 g, 82% yield). ^{1}H-NMR (400 MHz; DMSO-d_6): δ 11.60 (s, 1H), 7.93-7.89 (m, 4H), 7.75 (d, J_F = 11.9 Hz, 1H), 7.69-7.66 (m, 2H), 7.54-7.50 (m, 2H), 7.54-7.50 (m, 2H), 7.22 (d, J_F = 7.5 Hz, 1H), 3.91 (s, 3H), 1.93 (s, 3H). ^{19}F-NMR (376 MHz; DMSO): δ -56.7 (s, 3F), -139.3 (dd, J_H = 11.8, 7.4 Hz). HRMS calculated for C24H17F4NO3 [M + H]+ = 444.12173, observed for [M + H]+ = 444.12190.

7-Chloro-3-methyl-2-(4'-(trifluoromethoxy)-[1,1'-biphenyl]-4-yl)quinolin-4(1*H*)-one (2i, HLQ-165). Using General Procedure E, a mixture of **28i** (0.33 g containing side product, <0.00074 mole of the desired starting material), anhydrous potassium acetate (0.72 g, 0.0074 mole, 10 eq), and glacial acetic acid (10 ml) was heated at 115°C in a loosely capped reaction vial for 1 day. The hot reaction mixture was poured into water (100 ml) and the resulting precipitate was collected by vacuum filtration, rinsing with excess water followed by acetone (2 x 1.5 ml) to afford a white powder, the desired product (0.11 g, 18% yield over two steps from **23i**). ^{1}H-NMR (400 MHz; DMSO-d_6): δ 11.70 (s, 1H), 8.14 (d, J = 8.7 Hz, 1H), 7.93-7.90 (m, 4H), 7.73-7.68 (m, 2H), 7.64 (d, J = 1.8 Hz, 1H), 7.54-7.50 (m, 2H), 7.33 (dd, J = 8.7, 1.9 Hz, 1H), 1.94 (s, 3H); ^{19}F-NMR (376 MHz; DMSO): δ -56.7). HRMS calculated for C23H15ClF3NO2 [M + H]+ = 430.08161, observed for [M + H]+ = 430.08190.

6-Chloro-2-heptyl-7-methoxyquinolin-4(1H)-one (6, HLQ-120). According to General Procedure A[3], **4** (11.7 g crude, 54 mmol, 1.1 eq), 4-chloro-3-methoxyaniline **3** (7.7 g, 49 mmol), *p*TosOH·H_2O (a catalytic amount, 50 mg) and 150 ml of benzene were heated at reflux for 22 hours. The crude Schiff base **5** was added to 80 ml of boiling Dowtherm A over 2 minutes, boiling being continued for another 10 minutes. The cooled reaction mixture was stirred with hexanes (200 ml) for 25 minutes, and the precipitate was filtered off and washed with ethyl acetate (2 x 20 ml) and acetone (2 x 20 ml). The product **6** (**HLQ-120**) was obtained as a grey powder (5.93 g, 36%), ^{1}H-NMR (400 MHz; DMSO-d_6): δ 11.45 (s, 1H), 7.94 (s, 1H), 7.07 (s, 1H), 5.87 (d, J = 1.6 Hz, 1H), 3.94 (s, 3H), 2.55 (t, J = 7.6 Hz, 2H), 1.65 (t, J = 5.8 Hz, 2H), 1.31-1.26 (m, 8H), 0.86 (t, J = 6.9 Hz, 3H). HRMS calculated for C17H22ClNO2 [M + H]+ = 308.14118, observed for [M + H]+ = 308.14138.

6-Chloro-2-heptyl-7-methoxy-3-methylquinolin-4(1H)-one (9a, HLQ-119). Following General Procedure A, **7** (5.00 g, 23.4 mmol), 4-chloro-3-methoxyaniline **3a** (3.65 g, 23.2 mmol), *p*-TsOH·H_2O (100 mg), and benzene (150 ml) were heated for 19 days (for this reaction, the formation of the Schiff base appeared to be very slow). The crude Schiff base **8a** was added to 80 ml boiling Dowtherm A over 4 minutes, heating for a total of 9 minutes. The cooled reaction mixture was stirred with hexanes (400 ml) for 20 minutes. After settling, the supernatant was decanted and the residue stirred with ethyl acetate (50 ml). The now-solid precipitate was filtered, washed with acetone (4x5 ml), and air-dried to give the crude product **9a** (**HLQ-119**) (2.60 g). This material was re-crystallized twice from about 15 ml of DMF to produce the pure product **9a** (**HLQ-119**) (1.9 g, 26%). ^{1}H-NMR (400 MHz; DMSO-d_6): δ 11.32 (s, 1H), 7.96 (s, 1H), 7.04 (s, 1H), 3.93 (s, 3H), 2.64 (t, J = 7.9 Hz, 2H), 1.96 (s, 3H), 1.65-1.57 (m, 2H), 1.37-1.25 (m, 8H), 0.86 (t, J = 7.0 Hz, 4H).

5,7-Difluoro-2-heptyl-3-methylquinolin-4(1H)-one (9b, HLQ-118). Following General Procedure A, ethyl 2-methyl-3-ketodecanoate **7** (12.0 g, crude, 52.6 mmol), 5.7-difluoroaniline **3b** (6.8 g, 52.7 mmol), *p*-TsOH·H2O (300mg) and 80 ml of benzene were heated to reflux for 3 days with constant water removal. After removal of the solvent the residue (Schiff base **8b**) was added to 80 ml of boiling Dowtherm A in about 4 minutes, boiling being continued for another 5 minutes. After cooling with stirring, ethyl acetate (200 ml) was added, and after 15 minutes of stirring the precipitate was filtered off, washed with ethyl acetate (3x30 ml), and dried. The product was obtained as a whitish powder, 4.77 g (34.7 %), pure by NMR spectroscopy (1H-NMR (400 MHz; DMSO-d6): δ 11.41 (s, 1H), 7.04 (ddd, *J* = 10.0, 2.4, 1.4 Hz, 1H), 6.96 (ddd, *J* = 12.0, 9.7, 2.4 Hz, 1H), 2.62 (t, *J* = 7.9 Hz, 2H), 1.62-1.58 (m, 2H), 1.37-1.26 (m, 8H), 0.86 (t, *J* = 6.9 Hz, 3H), ^{19}F-NMR (376 MHz; DMSO): δ -106.2, -108.8). HRMS calculated for C17H21F2NO [M + H]+ = 294.16639, observed for [M + H]+ = 294.16651.

1-(4-(4-(Trifluoromethoxy)phenoxy)phenyl)ethan-1-one (11a). *p*-Fluoroacetophenone **10a** (17.0 g, 123 mmol), *p*-trifluoromethoxyphenol (23.6 g, 133 mmol, 1.08 eq), anhydrous K_2CO_3 (21 g, 151.9 mmol, 1.2 eq), and 75 ml DMF were heated with stirring at 125-130°C for 19 h. When cooled, the solids were removed by filtration and rinsed with several portions of ethyl acetate, and the filtrate was concentrated (rotary evaporator, 75°C, 0.02 atm). The dark orange residue was stirred with 300 ml of water containing 30 g of NaCl and 200 ml of hexanes After separating the layers, the aqueous phase was once more extracted with 100 ml of hexanes. The combined hexanes extracts were freed of the solvent (rotary evaporator), leaving 36.6 g of the crude product as a brown, clear, oily liquid showing only a single spot on TLC and a single band (M^+= 296) in GC-MS. This crude product was used without further purification. Because in the NMR spectrum of the crude material several impurities were seen, a sample was chromatographed for analysis to confirm structure, ^{1}H-NMR (400 MHz; $CDCl_3$): δ 7.98-7.95 (m, 2H), 7.26-7.23 (m, overlaps solvent residual signal, estimated ~2H), 7.10-7.06 (m, 2H), 7.03-7.00 (m, 2H), 2.58 (s, 3H); ^{19}F-NMR (376 MHz; CDCl3): δ -58.2.

1-(4-(4-(Trifluoromethoxy)phenoxy)phenyl)propan-1-one (11b). The synthesis of the diaryl ether **11b** has been previously reported using a different synthetic route involving a copper acetate catalyst[5]. Herein, we describe a simplified synthesis of **11b**. A stirring mixture of 1-(4-fluorophenyl)propan-1-one (152.2 g, 1.0 mol, 1.00 eq), 4-(trifluoromethoxy)phenol (186.9 g, 1.05 mol, 1.05 eq), and potassium carbonate (208.5 g, 1.5 mol, 1.2 eq) in DMF (750 ml) were heated at 140°C for 72 hours, when no more starting material was detected by GC-MS. After cooling to room temperature, the reaction mixture was filtered through Celite and the filtrate was concentrated under reduced pressure to give a black oil (327 g), which solidified upon standing at room temperature. NMR of the crude material indicated that the product was 90-95% pure. The product (265 g) was purified by flash chromatography using an isocratic eluent of hexanes/ethyl acetate (8:2) to give a brown solid (235 g). This material was crystallized in hexanes to give pure **11b** (145 g) as a white solid. A second crop from the mother liquor gave an additional 22 g for a total of 167 g of pure **11b** as a white solid (63% yield). ^{1}H-NMR (400 MHz; $CDCl_3$): δ 8.03-7.99

(m, 2H), 7.28-7.26 (m, 2H), 7.13-7.09 (m, 2H), 7.06-7.02 (m, 2H), 4.35 (q, *J* = 7.1 Hz, 1H), 4.20-4.15 (m, 2H), 1.51 (d, *J* = 7.1 Hz, 3H), 1.21 (t, *J* = 7.1 Hz, 3H).

Ethyl 3-oxo-3-(4-(4-(trifluoromethoxy)phenoxy)phenyl)propanoate (12a). 4-(4-Trifluoromethoxyphenoxy)-acetophenone **11a** (15.08 g, 51.0 mmol) was added in small portions to a stirred slurry of NaH (4.6 g of a 60 % dispersion by weight in paraffin, deparaffinated by washing with hexanes (2 x 30 ml), 2.2. eq) in 100 ml of anhydrous THF. When the reaction had subsided, diethylcarbonate (12.1 g, 102.5 mmol) was added and the mixture was heated at reflux for 3 hours. TLC indicated that no starting material was left at that time. The mixture was then stored at 5°C overnight. The next day, hydrochloric acid (15 %) was added with stirring to pH <7 (color lightens), and a white, sticky mass separated. The supernatant was decanted, and the sticky residue was stirred with two 100 ml portions of ethyl acetate, followed by decanting. The three supernatants were combined, and the solvent was removed (rotary evaporator, 75°C, 0.02 atm). There remained 18.5 g of a pale brown oil, which very slowly crystallized. This crude product was used without further purification.

Ethyl 2-methyl-3-oxo-3-(4-(4-(trifluoromethoxy)phenoxy)phenyl)propanoate (12b). To a stirred solution of diaryl ether **11b** (6.2 g, 20 mmol, 1.0 eq) in dry THF (200 ml) , a 60% oil suspension of NaH (1.6 g, 2 eq, 40 mmol) was added portion-wise over 5-10 minutes, followed by the addition of ethyl carbonate (9.44 g, 4.0 eq, 80 mmol). The reaction mixture was then heated at 70°C until no ketone starting material remained, as determined by GC-MS and TLC, which took 5 hours. After the reaction was cooled to room temperature, water (20 ml) was added. The organic layer was separated, and the aqueous layer was extracted with ethyl acetate (3 x 20 ml). The combined organic layer was concentrated under reduced pressure to give a yellow, oily solid, which was suspended in ethyl acetate (250 ml) and washed with water (3x30 ml). The organic layer was dried over Mg_2SO_4 and filtered. The filtrate was concentrated to dryness under reduced pressure to give crude **12b** (10.39 g), which was purified by flash chromatography using a gradient of hexane/ethyl acetate to give the pure product **12b** (3.2 g, 42%) as a yellow oil. ^{1}H-NMR (400 MHz; $CDCl_3$): δ 8.03-7.99 (m, 2H), 7.28-7.26 (m, 2H), 7.13-7.09 (m, 2H), 7.06-7.02 (m, 2H), 4.35 (q, J = 7.1 Hz, 1H), 4.20-4.15 (m, 2H), 1.51 (d, J = 7.1 Hz, 3H), 1.21 (t, J = 7.1 Hz, 3H).

6-Chloro-7-methoxy-2-(4-(4-(trifluoromethoxy)phenoxy)phenyl)quinolin-4(1*H*)-one (14, HLQ-101). Following General Procedure A, the Schiff base **13** was obtained by refluxing **12a** (10.0 g, 27.2 mmol, 1.0 eq), 4-chloro-3-methoxyaniline **3a** (4.29 g, 27.2 mmol, 1.0 eq), and *p*-TsOH·H_2O (a catalytic amount, approximately 0.42g) in benzene (50 ml) for 9 days. The residue was diluted in Dowtherm A (5 ml) and added to boiling Dowtherm A (75 ml) over 2 minutes, heating for a total of 9 minutes. After cooling, hexanes was added (to a volume of 500 ml) followed by 10 minutes stirring, then settling. The supernatant was decanted, and the residue was again stirred with hexanes (100 ml), followed by settling and decanting. The residue was stirred with ethyl acetate (50 ml) and the resulting solid was recovered by filtration, rinsing with ethyl acetate (50 ml) and acetone (3 x 10 ml), which provided a gray powder (crude **14** (**HLQ-101**), 5.2 g, 42% yield), ^{1}H-NMR (400 MHz; DMSO-d_6): δ 11.74 (br s, 1H),

8.02 (s, 1H), 7.91-7.88 (m, 2H), 7.48-7.45 (m, 2H), 7.38 (s, 1H), 7.26-7.22 (m, 4H), 6.35 (br s, 1H), 3.97 (s, 3H). ^{19}F-NMR (376 MHz; DMSO): δ -57.1. The product contained a small percentage of impurity that could not be removed by crystallization from DMF. To generate a sample of higher purity for biological testing, a portion of the product was converted to a 4-chloro derivative to allow purification by chromatography: **14** (1.0 g, 0.0021 moles) was heated in $POCl_3$ (3 ml) for 5 minutes. The reaction mixture was poured into water and stirred, followed by filtration. The resulting solid was then digested with DCM (ca. 10 ml), filtered, concentrated and purified by automated flash chromatography using a gradient of 2 to 20% ethyl acetate in hexanes (144 mg, 14% yield of the 4-chloro derivative of **14**, 4,6-dichloro-7-methoxy-2-(4-(4-(trifluoromethoxy)phenoxy)phenyl)quinoline). To obtain pure **14**, following General Procedure E the 4-chloroquinoline was heated with a solution of potassium acetate in glacial acetic acid (obtained by dissolving 100 mg of potassium carbonate in 5 ml of glacial acetic acid) at 125°C overnight. After cooling, water (5 ml) was added, followed by filtration, washing with water (3 x 2 ml), and drying. Obtained were 127 mg of a white solid, pure **14** (91% from the chloride), ^{1}H-NMR (400 MHz; DMSO-d_6): δ 11.67 (br s, 1H), 8.01 (s, 1H), 7.90-7.86 (m, 2H), 7.47-7.45 (m, 2H), 7.36 (s, 1H), 7.26-7.22 (m, 4H), 6.31 (d, *J* = 1.8 Hz, 1H), 3.96 (s, 3H). ^{19}F-NMR (376 MHz; DMSO): δ -57.1. HRMS calculated for C23H15ClF3NO4 [M + H]+ = 462.07144, observed for [M + H]+ = 462.07184.

Ethyl 3-(4-bromophenyl)-2-methyl-3-oxopropanoate (17b). To a stirred solution of 4-bromo propanone **16b** (10.65 g, 150 mmol, 1.0 eq) in dry THF (100 ml), a 60% oil suspension of NaH (4.0 g, 100 mmol, 2.0 eq) was added portion-wise over 5-10 minutes, followed by the addition of ethyl carbonate (23.6 g, 200 mmol, 4.0 eq). The reaction mixture was then heated at 70°C until no ketone starting material remained, as determined by GC-MS and TLC, which took 3 hours. After the reaction was cooled to room temperature, water (20 ml) was added. The organic layer was separated, and the aqueous layer was extracted with ethyl acetate (3 x 20 ml). The combined organic layer was concentrated under reduced pressure to give a yellow, oily solid, which was suspended in ethyl acetate (250 ml) and washed with water (3 x 30 ml). The organic layer was dried over Mg_2SO_4 and filtered. The filtrate was concentrated to dryness under reduced pressure to give crude **17b** (13.55 g), which was purified by flash chromatography using a gradient of hexanes/ethyl acetate to give the pure product **17b** (7.72 g, 54%) as a yellow oil. ^{1}H-NMR (400 MHz; $CDCl_3$): ^{1}H-NMR (400 MHz; $CDCl_3$): δ 7.88-7.85 (m, 2H), 7.66-7.63 (m, 2H), 4.33 (q, *J* = 7.1 Hz, 1H), 4.17 (q, *J* = 7.1 Hz, 2H), 1.51 (d, *J* = 7.1 Hz, 3H), 1.19 (t, *J* = 7.1 Hz, 3H). Note: The crude product can also be used without further purification in the next step (without chromatographic purification).

2-(4-Bromophenyl)-6-chloro-7-methoxyquinolin-4(1H)-one (19, HLQ-107). Following General Procedure A, the Schiff base **18** was obtained from 4-chloro-3-methoxyaniline **3a** (5.8 g, 37.6 mmol, 1.0 eq), **17a** (10.0 g, 36.9 mmol, 1.0 eq), and *p*-TsOH.H_2O (50 mg) by refluxing in benzene (100 ml) for 3 days. The crude Schiff base **18** (a black oil, 3.5 g) was dissolved in 10 ml of Dowtherm A and added to 40 ml of boiling Dowtherm A over 2 minutes followed by an additional 5 minutes heating. The mixture was allowed to cool while stirring was maintained. The solid was filtered, washed with ethyl acetate (15 ml) and acetone (5 ml), and air-dried to give pure **19** (**HLQ-107**, 1.37g, 37%

yield) as a cream-colored solid. ^{1}H-NMR (400 MHz; DMSO-d6): δ 11.73 (s, 1H), 8.02 (s, 1H), 7.79 (s, 4H), 7.35 (s, 1H), 6.34 (s, 1H), 3.97 (s, 3H). HRMS calculated for C16H11BrClNO2 [M]- = 361.95779, observed for [M]- = 361.95917.

2-(4-Bromophenyl)-4,6-dichloro-7-methoxyquinoline (20). Using General Procedure B, **a** stirred solution of **19** (**HLQ-107**, 1.46 g, 4.0 mmol, 1 eq) and $POCl_3$ (10 ml) was refluxed for 1 h. After cooling to room temperature, the precipitate was filtered and washed with hexanes (3 x 20 ml) and air dried to give the HCl salt of **20** (1.88 g) as a yellow solid. This product was then stirred in 10% aqueous NaOH (50 ml) for 12 hours, filtered, washed with water (3 x 20 ml), and air dried to give pure **20** (1.36 g, 89% yield) as a white solid. ^{1}H-NMR (400 MHz; $CDCl_3$): δ 8.26 (s, 1H), 8.04-8.01 (m, 2H), 7.84 (s, 1H), 7.71-7.65 (m, 3H), 4.12 (s, 3H).

2-(4-Bromophenyl)-6-chloro-7-methoxy-3-methylquinolin-4(1*H*)-one (22a, HLQ-108). Using General Procedure A, **17b** (28.5 g, 100 mmol, 1.0 eq), 4-chloro-3-methoxyaniline **3a** (15.8 g, 100 mmol, 1.0 eq), and *p*-TsOH•H_2O (950 mg, 5 mmol, 0.05 eq) were heated in benzene (150 ml) for 3 days. The Schiff base **21a** (32.3 g) was added to 150 ml of boiling Dowtherm A over 4 minutes, heating for a total of 10 minutes. After cooling, the reaction mixture was briefly stirred with 400 ml of hexanes followed by settling and decanting. The residue was stirred with 100 ml ethyl acetate for 30 minutes, and the resulting solid was recovered by vacuum filtration, rinsing with ethyl acetate (50 ml). The crude product (9.60 g) was recrystallized from DMF to afford the desired product **22a** (6.2 g and 1.1 g from the first and second crops respectively). An additional 1.9g of **22a** was obtained by re-filtering the original ethyl acetate filtrate (total yield of **22a** (**HLQ-108**) 9.1 g, 32% yield). ^{1}H-NMR (400 MHz; DMSO-d_6): δ 11.59 (s, 1H), 8.04 (s, 1H), 7.81-7.77 (m, 2H), 7.54-7.51 (m, 2H), 7.15 (s, 1H), 3.92 (s, 3H), 1.86 (s, 3H). HRMS calculated for C17H13BrClNO2 [M + H]+ = 377.98909, observed for [M + H]+ = 377.98939.

2-(4-Bromophenyl)-5,7-difluoro-3-methylquinolin-4(1*H*)-one (22b). Using General Procedure A, **17b** (10.00 g, 0.035 mole), 3,5-difluoroaniline **3b** (0.035 mole, 4.94 g, 1.0 eq), and *p*-TsOH•H_2O (a catalytic amount, about 0.7 g) were heated for 3 days in benzene (75 ml) for three days. The crude Schiff base **21b** (a reddish brown, inhomogeneous oil) was taken up in hot Dowtherm A (5 ml) and added to 100 ml of boiling Dowtherm A over the 6 minutes, heating for a total of 16 minutes. The cooled reaction mixture was stirred with 300 ml of hexanes for 5 minutes. The resulting solid was recovered by vacuum filtration and rinsed with ethyl acetate (250 ml) followed by acetone (3 x 5 ml). The pale orange powder thus obtained (**22b**) was sufficiently pure for use in the following chlorination reaction without recrystallization (6.73 g, 55% yield). ^{1}H-NMR (400 MHz; DMSO-d_6): δ 11.68 (s, 1H), 7.81-7.78 (m, 2H), 7.54-7.51 (m, 2H), 7.12 (ddd, J = 10.0, 2.4, 1.4 Hz, 1H), 7.04 (ddd, J = 12.0, 9.6, 2.4 Hz, 1H), 1.81 (s, 3H), ^{19}F-NMR (376 MHz; DMSO): δ -105.4 (m), -108.6 (m).

2-(4-Bromophenyl)-3-methylquinolin-4(1*H*)-one (22c). Using General Procedure A, ethyl 3-(4-bromophenyl)-2-methyl-3-oxopropanoate (**17b**, 9.00 g, 0.032 mole), aniline **3c** (1.0 eq, 0.032 mole, 2.94 g), and *p*-TsOH•H_2O (a

catalytic amount, about 0.4 g) were heated in benzene (90 ml) for six days. The crude Schiff base **21c**, a brown oil, was added without dilution to 100 ml of boiling Dowtherm A over 8 minutes, heating for a total of 18 minutes. The cooled reaction mixture was stirred with 300 ml of hexanes, and the resulting solid was recovered by vacuum filtration, rinsing with ethyl acetate (excess) followed by acetone (3 x 15 ml). The pale yellow powder thus obtained (4.55 g) was recrystallized from 13 ml of DMF to afford the desired product **22c** as a very pale yellow powder (2.45 g, 24%, yield). ^{1}H-NMR (400 MHz; DMSO-d_6): δ 11.60 (s, 1H), 8.14-8.11 (m, 1H), 7.81-7.77 (m, 2H), 7.65-7.57 (m, 2H), 7.55-7.52 (m, 2H), 7.30 (ddd, *J* = 8.1, 6.5, 1.6 Hz, 1H), 1.88 (s, 3H).

2-(4-Bromophenyl)-7-methoxy-3-methylquinolin-4(1*H*)-one (22d). Using General Procedure A, ethyl 3-(4-bromophenyl)-2-methyl-3-oxopropanoate 17**b**, 10.00 g, 0.035 mole), *meta*-anisidine **3d** (0.035 mole, 4.32 g, 1.0 eq), and *p*-TsOH•H_2O (a catalytic amount, about 0.3 g) were heated at reflux in benzene (80 ml) for seven days. The crude Schiff base **21d** (a brown syrup) was taken up in hot Dowtherm A (12 ml, then 8 ml to rinse) and added to 100 ml of boiling Dowtherm A over 6 minutes, heating for a total of 16 minutes. The cooled reaction mixture was stirred with 300 ml of hexanes for 10 minutes, and the supernatant was decanted from a brown, sticky solid that was then mixed with 150 ml of ethyl acetate. The resulting solid was recovered by vacuum filtration and rinsed with ethyl acetate (300 ml) followed by acetone (3 x 10 ml). The pale yellow powder thus obtained (3.43 g) was recrystallized from 12 ml of DMF to afford the desired product **22d** as a very pale yellow powder (2.31 g, 19% yield). ^{1}H-NMR (400 MHz; DMSO-d_6): δ 11.41 (s, 1H), 8.02 (d, *J* = 9.0 Hz, 1H), 7.79-7.77 (m, 2H), 7.53-7.50 (m, 2H), 6.99 (d, *J* = 2.4 Hz, 1H), 6.91 (dd, *J* = 9.0, 2.4 Hz, 1H), 3.83 (s, 3H), 1.85 (s, 3H).

2-(4-Bromophenyl)-6-fluoro-7-methoxy-3-methylquinolin-4(1*H*)-one (22e). Using General Procedure A, **17b** (10.00 g, 0.035 mole), 4-fluoro-3-methoxyaniline **3e** (0.035 mole, 4.52 g, 1.0 eq), and *p*-TsOH•H_2O (a catalytic amount, about 0.7 g) were heated for 3 days at reflux in benzene (75 ml). The crude Schiff base **21e** (a dark oil) was taken up in hot Dowtherm A (5 ml) and added to 100 ml of boiling Dowtherm A over 8 minutes, heating for a total of 15 minutes. After cooling, the reaction mixture was stirred with 300 ml of hexanes for 5 minutes. The resulting solid was recovered by vacuum filtration and rinsed with ethyl acetate (250 ml) followed by acetone (2 x 5 ml). The sulfur-yellow powder thus obtained (6.73 g) was recrystallized from 50 ml of DMF to afford the desired product **22e** as a beige powder (4.06 g, 32% yield). ^{1}H-NMR (400 MHz; DMSO-d_6): δ 11.56 (s, 1H), 7.80-7.77 (m, 2H), 7.73 (d, J_F = 11.8 Hz, 1H), 7.54-7.50 (m, 2H), 7.18 (d, J_F = 7.4 Hz, 1H), 3.91 (s, 3H), 1.86 (s, 3H), ^{19}F-NMR (376 MHz; DMSO): δ -139.1 (dd, J_H = 11.7, 7.5 Hz).

2-(4-Bromophenyl)-7-chloro-3-methylquinolin-4(1*H*)-one (22i). Using General Procedure A, *meta*-chloroaniline **3i** (6.76 g, 0.053 mole), **17b** (15.00 g, 0.053 mole, 1.0 eq), and *p*-TsOH•H_2O (a catalytic amount, about 0.24 g) were heated for 6 days at reflux in benzene (75 ml). The crude Schiff base **21i** (a brown oil) was taken up in hot Dowtherm A (8 ml, then 7 ml to rinse) and added to 100 ml of boiling Dowtherm A over 4 minutes, heating for a total of 15 minutes. After cooling, the reaction mixture was stirred with 350 ml of hexanes for 10 minutes. The

supernatant was decanted and the residue swirled with 70 ml ethyl acetate, followed by vacuum filtration, rinsing with ethyl acetate (200 ml) followed by acetone (2 x 10 ml). The pale yellow powder thus obtained, containing both the 5- and 7-chloro regioisomers, was converted to a pivalate ester to allow separation by chromatography. Sodium hydride (2.16 g of a 60% w/w dispersion in paraffin, thus 1.29 g, 0.054 mole, 2.0 eq of NaH) was deparaffinated by rinsing with hexanes (5 ml, then 2 x 2 ml) and taken up in anhydrous tetrahydrofuran (90 ml). The regioisomeric mixture from above (9.40 g, 0.027 mole of both regioisomers combined) was cautiously added over 10 minutes, and the reaction was then stirred at 60°C (sealed with a needle-vented septum) for 40 minutes. After removing from the heat, pivaloyl chloride (2.0 eq, 6.64 ml, 0.054 mole) was added cautiously over 5 minutes, followed by stirring at room temperature for 100 minutes (whereupon TLC showed that the reaction was complete). The reaction mixture was poured cautiously into ice water (100 ml) while stirring, then separated. The aqueous layer was extracted with 2 x 30 ml ethyl acetate, and the combined organic layers were evaporated under reduced pressure with warming (without drying) to remove most of the THF. The resulting biphasic yellow syrup was partitioned between water (75 ml) and ethyl acetate (75 ml) and separated, followed by extraction of the aqueous layer with ethyl acetate (2 x 25 ml). The pooled organic layers were rinsed with brine (50 ml), dried ($MgSO_4$), and evaporated under reduced pressure with warming. The residue was separated by automated flash chromatography on silica, eluting with a gradient of 99:1 to 92:8 v/v hexanes/ethyl acetate, to obtain the desired 7-chloro regioisomer (2-(4-bromophenyl)-7-chloro-3-methylquinolin-4-yl pivalate, R_f = 0.21, 95:5 v/v ethyl acetate/hexanes) as a white solid (5.40 g, 24% yield over several steps from the starting aniline). ^{1}H-NMR (400 MHz; $CDCl_3$): δ 8.12 (dd, *J* = 2.0, 0.5 Hz, 1H), 7.66 (dd, *J* = 8.9, 0.4 Hz, 1H), 7.65-7.62 (m, 2H), 7.49 (dd, *J* = 8.9, 2.0 Hz, 1H), 7.48-7.45 (m, 2H), 1.53 (d, *J* = 4.5 Hz, 9H)). To obtain the desired product, 2-(4-bromophenyl)-7-chloro-3-methylquinolin-4-yl pivalate (5.40 g, 0.012 mole) was dissolved in absolute ethanol by stirring at 70°C for 5 minutes. Water (5 ml) and *p*-TsOH•H_2O (0.037 mole, 7.12 g, 3.0 eq) were added, followed by heating at reflux for 22 hours, whereupon TLC showed completion. The reaction mixture was concentrated to 20 ml under reduced pressure with warming, poured into 130 ml water, stirred 30 minutes, and vacuum filtered, rinsing with excess water. The resulting white solid (4.58 g) still contained residual *p*-TsOH•H_2O, and so it was stirred in 70 ml water at 80°C for 1 hour, followed by filtration while still hot, rinsing with excess hot water followed by 2 x 3 ml acetone. This afforded the desired product **22i** as a white solid (4.18 g, 96% from the pivalate and 23% over several steps from the starting aniline). ^{1}H-NMR (400 MHz; DMSO-d_6): δ 11.67 (s, 1H), 8.12 (d, *J* = 8.7 Hz, 1H), 7.82-7.79 (m, 2H), 7.61-7.61 (m, 1H), 7.56-7.53 (m, 2H), 7.32 (dd, *J* = 8.7, 2.0 Hz, 1H), 1.87 (s, 3H).

2-(4-Bromophenyl)-4,6-dichloro-7-methoxy-3-methylquinoline (23a). Using General Procedure B, a stirred solution of **22a** (**HLQ-108**, 7.0 g, 18.5 mmol), $POCl_3$ (17.2 ml) and $CHCl_3$ (50 ml) was refluxed for 16 hours. After cooling to room temperature, the mixture was poured into ice water/$CHCl_3$ (200 ml/200 ml), and then concentrated aqueous NaOH was added slowly with stirring until pH ~14 for 2 hours. The mixture was filtered. The organic layer was separated, washed with water (50 ml x 2) and brine (50 ml), dried over ($MgSO_4$) and

concentrated to dryness under reduced pressure to give **23a** (6.34 g, 87 % yield) as a beige solid. and the precipitate was washed with water (3x50 ml) and air dried to give pure **23a** (735 mg, 71%) as a yellow solid. ^{1}H-NMR (400 MHz; $CDCl_3$): δ 8.25 (s, 1H), 7.66-7.62 (m, 2H), 7.52 (s, 1H), 7.45-7.41 (m, 2H), 4.03 (s, 3H), 2.49 (s, 3H).

2-(4-Bromophenyl)-4-chloro-5,7-difluoro-3-methylquinoline (23b). Using General Procedure B, a mixture of **22b** (6.08 g, 0.017 mole), chloroform (100 ml), and $POCl_3$ (0.052 mole, 4.86 g, 3.0 eq) was heated for 4 days. Workup was as described in the general procedure using a 300 ml ice bath together with 150 ml additional chloroform (stirring 10 minutes, then 10 minutes after basification), followed by extraction (3 x 50 ml chloroform and 75 ml brine). **23b** was obtained as a dull pale orange-yellow solid (5.98 g, 95% yield). ^{1}H-NMR (400 MHz; $CDCl_3$): δ 7.67-7.63 (m, 2H), 7.57 (dd, *J* = 7.8, 2.6 Hz, 1H), 7.45-7.42 (m, 2H), 7.14-7.08 (m, 1H), 2.49 (s, 3H), ^{19}F-NMR (376 MHz; CDCl3): δ -107.7 (m).

2-(4-Bromophenyl)-3-methylquinolin-4(1*H*)-one (23c). Using General Procedure B, a mixture of **22c** (2.30 g, 0.0073 mole), 100 ml chloroform (100 ml), and $POCl_3$ (3.0 eq, 0.022 mole, 3.37 g) was heated for 5 days. Workup was as described in the general procedure using a 350 ml ice bath (10 minutes stirring, then 5 minutes stirring after basification) followed by separation and extraction (3 x 40 ml chloroform and 50 ml brine), affording a beige solid (**23c**, 2.27 g, 93% yield). ^{1}H-NMR (400 MHz; $CDCl_3$): δ 8.25 (ddd, *J* = 8.4, 1.4, 0.5 Hz, 1H), 8.14 (br d, *J* = 8.3 Hz, 1H), 7.74 (ddd, *J* = 8.4, 6.9, 1.5 Hz, 1H), 7.67-7.62 (m, 3H), 7.47-7.44 (m, 2H), 2.53 (s, 3H).

2-(4-Bromophenyl)-4-chloro-7-methoxy-3-methylquinoline (23d). Using General Procedure B, a mixture of **22d** (2.31 g, 0.0067 mole), chloroform (80 ml), and $POCl_3$ (3.0 eq, 0.020 mole, 1.87 ml) was heated at reflux for 24 hours, followed by room temperature stirring for 5 days, then an additional 24 hours at reflux. Workup was as described in the general procedure using a 300 ml ice bath together with additional 120 ml chloroform (stirring one minute, then 10 minutes after basification), followed by separation and extraction (3 x 30 ml chloroform and 50 ml brine). This afforded 2.27 g of a beige solid with a sharp odor, which was partially recrystallized from 20 ml ethyl acetate, rinsing with 2 x 2 ml ethyl acetate, affording the desired product **23d** as a cream solid in sufficient purity for use in the following chlorination reaction (1.24 g, 51% yield). ^{1}H-NMR (400 MHz; $CDCl_3$): δ 8.12 (d, *J* = 9.2 Hz, 1H), 7.64-7.62 (m, 2H), 7.44-7.42 (m, 3H), 7.29-7.27 (m, partially overlaps $CDCl_3$ residual signal, estimated ~1H), 3.94 (s, 3H), 2.48 (s, 3H).

2-(4-Bromophenyl)-4-chloro-6-fluoro-7-methoxy-3-methylquinoline (23e): Using General Procedure B, a mixture of **22e** (4.00 g, 0.011 mole), chloroform (100 ml), and $POCl_3$ (0.033 mole, 5.06 g, 3.0 eq) was stirred at reflux for 2 days. Workup was as described in the general procedure using a 350 ml ice bath together with an additional 25 ml chloroform (stirring 3 minutes, then 10 minutes after basification), followed by extraction (3 x 40 ml chloroform and 50 ml brine), which afforded **23e** as a butter-yellow solid (3.92 g, 92% yield). ^{1}H-NMR (400 MHz; CDCl3): δ

7.85 (d, *JF* = 11.9 Hz, 1H), 7.65-7.62 (m, 2H), 7.53 (d, *JF* = 8.3 Hz, 1H), 7.44-7.41 (m, 2H), 4.02 (s, 3H), 2.49 (s, 3H); ^{19}F-NMR (376 MHz; CDCl3): δ -130.0 (m).

2-(4-Bromophenyl)-4,7-dichloro-3-methylquinoline (23i): Using General Procedure B, a mixture of **22i** (3.89 g, 0.011 mole), chloroform (90 ml), and $POCl_3$ (3.0 eq, 0.033 mole, 3.08 ml) was heated for 21 hours. Workup was as descirbed in the general procedure using a 350 ml ice bath together with 50 ml additional chloroform (stirring 5 minutes, then 30 minutes after basification), followed by extraction (3 x 40 ml chloroform and 60 ml brine). **23i** was obtained as an off-white solid (3.93 g, 97% yield). ^{1}H-NMR (400 MHz; $CDCl_3$): δ 8.17 (dd, *J* = 9.0, 0.4 Hz, 1H), 8.10 (dd, *J* = 2.1, 0.4 Hz, 1H), 7.66-7.63 (m, 2H), 7.58 (dd, *J* = 9.0, 2.1 Hz, 1H), 7.46-7.42 (m, 2H), 2.52 (s, 3H).

4,6-Dichloro-7-methoxy-3-methyl-2-(4-(4-(trifluoromethoxy)benzyl)phenyl)quinoline (24a). Using General Procedure C, a mixture of **23a** (2.5 mmol, 1 eq), 4-(trifluoromethoxy) benzylboronic acid pinacol ester (5 mmol, 2 eq), K_2CO_3 (5 mmol, 2 eq) and Pd(dppf)Cl_2 (0.25 mmol, 0.1 eq) in 12 mL toluene/dioxane/water (10:1:1) was stirred at 90°C for 24 hours. Worked up as described in the general procedure with 60 ml ethyl acetate and 50 ml water. The product was obtained as a white powder (0.522 g, 42.4% yield). ^{1}H-NMR (400 MHz; $CDCl_3$): δ 8.26 (s, 1H), 7.52-7.49 (m, 3H), 7.34-7.31 (m, 2H), 7.28-7.25 (m, 2H), 7.19-7.16 (m, 2H), 4.08 (s, 2H), 4.04 (s, 3H), 2.52 (s, 3H).

4-Chloro-5,7-difluoro-3-methyl-2-(4-(4-(trifluoromethoxy)benzyl)phenyl)quinoline (24b). Using General Procedure C, a mixture of **23b** (2 mmol, 1 eq), 4-(trifluoromethoxy) benzylboronic acid pinacol ester (4 mmol, 2 eq), K_2CO_3 (4 mmol, 2 eq) and Pd(dppf)Cl_2 (0.2 mmol, 0.1 eq) in 9 mL toluene/dioxane/water (10:1:1) was stirred at 90°C for 24 hours. Worked up as described in the general procedure with 60 ml ethyl acetate and 50 ml water. The product was obtained as a white powder (0.477 g, 51% yield). ^{1}H-NMR (400 MHz; $CDCl_3$): δ 7.60-7.56 (m, 1H), 7.51-7.47 (m, 2H), 7.33-7.29 (m, 2H), 7.27-7.22 (m, 2H), 7.21-7.05 (m, 3H), 4.06 (s, 2H), 2.50 (s, 3H).

4-Chloro-3-methyl-2-(4-(4-(trifluoromethoxy)benzyl)phenyl)quinoline (24c). Using General Procedure C, a mixture of **23c** (2 mmol, 1 eq), 4-(trifluoromethoxy) benzylboronic acid pinacol ester (4 mmol, 2 eq), K_2CO_3 (4 mmol, 2 eq) and Pd(dppf)Cl_2 (0.2 mmol, 0.1 eq) in 9 mL toluene/dioxane/water (10:1:1) was stirred at 90°C for 24 hours. Worked up as described in the general procedure with 60 ml ethyl acetate and 50 ml water. The product was obtained as a white powder (0.307 g, 36% yield). ^{1}H-NMR (400 MHz; $CDCl_3$): δ 8.24 (d, *J* = 8.6 Hz, 1H), 8.11 (d, *J* = 8.6 Hz, 1H), 7.74-7.68 (m, 1H), 7.65-7.59 (m, 1H), 7.51 (d, *J* = 8.2 Hz, 2H), 7.31 (d, *J* = 8.2 Hz, 2H), 7.25 (d, *J* = 8.2 Hz, 2H), 7.15 (d, *J* = 8.2 Hz, 2H), 4.06 (s, 2H), 2.54 (s, 3H).

4-Chloro-6-fluoro-7-methoxy-3-methyl-2-(4-(4-(trifluoromethoxy)benzyl)phenyl)quinoline (24e). Using General Procedure C, a mixture of **23e** (2 mmol, 1 eq), 4-(trifluoromethoxy) benzylboronic acid pinacol ester (4 mmol, 2 eq), K_2CO_3 (4 mmol, 2 eq) and Pd(dppf)Cl_2 (0.2 mmol, 0.1 eq) in 9 mL toluene/dioxane/water (10:1:1) was stirred

at 90°C for 24 hours. Worked up as described in the general procedure with 60 ml ethyl acetate and 50 ml water. The product was obtained as a white powder (0.495 g, 52% yield). ^{1}H-NMR (400 MHz; $CDCl_3$): δ 7.84 (d, *J* = 12.1 Hz, 1H), 7.52 (d, *J* = 8.2 Hz, 1H), 7.48 (d, *J* = 8.1 Hz, 1H), 7.30 (d, *J* = 8.2 Hz, 1H), 7.24 (d, *J* = 8.8 Hz, 1H), 7.15 (d, *J* = 8.4 Hz, 1H), 4.06 (s, 2H), 4.00 (s, 3H), 2.50 (s, 3H).

4,7-Dichloro-3-methyl-2-(4-(4-(trifluoromethoxy)benzyl)phenyl)quinoline (24i). Using General Procedure C, a mixture of **23i** (2 mmol, 1 eq), 4-(trifluoromethoxy) benzylboronic acid pinacol ester (4 mmol, 2 eq), K_2CO_3 (4 mmol, 2 eq) and Pd(dppf)Cl_2 (0.2 mmol, 0.1 eq) in 9 mL toluene/dioxane/water (10:1:1) was stirred at 90°C for 24 hours. Worked up as described in the general procedure with 60 ml ethyl acetate and 50 ml water. The product was obtained as a white powder (0.472 g, 51% yield). ^{1}H-NMR (400 MHz; $CDCl_3$): δ 8.19-8.09 (m, 1H), 7.58-7.47 (m, 2H), 7.34-7.05 (m, 8H), 4.07 (s, 2H), 2.53 (s, 3H).

6-Chloro-7-methoxy-3-methyl-2-(4-(4-(trifluoromethoxy)benzyl)phenyl)quinolin-4(1H)-one (25a, HLQ-168). Using General Procedure E, a mixture of **24a** (0.5 mmol, 1 eq) and anhydrous KOAc (5 mmol, 10 eq) in glacial AcOH (5 ml) was stirred at 120°C for 24 hours. Worked up as described in the general procedure with 20 ml ice water, 3 x 10 ml water, 2 x 10 ml acetone and 10 ml DCM. The product was obtained as a white powder (0.0315 g, 13% yield). ^{1}H-NMR (400 MHz; DMSO-d_6): δ 11.51 (s, 1H), 8.03 (s, 1H), 7.50-7.41 (m, 6H), 7.33-7.31 (m, 2H), 7.15 (s, 1H), 4.10 (s, 2H), 3.90 (s, 3H), 1.87 (s, 3H). HRMS calculated for C25H19ClF3NO3 [M + H]+ = 474.10783, observed for [M + H]+ = 474.10799.

5,7-Difluoro-3-methyl-2-(4-(4-(trifluoromethoxy)benzyl)phenyl)quinolin-4(1H)-one (25b, HLQ-172). Using General Procedure E, a mixture of **24b** (0.6 mmol, 1 eq) and anhydrous KOAc (6 mmol, 10 eq) in glacial AcOH (7.5 ml) was stirred at 120°C for 24 hours. Worked up as described in the general procedure with 20 ml ice water, 3 x 10 ml water, 2 x 10 ml acetone and 10 ml DCM. The product was obtained as an off-white powder (0.104 g, 39% yield). ^{1}H-NMR (400 MHz; DMSO-d_6): δ 7.50-7.40 (m, 6H), 7.32 (d, *J* = 8.2 Hz, 2H), 7.14-7.08 (m, 1H), 7.05-6.96 (m, 1H), 4.09 (s, 2H), 1.82 (s, 3H). HRMS calculated for C24H16F5NO2 [M + H]+ = 446.11739, observed for [M + H]+ = 446.11768.

3-Methyl-2-(4-(4-(trifluoromethoxy)benzyl)phenyl)quinolin-4(1H)-one (25c, HLQ-167). Using General Procedure E, a mixture of **24c** (0.671 mmol, 1 eq) and anhydrous KOAc (6.71 mmol, 10 eq) in glacial AcOH (7.5 ml) was stirred at 120°C for 24 hours. Worked up as described in the general procedure with 20 ml ice water, 3 x 10 ml water, 2 x 10 ml acetone and 10 ml DCM. The product was obtained as an off-white powder (0.245 g, 89% yield). ^{1}H-NMR (400 MHz; DMSO-d_6): δ 11.53 (s, 1H), 8.17-8.08 (m, 1H), 7.64-7.55 (m, 2H), 7.52-7.40 (m, 6H), 7.36-7.24 (m, 3H), 4.09 (s, 2H), 1.89 (s, 3H). HRMS calculated for C24H18F3NO2 [M + H]+ = 410.13624, observed for [M + H]+ = 410.13638.

6-Fluoro-7-methoxy-3-methyl-2-(4-(4-(trifluoromethoxy)benzyl)phenyl)quinolin-4(1H)-one (25e, HLQ-169). Using General Procedure E, a mixture of **24e** (1 mmol, 1 eq) and anhydrous KOAc (10 mmol, 10 eq) in glacial AcOH (7.5 ml) was stirred at 120°C for 24 hours. Worked up as described in the general procedure with 20 ml ice water, 3 x 10 ml water, 2 x 10 ml acetone and 10 ml DCM. The product was obtained as an off-white powder (0.245 g, 54% yield). ^{1}H-NMR (400 MHz; DMSO-d_6): δ 11.48 (s, 1H), 7.72 (d, *J* = 11.8 Hz, 1H), 7.50-7.41 (m, 6H), 7.32 (d, *J* = 8.2 Hz, 2H), 7.18 (d, *J* = 7.4 Hz, 1H), 4.09 (s, 2H), 3.89 (s, 3H), 1.87 (s, 3H). HRMS calculated for C25H19F4NO3 [M + H]+ = 458.13738, observed for [M + H]+ = 458.13763.

7-Chloro-3-methyl-2-(4-(4-(trifluoromethoxy)benzyl)phenyl)quinolin-4(1H)-one (25i, CK-2-68). Using General Procedure E, a mixture of **24i** (1.65 mmol, 1 eq) and anhydrous KOAc (16.5 mmol, 10 eq) in glacial AcOH (10 ml) was stirred at 120°C for 24 hours. Worked up as described in the general procedure with 20 ml ice water, 3 x 10 ml water, 2 x 10 ml acetone and 10 ml DCM. The product was obtained as an off-white powder (0.016 g, 2.2% yield). ^{1}H-NMR (400 MHz; DMSO-d_6): δ 11.57 (s, 1H), 8.11 (d, *J* = 8.6 Hz, 1H), 7.62-7.40 (m, 7H), 7.35-7.27 (m, 3H), 4.09 (s, 2H), 1.87 (s, 3H). HRMS calculated for C24H17ClF3NO2 [M + H]+ = 444.09726, observed for [M + H]+ = 444.09748.

4,6-Dichloro-7-methoxy-2-(4'-(trifluoromethoxy)-[1,1'-biphenyl]-4-yl)quinoline (26). Using General Procedure D, a mixture of **20** (766 mg, 2.0 mmol, 1 eq), (4-(trifluoromethoxy)phenyl)boronic acid (2.1 mmol, 1.05 eq), aqueous 2M K_2CO_3 (2 ml, 4.0 mmol, 2 eq) and Pd(dppf)Cl_2 (73 mg, 0.1 mmol, 0.05 eq) in DMF (100 ml) was heated for 66 hours until no more starting material remained, as determined by GC-MS and TLC. After filtration and evaporation as in the general procedure, the resulting black, oily solid was resuspended in DCM (100 ml) and stirred vigorously at room temperature for 30 minutes, filtered through Celite, and concentrated to dryness under reduced pressure to give crude **26** (942 mg). Flash chromatography, using a gradient of ethyl acetate/hexanes, gave **26** (592 mg) as a yellow solid. GC-MS and NMR showed that **26** contained 5-10% of another product with m/z = 589 corresponding to the addition of 4-(trifluoromethoxy)phenyl)boronic acid at the 4 position of **26**. This product was used without further purification to make **27** (**HLQ-104**).

6-Chloro-7-methoxy-2-(4'-(trifluoromethoxy)-[1,1'-biphenyl]-4-yl)quinolin-4(1*H*)-one (27, HLQ-104). Using General Procedure E, a mixture of **26** (592 mg, 1.28 mmol, 1.0 eq), KOAc, (1.25 g, 12.8 mmol, 10.0 eq) and glacial acetic acid (5 ml) was heated at 120°C for 18 hours. After cooling to room temperature, the reaction mixture was poured into ice water (30 ml). The resulting precipitate was filtered and washed with water (3 x 20 ml), acetone (3 x 10 ml), DCM (3 x 10 ml), hexanes (3 x 10 ml) and air-dried to give pure **27** (**HLQ-104**) as a white solid (372 mg, 65% yield, mp 349.5-350 °C (dec)). ^{1}H-NMR (400 MHz; DMSO-d_6): δ 11.75 (s, 1H), 8.04 (s, 1H), 7.98-7.91 (m, 6H), 7.53-7.50 (m, 2H), 7.41 (s, 1H), 6.42 (s, 1H), 3.98 (s, 3H). HRMS calculated for C23H15CLF3NO3 [M + H]+ = 446.07653, observed for [M + H]+ = 446.07653.

4,6-Dichloro-7-methoxy-3-methyl-2-(4'-(trifluoromethoxy)-[1,1'-biphenyl]-4-yl)quinoline (28a). Using General Procedure D, a stirred mixture of **23a** (735 mg, 1.85 mmol, 1.0 eq), (4-(trifluoromethoxy)phenyl)boronic acid (419 mg, 2.04 mmol, 1.1 eq), aqueous 2M K_2CO_3 (1.85 ml, 3.7 mmol, 2 eq), and Pd(dppf)Cl_2 (68 mg, 0.1 mmol, 0.05 eq) was heated in DMF (75 ml) for 16 hours until no more starting material remained, as determined by GC-MS and TLC. After filtration and evaporation as described in the general procedure, the resulting black, oily solid was resuspended in DCM (200 ml), stirred vigorously at room temperature for 30 minutes, filtered through Celite, and concentrated to dryness under reduced pressure to give crude **28a** (880 mg). Chromatography using a gradient of ethyl acetate/hexanes gave **28a** (325 mg, 37% yield) as a white solid. ^{1}H-NMR (400 MHz; $CDCl_3$): δ 8.29 (s, 1H), 7.73-7.65 (m, 6H), 7.55 (s, 1H), 7.36-7.34 (m, 2H), 4.06 (s, 3H), 2.58 (s, 3H).

4-Chloro-5,7-difluoro-3-methyl-2-(4'-(trifluoromethoxy)-[1,1'-biphenyl]-4-yl)quinoline (28b). Using General Procedure D, a mixture of **23b** (1.00 g, 0.0027 mole), *para*-(trifluoromethoxy)phenyl)boronic acid (0.67 g, 0.0033 mole, 1.2 eq), DMF (80 ml), and aqueous potassium carbonate (0.75 g of anhydrous potassium carbonate dissolved in 2.7 ml water, 0.0054 mol, 2.0 eq) was degassed for 20 minutes. Pd(dppf)Cl_2 (0.10 g, 0.00014 mol, 0.05 eq) was added followed by heating for 3 days. Workup was as described in the general procedure using 125 ml of DCM. Chromatography used an elution gradient of 100:0 to 95:5 v/v hexanes/ethyl acetate, affording the desired product **28b** as a white solid (0.77 g, 63% yield, Rf = 0.35 (95:5 v/v hexanes/ethyl acetate)). ^{1}H-NMR (400 MHz; $CDCl_3$): δ 7.74-7.65 (m, 6H), 7.63 (ddd, *J* = 9.3, 2.6, 1.5 Hz, 1H), 7.36-7.34 (m, 2H), 7.13 (ddd, *J* = 12.1, 8.8, 2.6 Hz, 1H), 2.58 (s, 3H); 19-F NMR (376 MHz; CDCl3): δ -57.8 (s, 3F), -107.8 (m, 2F).

4-Chloro-3-methyl-2-(4'-(trifluoromethoxy)-[1,1'-biphenyl]-4-yl)quinoline (28c). Using General Procedure D, a mixture of **23c** (1.2 eq, 0.0048 mole, 1.00 g), DMF (105 ml), and aqueous potassium carbonate (1.11 g of anhydrous potassium carbonate dissolved in 4.0 ml water, 0.0080 mol, 2.0 eq) was degassed for 30 minutes. 1,1'-Pd(dppf)Cl_2 (0.15 g, 0.00020 mol, 0.05 eq) was added followed by 21 hours of heating. Workup was as described in the general procedure, using 120 ml DCM. Chromatographic purification used an elution gradient of 100:0 to 93:7 v/v hexanes/ethyl acetate. The desired product **28c** was obtained as a white solid (1.03 g, 62% yield, R_f = 0.50 (9:1 v/v hexanes/ethyl acetate)). ^{1}H-NMR (400 MHz; $CDCl_3$): δ 8.26 (dd, *J* = 8.4, 1.0 Hz, 1H), 8.15-8.12 (m, 1H), 7.75-7.62 (m, 8H), 7.34-7.30 (m, 2H), 2.60 (s, 3H), ^{19}F-NMR (376 MHz; CDCl3): δ -57.8).

4-Chloro-7-methoxy-3-methyl-2-(4'-(trifluoromethoxy)-[1,1'-biphenyl]-4-yl)quinoline (28d). Using General Procedure D, a mixture of **23d** (1.24 g 0.0034 mole), *para*-(trifluoromethoxy)phenyl)boronic acid (0.84 g, 0.0041 mole, 1.2 eq), DMF (100 ml), and aqueous potassium carbonate (0.94 g of anhydrous potassium carbonate dissolved in 3.4 ml water, 0.0068 mol, 2.0 eq) was degassed for 20 minutes. Pd(dppf)Cl_2 (0.12 g, 0.00017 mol, 0.05 eq) was added followed by 20 hours of heating. Workup was as described in the general procedure, using 110 ml of DCM. For chromatography, an elution gradient of 100:0 to 87:13 v/v hexanes/ethyl acetate was used, affording the

desired product **28d** as a white solid (1.08 g, 72% yield, $R_f = 0.40$ (9:1 v/v hexanes/ethyl acetate)). ^{1}H-NMR (400 MHz; $CDCl_3$): δ 8.13 (d, *J* = 9.2 Hz, 1H), 7.70-7.63 (m, 6H), 7.46 (d, *J* = 2.5 Hz, 1H), 7.34-7.31 (m, 2H), 7.28 (dd, *J* = 9.2, 2.6 Hz, 1H), 3.95 (s, 3H), 2.55 (s, 3H); ^{19}F-NMR (376 MHz; CDCl3): δ -57.8 (s).

4-Chloro-6-fluoro-7-methoxy-3-methyl-2-(4'-(trifluoromethoxy)-[1,1'-biphenyl]-4-yl)quinoline (28e). Using General Procedure D, a mixture of **23e** (1.05 g 0.0027 mole), *para*-(trifluoromethoxy)phenyl)boronic acid (0.67 g, 0.0033 mole, 1.2 eq), DMF (80 ml), and aqueous potassium carbonate (0.75 g of anhydrous potassium carbonate dissolved in 2.7 ml water, 0.0054 mol, 2.0 eq) was degassed for 20 minutes. Pd(dppf)Cl_2 (0.10 g, 0.00014 mol, 0.05 eq) was added followed by heating for 3 days. The workup differed from the general procedure. The cooled reaction mixture was vacuum filtered to remove solids, followed by concentration of the filtrate under reduced pressure with heating. The resulting dark residue was swirled with DCM (125 ml) and again filtered (Filtrate A). Meanwhile, the solids recovered from the initial filtration contained crystals that were not water soluble. These were taken up in 20 ml of DCM and rinsed with water (20 ml). The aqueous layer was extracted with an additional 15 ml of DCM, and the pooled organic layers were dried ($MgSO_4$) and filtered (Filtrate B). Filtrate B was combined with Filtrate A from above and evaporated. Chromatography used an elution gradient of 100:0 to 87:13 v/v hexanes/ethyl acetate and afforded the desired product **28e** as a white solid (0.80 g, 63% yield, $R_f = 0.46$ (8:2 v/v hexanes/ethyl acetate)). ^{1}H-NMR (400 MHz; $CDCl_3$): δ 7.86 (d, J_F = 12.0 Hz, 1H), 7.70-7.62 (m, 6H), 7.55 (d, J_F = 8.2 Hz, 1H), 7.35-7.31 (m, 2H), 4.02 (s, 3H), 2.55 (s, 3H); ^{19}F-NMR (376 MHz; CDCl3): δ -57.8 (s, 3F), -130.5 (dd, 1F, J_H = 12.3, 8.3 Hz).

4,7-Dichloro-3-methyl-2-(4'-(trifluoromethoxy)-[1,1'-biphenyl]-4-yl)quinoline (28i). Using General Procedure D, a mixture of **23i** (0.50 g, 0.0014 mole), *para*-(trifluoromethoxy)phenyl)boronic acid (0.33 g, 0.0016 mole, 1.2 eq), DMF (80 ml), and aqueous potassium carbonate (0.39 g of anhydrous potassium carbonate dissolved in 1.6 ml water, 0.0028 mol, 2.0 eq) was degassed for 20 minutes. Pd(dppf)Cl_2 (0.051 g, 0.00007 mol, 0.05 eq) was added followed by heating for 22 hours. Workup was as described in the general procedure using 125 ml of DCM. Chromatography used an elution gradient of 99:1 to 98:2 v/v hexanes/ethyl acetate, affording a mixture containing the desired product **28i** and an impurity resulting from double addition of the boronic ester to the starting quinoline in a ratio of about 3:1 mole/mole, as estimated by NMR (both compounds observable by GC-MS: $(M-H)^+ = 446.2$, $t_R = 12.665$ min., and $(M-H)^+ = 572.2$, $t_R = 15.861$ min., temperature program starting at 75°C). This mixture was used without further purification in the subsequent chloride replacement reaction (see **2i**).

4,6-Dichloro-7-methoxy-3-methyl-2-(3'-(trifluoromethoxy)-[1,1'-biphenyl]-4-yl)quinoline (29). Using General Procedure D, a mixture of **23a** (0.70 g 0.0018 mole), *meta*-(trifluoromethoxy)phenyl)boronic acid (0.44 g, 0.0021 mole, 1.2 eq), DMF (80 ml), and K_2CO_3 (0.50 g of anhydrous K_2CO_3dissolved in 1.8 ml water, 0.0036 mol, 2.0 eq) was degassed for 20 minutes. Pd(dppf)Cl_2 (0.066 g, 0.000090 mole, 0.05 eq) was added followed by heating for 4

days. Workup was as described in the general procedure, using 125 ml of DCM. Chromatography used an elution gradient of 100:0 to 92:8 v/v hexanes/ethyl acetate, affording the desired product **29** as a white solid (0.50 g, 58% yield, R_f = 0.56 (8:2 v/v hexanes/ethyl acetate)). ^{1}H-NMR (400 MHz; $CDCl_3$): δ 8.26 (s, 1H), 7.73-7.70 (m, 2H), 7.66-7.63 (m, 2H), 7.60-7.57 (m, 1H), 7.52-7.48 (m, 3H), 7.26-7.23 (m, 1H), 4.04 (s, 3H), 2.55 (s, 3H); ^{19}F-NMR (376 MHz; CDCl3): δ -57.7 (s).

6-Chloro-7-methoxy-3-methyl-2-(3'-(trifluoromethoxy)-[1,1'-biphenyl]-4-yl)quinolin-4(1*H*)-one (30, HLQ-140). Using General Procedure E, a mixture of **29** (0.50 g, 0.0010 mole), anhydrous potassium acetate (1.03 g, 0.010 mole, 10 eq), and glacial acetic acid (10 ml) was heated at 120°C for 20 hours. The hot reaction mixture was poured into water (100 ml). The resulting precipitate was collected by vacuum filtration, rinsing with excess water followed by acetone (30 ml) to afford an off-white powder, the desired product **30** (**HLQ-140**, 0.39 g, 85% yield). ^{1}H-NMR (400 MHz; DMSO-d_6): δ 11.63 (s, 1H), 8.06 (s, 1H), 7.96-7.93 (m, 2H), 7.84 (ddd, *J* = 7.8, 1.6, 0.9 Hz, 1H), 7.75-7.70 (m, 1H), 7.69-7.66 (m, 3H), 7.45-7.43 (m, 1H), 7.20 (s, 1H), 3.92 (s, 3H), 1.93 (s, 3H). ^{19}F-NMR (376 MHz; DMSO): δ -56.6 (s). HRMS calculated for C24H17ClF3NO3 [M + H]+ = 460.09218, observed for [M + H]+ = 460.09249.

((6-Chloro-7-methoxy-3-methyl-2-(4-(4-(trifluoromethoxy)phenoxy)phenyl)quinolin-4-yl)oxy)methyl ethyl carbonate (31, HLQ-130). Using General Procedure F, a mixture of **1a** (**HLQ-102**, 1.00g, 0.0021 mole), tetrabutylammonium iodide (0.0042 mole, 1.56 g, 2.0 eq), anhydrous K_2CO_3 (0.58 g, 0.0042 mole, 2.0 eq), 30 ml *N,N*-dimethylformamide, and chloromethyl ethyl carbonate (0.58 g, 0.0042 mole, 2.0 eq) was heated for 21 hours. Workup was as described in the general procedure, using 50 ml ethyl acetate. Chromatography utilized an elution gradient of 95:5 to 88:12 v/v hexanes/ethyl acetate, affording the desired product (R_F = 0.29, 8:2 v/v hexanes/ethyl acetate) as a white powder (**31**, **HLQ-130**, 0.70 g, 58% yield). ^{1}H-NMR (400 MHz; $CDCl_3$): δ 8.01 (s, 1H), 7.59-7.56 (m, 2H), 7.51 (s, 1H), 7.23-7.20 (m, 2H), 7.15-7.11 (m, 2H), 7.10-7.06 (m, 2H), 5.84 (s, 2H), 4.25 (q, *J* = 7.1 Hz, 2H), 4.02 (s, 3H), 2.38 (s, 3H), 1.31 (t, *J* = 7.1 Hz, 3H); ^{19}F-NMR (376 MHz; CDCl3): δ -58.2). ^{13}C-NMR (101 MHz; $CDCl_3$): δ 162.0, 157.9, 157.1, 155.9, 155.5, 154.2, 147.9, 144.7, 136.1, 130.7, 124.9, 122.7, 122.3, 119.9, 119.4, 118.8, 117.8, 108.9, 91.6, 64.9, 56.4, 14.2. HRMS calculated for C28H23ClF3NO7 [M + H]+ = 578.11879, observed for [M + H]+ = 578.11937.

6-Chloro-4-(((ethoxycarbonyl)oxy)methoxy)-7-methoxy-3-methyl-2-(4-(4-(trifluoromethoxy)phenoxy)-phenyl)quinoline 1-oxide (32). Using General Procedure G, a solution of **31** (**HLQ-130**, 578 mg, 1.0 mmol, 1 eq), *m*CPBA (346 mg, 2.0 mmol, 2 eq), and $CHCl_3$ (25 ml) was heated at 90°C for 24 hours. The product was purified by flash chromatography using a gradient of hexanes/ethyl acetate to give **32** (290 mg, 49% yield) as a white solid. ^{1}H-NMR (400 MHz; $CDCl_3$): δ 8.20 (s, 1H), 8.05 (s, 1H), 7.43-7.39 (m, 2H), 7.24-7.22 (m, 2H), 7.18-7.10 (m, 4H), 5.78 (s, 2H), 4.22 (q, *J* = 7.1 Hz, 2H), 4.06 (s, 3H), 2.18 (s, 3H), 1.28 (t, *J* = 7.1 Hz, 3H).

6-Chloro-1-hydroxy-7-methoxy-3-methyl-2-(4-(4-(trifluoromethoxy)phenoxy)phenyl)quinolin-4(1*H*)-one (33, HLQ-103). Using General Procedure H, a mixture of **32** (198 mg, 0.3 mmol), methanol/10% aqueous NaOH (4:1, 10 ml) was heated for 16 hours. Pure **33** (**HLQ-103**, 138 mg, 93% yield) was obtained as a yellow solid. ^{1}H-NMR (400 MHz; DMSO-d_6): δ 8.07 (s, 1H), 7.73 (s, 1H), 7.43 (d, *J* = 8.6 Hz, 2H), 7.31-7.26 (m, 2H), 7.20-7.17 (m, 2H), 7.07-7.05 (m, 2H), 3.84 (s, 3H), 1.76 (s, 3H).

((6-Chloro-7-methoxy-3-methyl-2-(4'-(trifluoromethoxy)-[1,1'-biphenyl]-4-yl)quinolin-4-yl)oxy)methyl ethyl carbonate (34, HLQ-114). Using General Procedure F, a mixture of **2a** (**HLQ-105**, 956 mg, 2.0 mmol, 1 eq), TBAI (1.48 g, 4.0 mmol, 2.0 eq), chloromethyl ethyl carbonate (556 mg, 4.0 mmol, 2.0 eq) and dry K_2CO_3 (556 mg, 2.0 mmol, 2 eq) in DMF (150 ml) was heated for 18 hours. Workup was as described in the general procedure, using 150 ml ethyl acetate and stirring for 30 minutes, then washing with 50 ml ethyl acetate to afford 1.13 g of a yellow solid. Chromatography using a gradient of ethyl acetate/hexane as eluent gave **34** (910 mg) as a white solid. The product was further purified by crystallization from hexanes to give pure **34** (**HLQ-105**, 642 mg, 57% yield) as white needles. ^{1}H-NMR (400 MHz; $CDCl_3$): δ 8.05 (s, 1H), 7.72-7.67 (m, 6H), 7.56 (s, 1H), 7.36-7.33 (m, 2H), 5.88 (s, 2H), 4.28 (q, *J* = 7.1 Hz, 2H), 4.05 (s, 3H), 2.43 (s, 3H), 1.33 (t, *J* = 7.1 Hz, 3H). ^{13}C-NMR (101 MHz; $CDCl_3$): δ 162.3, 157.9, 155.9, 154.3, 148.8, 148.0, 139.93, 139.91, 139.5, 129.5, 128.5, 127.1, 125.0, 122.3, 121.3, 119.4, 117.9, 108.9, 91.7, 64.9, 56.4, 14.1. HRMS calculated for C28H23ClF3NO6 [M + H]+ = 562.12387, observed for [M + H]+ = 562.12434.

6-Chloro-4-(((ethoxycarbonyl)oxy)methoxy)-7-methoxy-3-methyl-2-(4'-(trifluoromethoxy)-[1,1'-biphenyl]-4-yl)quinoline 1-oxide (35). Using General Procedure G, a solution of **34** (**HLQ-114**, 1.28 g, 2.28 mmol, 1 eq), *m*CPBA (592 mg, 3.42 mmol, 2 eq), and $CHCl_3$ (50 ml) was heated at 90°C for 12 hours, TLC still showed some starting material left. Two additional equivalents of *m*CPBA (592 mg) were added and heating was continued for another 12 hours. The product was purified by flash chromatography using a gradient of hexanes/ethyl acetate to give **35** (830 mg, 63% yield), as a brown solid. The product was pure enough for the next step. A sample was crystallized in hexanes/ethyl acetate to give a pure sample as a white solid for NMR analysis. ^{1}H-NMR (400 MHz; $CDCl_3$): δ 8.23 (s, 1H), 8.09 (s, 1H), 7.77-7.75 (m, 2H), 7.71-7.69 (m, 2H), 7.54-7.51 (m, 2H), 7.36-7.33 (m, 2H), 5.82 (s, 2H), 4.25 (q, *J* = 7.1 Hz, 2H), 4.09 (s, 3H), 2.22 (s, 3H), 1.31 (t, *J* = 7.1 Hz, 3H).

6-Chloro-1-hydroxy-7-methoxy-3-methyl-2-(4'-(trifluoromethoxy)-[1,1'-biphenyl]-4-yl)quinolin-4(1*H*)-one (36, HLQ-106). Using General Procedure H, a mixture of **35** (577 mg, 1.0 mmol), ethanol/10% aqueous NaOH (4:1, 30 ml) was heated for 16 hours. Pure **36** (**HLQ-106**, 390 mg, 82% yield) was obtained as a yellow solid. ^{1}H-NMR (400 MHz; $CDCl_3$): δ 8.23 (s, 1H), 8.09 (s, 1H), 7.77-7.75 (m, 2H), 7.71-7.69 (m, 2H), 7.54-7.51 (m, 2H), 7.36-7.33 (m, 2H), 5.82 (s, 2H), 4.25 (q, *J* = 7.1 Hz, 2H), 4.09 (s, 3H), 2.22 (s, 3H), 1.31 (t, *J* = 7.1 Hz, 3H). HRMS calculated for C24H17ClF3NO4 [M + H]+ = 476.08709, observed for [M + H]+ = 476.08723.

ABBREVIATIONS

AcOH – Glacial acetic acid
$CHCl_3$- Chloroform
DCM - Dichloromethane
(dec) – Decomposition during melting
DMF – N,N-dimethylformamide
GC-MS – Gas chromatography-mass spectroscopy
HRAM – High-resolution accurate mass
K_2CO_3-potassium carbonate
KOAc – potassium acetate,
60% oil suspension of sodium hydride- 60% NaH
mCPBA – *meta*-Chloroperbenzoic acid
mp – Melting point
NMR – Nuclear magnetic resonance spectroscopy
Pd(dppf)Cl_2 - [1,1'-bis(Diphenylphosphino)ferrocene]-dichloropalladium (II)
$POCl_3$ – Phosphorus oxychloride
TBAI – Tetrabutylammonium iodide
TLC- Thin layer chromatography
p-TsOH•H_2O – *para*-Toluenesulfonic acid monohydrate

^{1}H-NMR: 1a (HLQ-102)

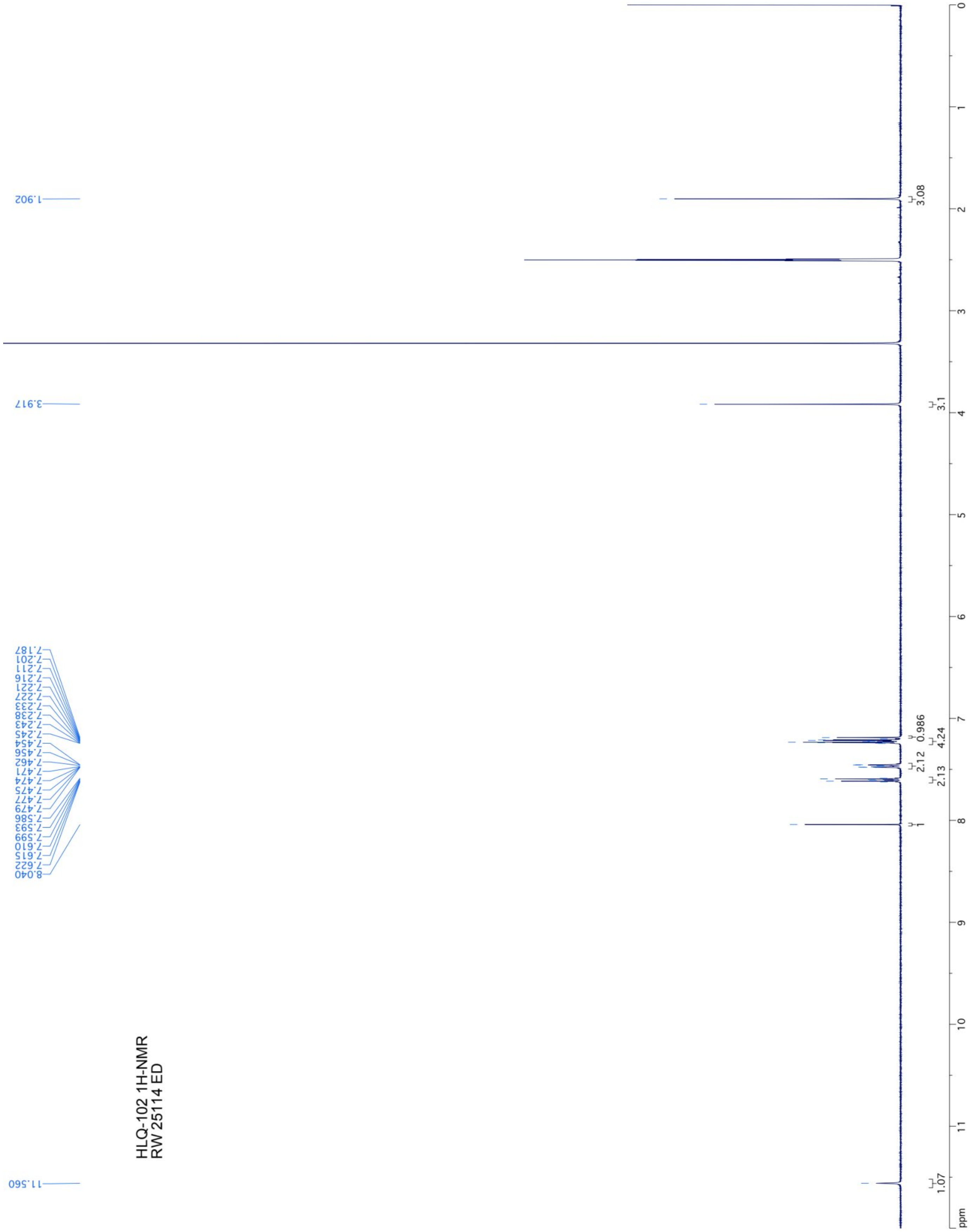

^{1}H-NMR: **2a** (HLQ-105)

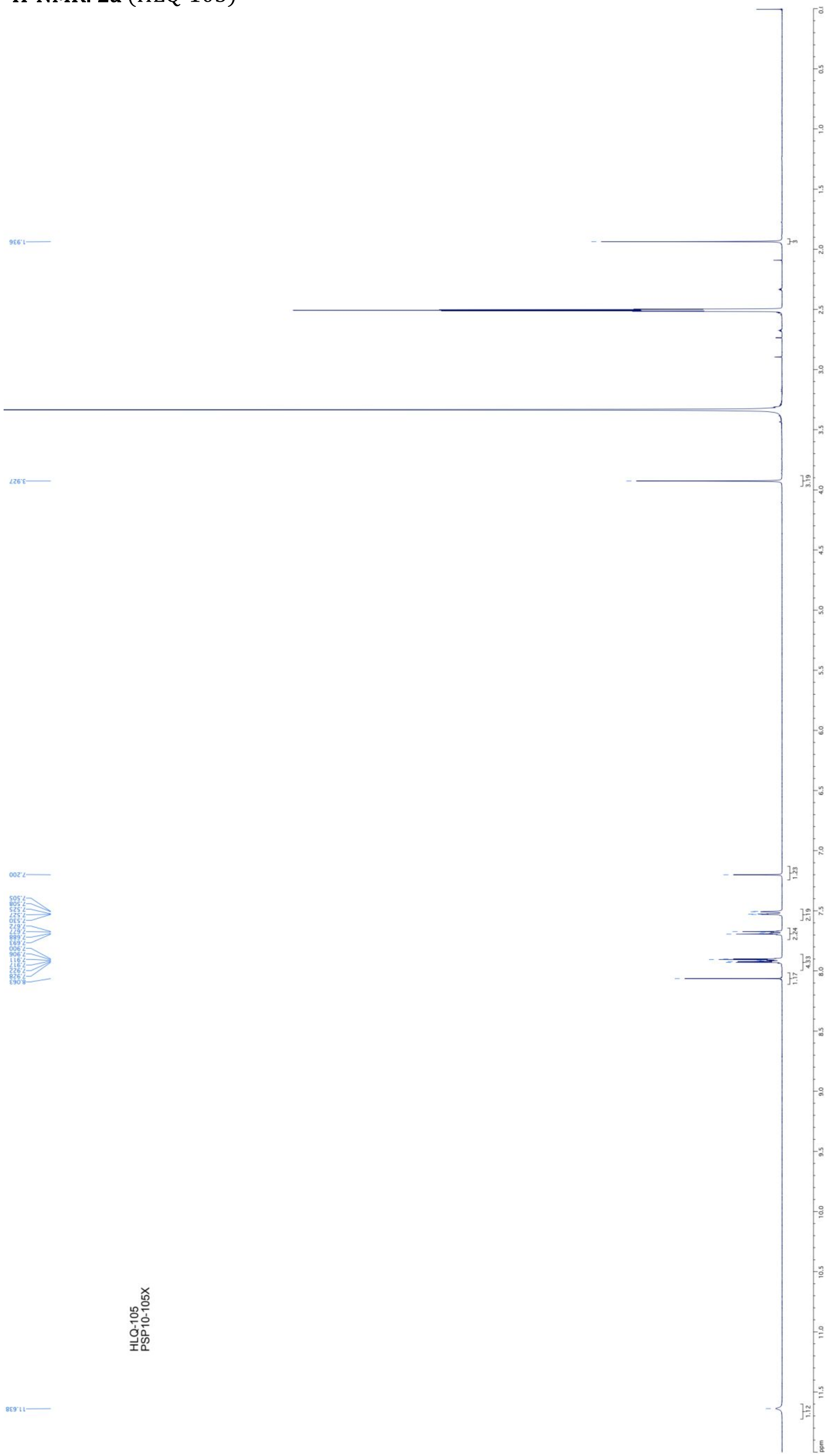

¹H-NMR: 34 (HLQ-114)

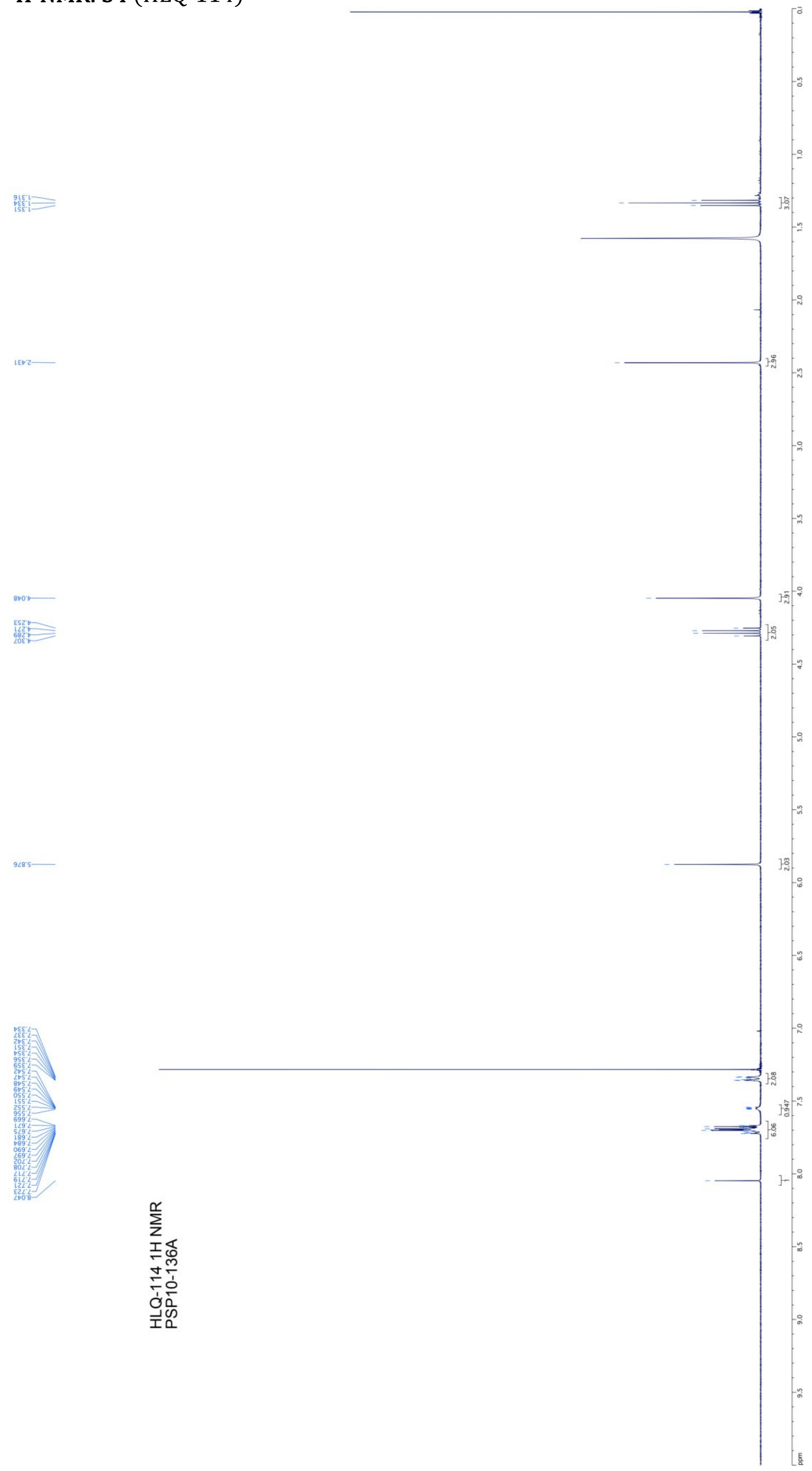

^{1}H-NMR: 31 (HLQ-130)

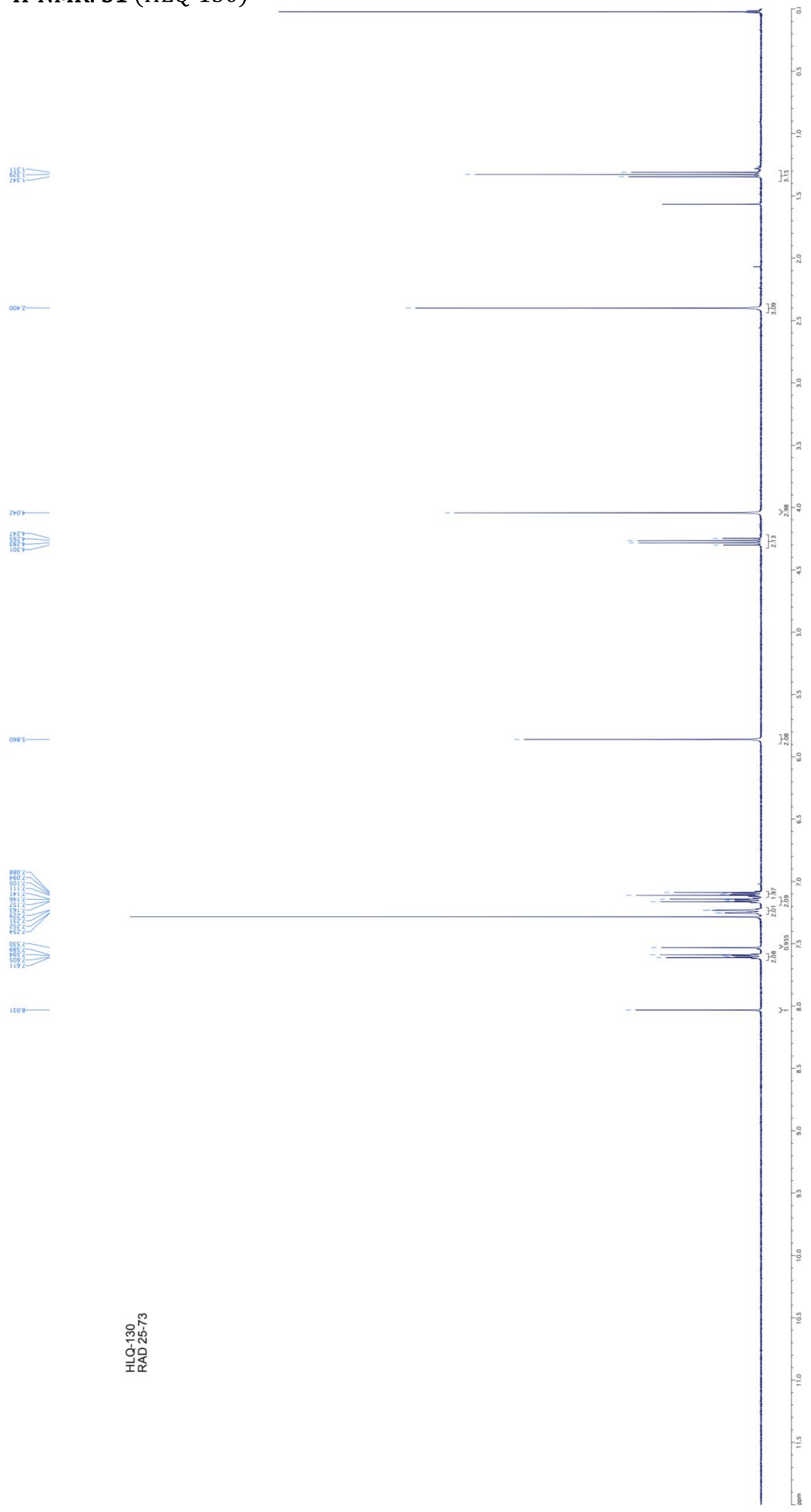

^{13}C-NMR: 1a (HLQ-102)

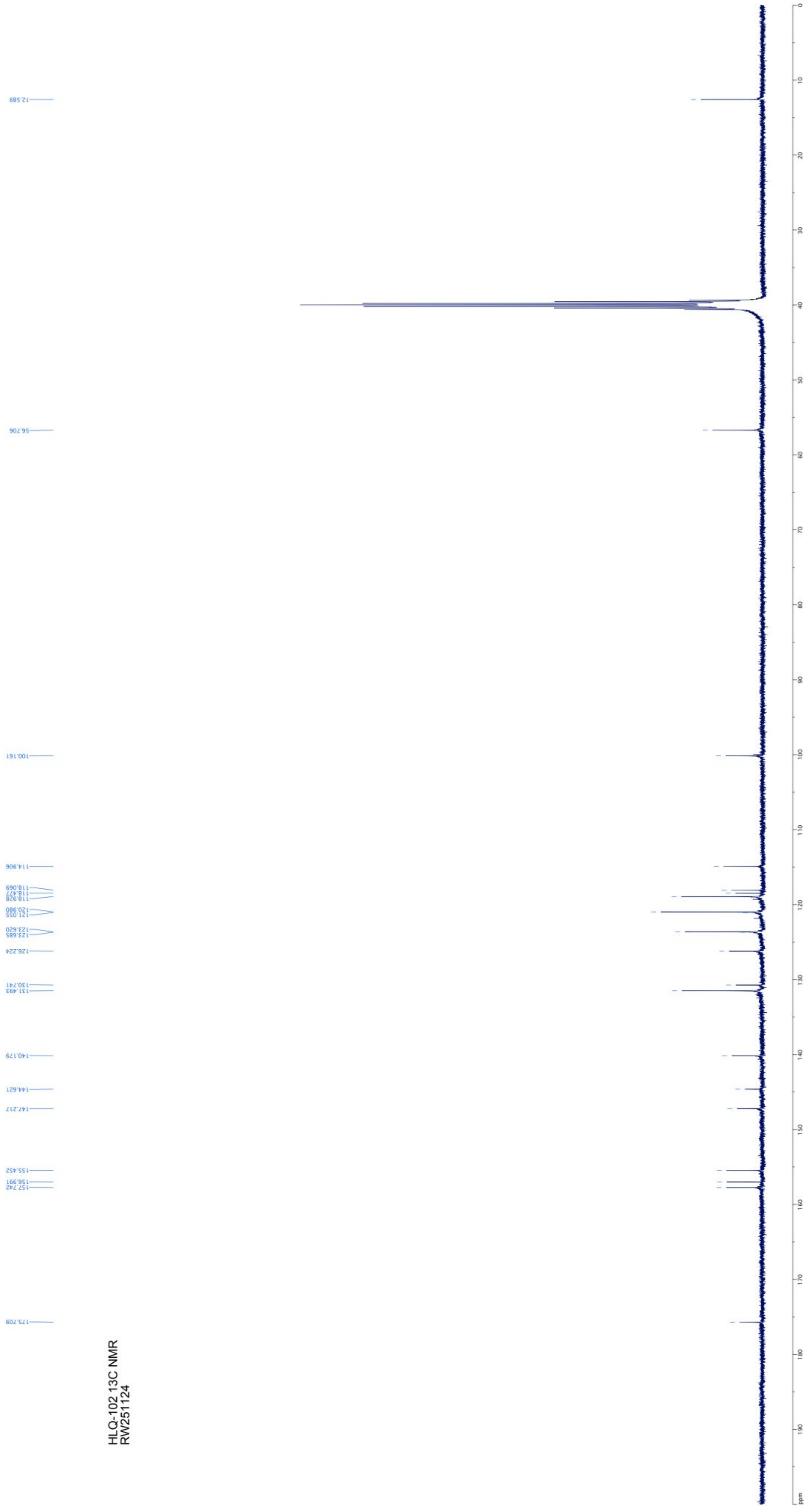

^{13}C-NMR: 2a (HLQ-105)

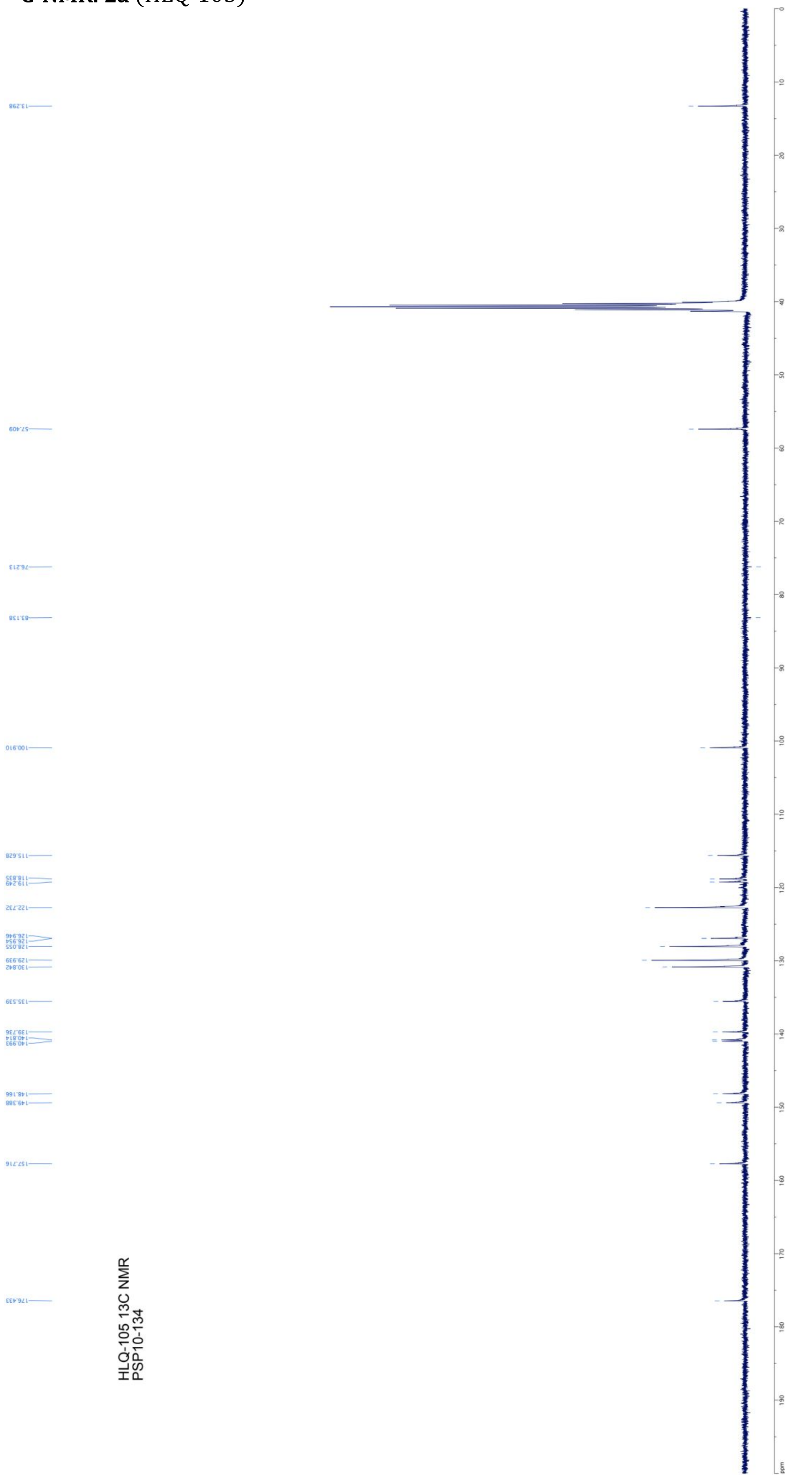

^{13}C-NMR: 34 (HLQ-114)

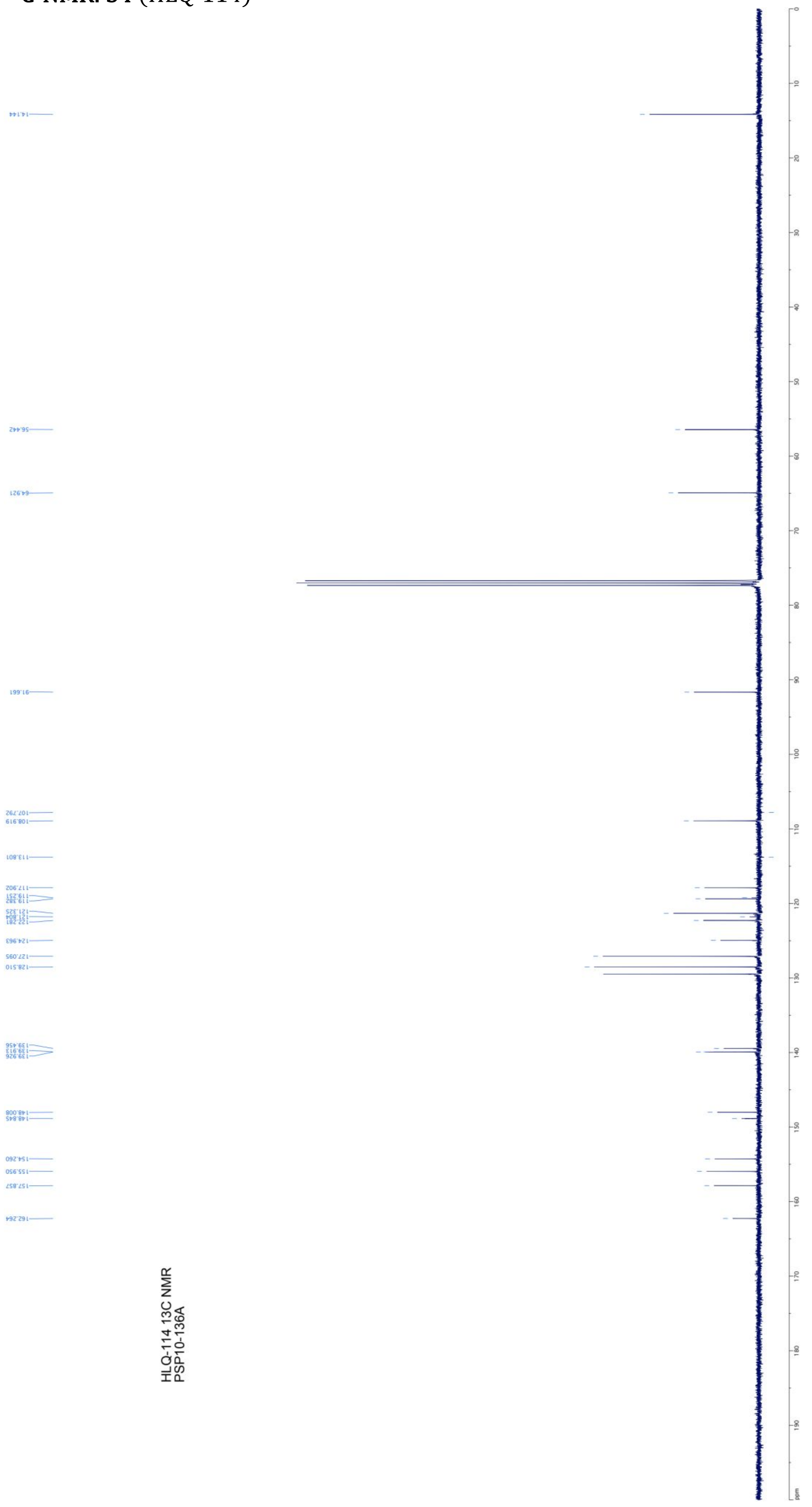

^{13}C-NMR: 31 (HLQ-130)

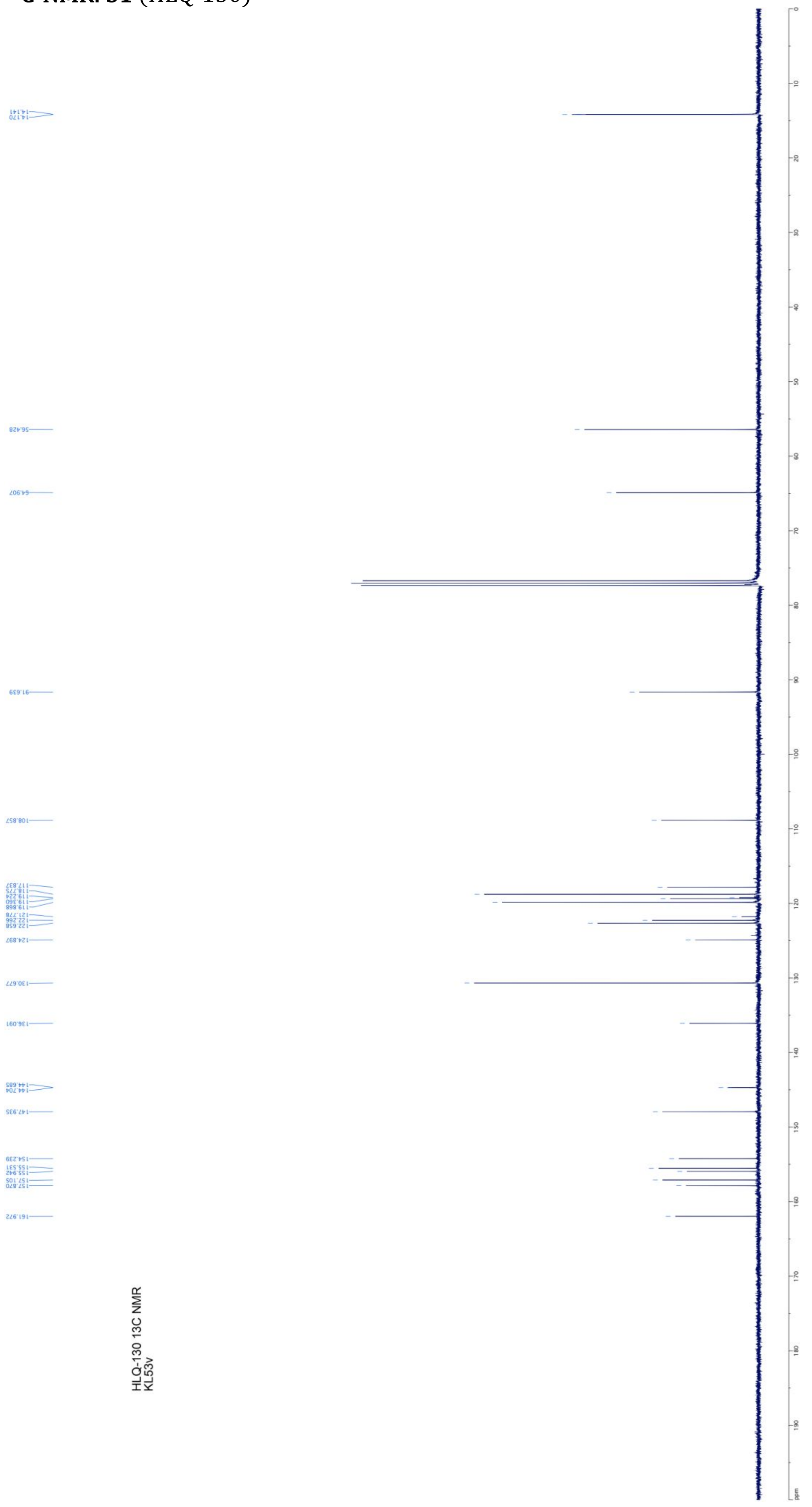

HPLC Data:

1a (HLQ-102)

Sample Name	HLQ-102 RWW 25.11.24	Sequence Acquired Date	8/5/2025 11:27:47 AM (GMT -07:00)

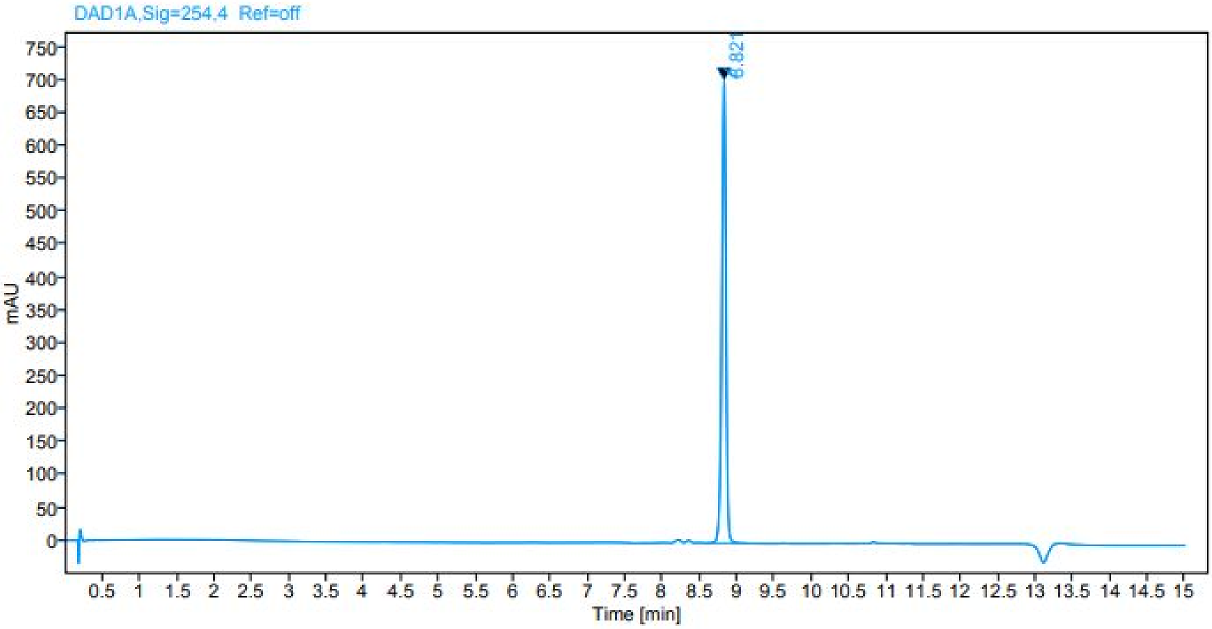

Signal: DAD1A,Sig=254,4 Ref=off

RT [min]	Type	Width [min]	Area	Height	Area%	Name
8.821	BB	0.4741	2684.6008	702.3006	100.0000	
		Sum	2684.6008			

2a (HLQ-105)

Sample Name	HLQ-105<br>PSP10-134	Sequence Acquired Date	8/5/2025<br>12:56:15 PM<br>(GMT -07:00)

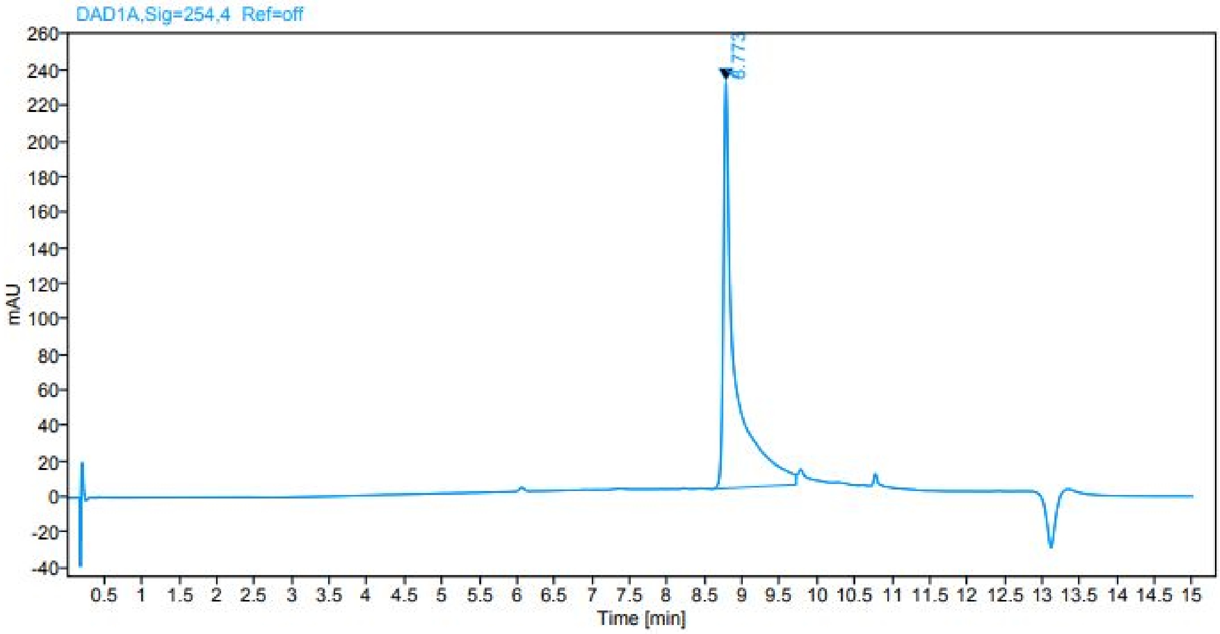

Signal: DAD1A,Sig=254,4 Ref=off

RT [min]	Type	Width [min]	Area	Height	Area%	Name
8.773	BV	1.1192	2432.4197	228.6212	100.0000	
		Sum	2432.4197			

34 (HLQ-114)

Sample Name	HLQ-114<br>PSP10-136A	Sequence Acquired Date	8/25/2025<br>9:47:36 PM<br>(GMT -07:00)

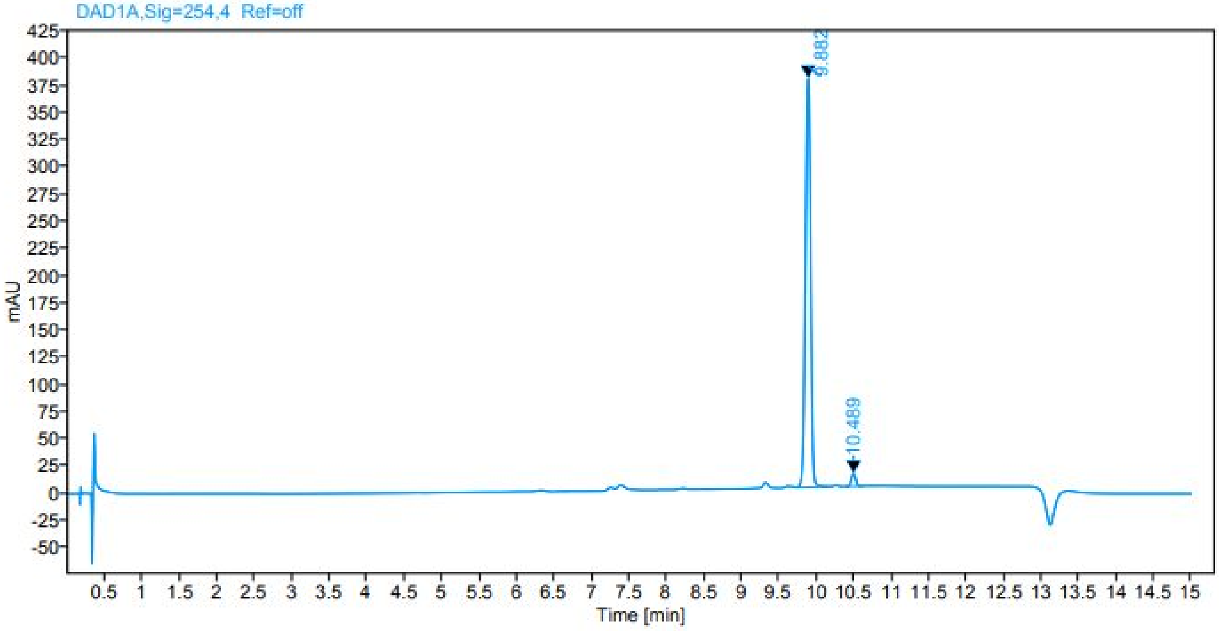

Signal: DAD1A,Sig=254,4 Ref=off

RT [min]	Type	Width [min]	Area	Height	Area%	Name
9.882	VB	0.4352	1776.7627	375.4484	97.5321	
10.489	BB	0.2069	44.9591	11.5782	2.4679	
		Sum	1821.7218			

31 (HLQ-130)

Sample Name	HLQ-130<br>KL53V	Sequence Acquired Date	8/25/2025<br>10:52:41 PM<br>(GMT -07:00)

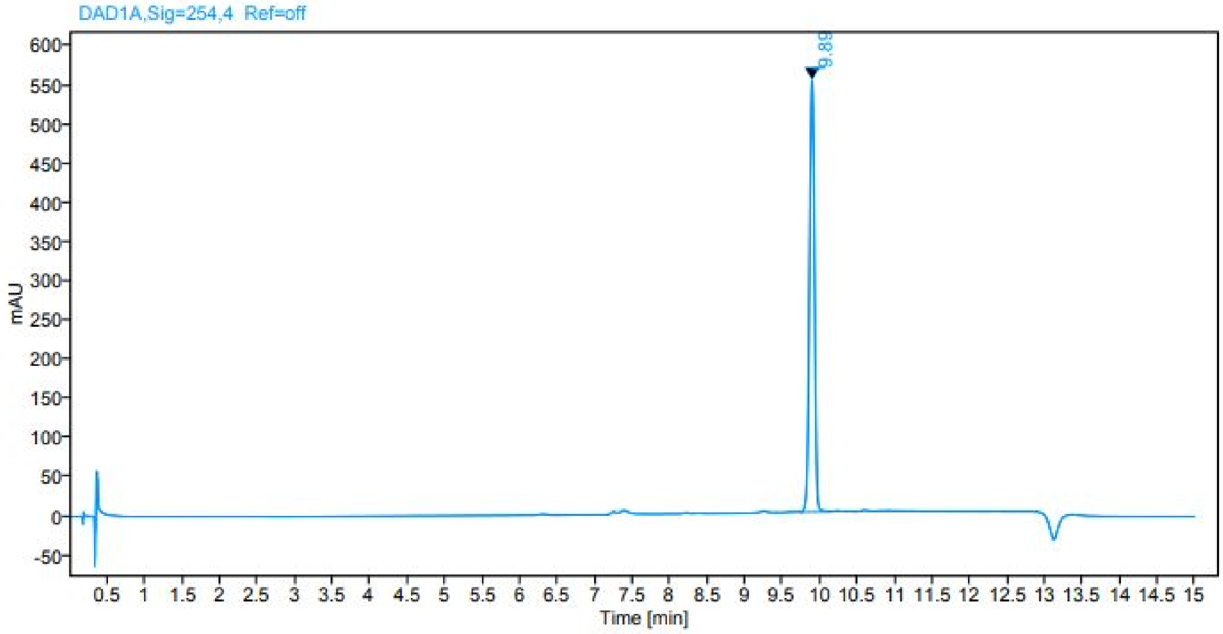

Signal: DAD1A,Sig=254,4 Ref=off

RT [min]	Type	Width [min]	Area	Height	Area%	Name
9.890	VV	0.4220	2530.0216	550.4482	100.0000	
		Sum	2530.0216			

X-Ray Data:

Crystal structures of HLQ-102, HLQ-105, and ELQ-300. The three-dimensional (3D) crystal structures of the antimalarial compounds **HLQ-102** and **HLQ-105** were elucidated by single-crystal X-ray diffraction analysis (Figures S1–S2). For comparative purposes, the 3D crystal structure of **ELQ-300**, the 3-substituted diaryl-ether isomer of **HLQ-102**, was also determined (Figure S3). The crystallographic analysis of **HLQ-105** revealed co-crystallization with a single water molecule, and the terminal phenyl ring of the biphenyl moiety was found to be disordered over two positions, corresponding to alternative conformational orientations (Figure S2). Within the molecule, there are hydrogen bonds between the oxygen of the quinolone ring and the water hydrogens with distances of 1.875 Å and 1.954 Å. The structure of **HLQ-102** exhibited no significant disorder. The structure of **ELQ-300** indicated that two molecules link with a hydrogen bonding between the oxygen of the keto group and the hydrogen of the NH group to form a dimeric assembly as the principal structural unit (Figure S3). The terminal OCF_3 substituent exhibited slight positional disorder, as reflected by elongated thermal ellipsoids for the atoms within this group. The central part in both molecules, N1, C1-C9, is planar within 0.034 Å (**HLQ-102**), 0.016 Å (**ELQ-300**), and 0.033 Å (**HLQ-105**). The orientations of the first phenyl ring (C12–C17) attached to the central quinolone core in **HLQ-102** and **HLQ-105** differ slightly. The dihedral angles between the mean plane of the quinolone ring and the first phenyl ring are 52.61(6)° and 56.65(8)° for **HLQ-102** and **HLQ-105**, respectively. In contrast, the corresponding phenyl ring in **ELQ-300** is more twisted relative to the quinolone core, with a dihedral angle of 69.05(9)°.

The orientation of the terminal phenyl groups in these molecules is significantly different. For **HLQ-102** and **ELQ-300**, the terminal phenyl ring of the diaryl-ether group is bent away from the central part of the molecule. Packing for **HLQ-102** shows a network of hydrogen bonds between the oxygen O1 of the quinolone to the adjacent N1-H1 and C6-H6 with distances of 1.894 Å and 2.418 Å, respectively. Similarly, packing for **HLQ-105** shows a network of hydrogen bonds between the oxygen O1 of the quinolone to the adjacent N1-H1 and C6-H6 with distances of 1.936 Å and 2.57 Å, respectively.

All of the bond distances found in **HLQ-102**, **ELQ-300**, and **HLQ-105** are inside the typical values for corresponding bonds.

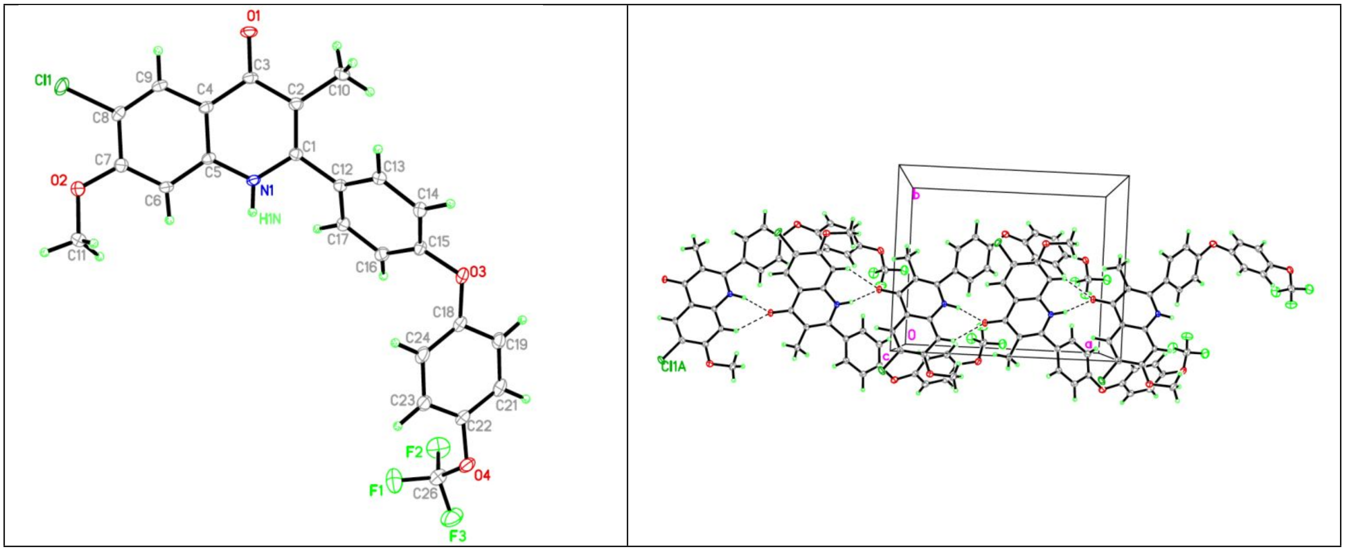

Figure S1: Left panel, ORTEP diagram of HLQ-102 in crystalline form. Ellipsoids are drawn at the 30% probability level. Right panel, ORTEP packing diagram showing the interaction between adjacent molecules in **HLQ-102** crystal. The dotted lines represent the hydrogen bonds between the C-H…O atoms.

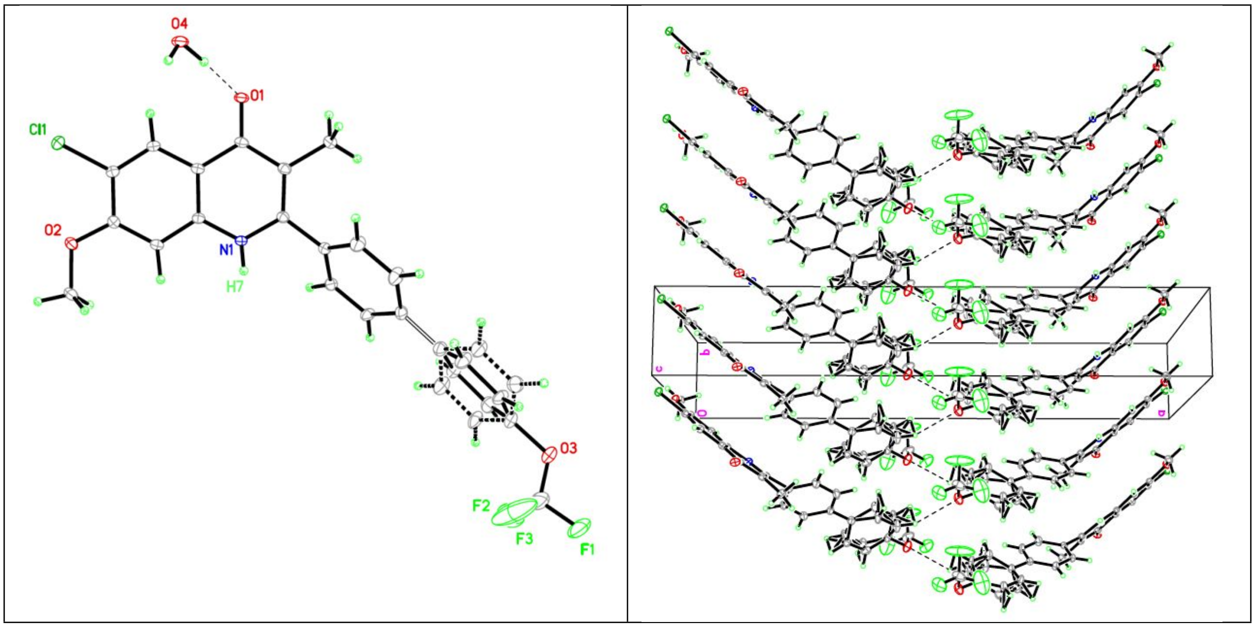

Figure S2: Left panel, ORTEP diagram of **HLQ-105** in crystalline form showing **HLQ-105** is co-crystallized with a molecule of water, and the terminal phenyl ring of the biphenyl is disordered over two positions corresponding to two possible orientations. Ellipsoids are drawn at the 30% probability level. Right panel, ORTEP packing diagram showing the interaction between adjacent molecules in the **HLQ-105** crystal. The dotted lines represent the hydrogen bonds between the H…O atoms.

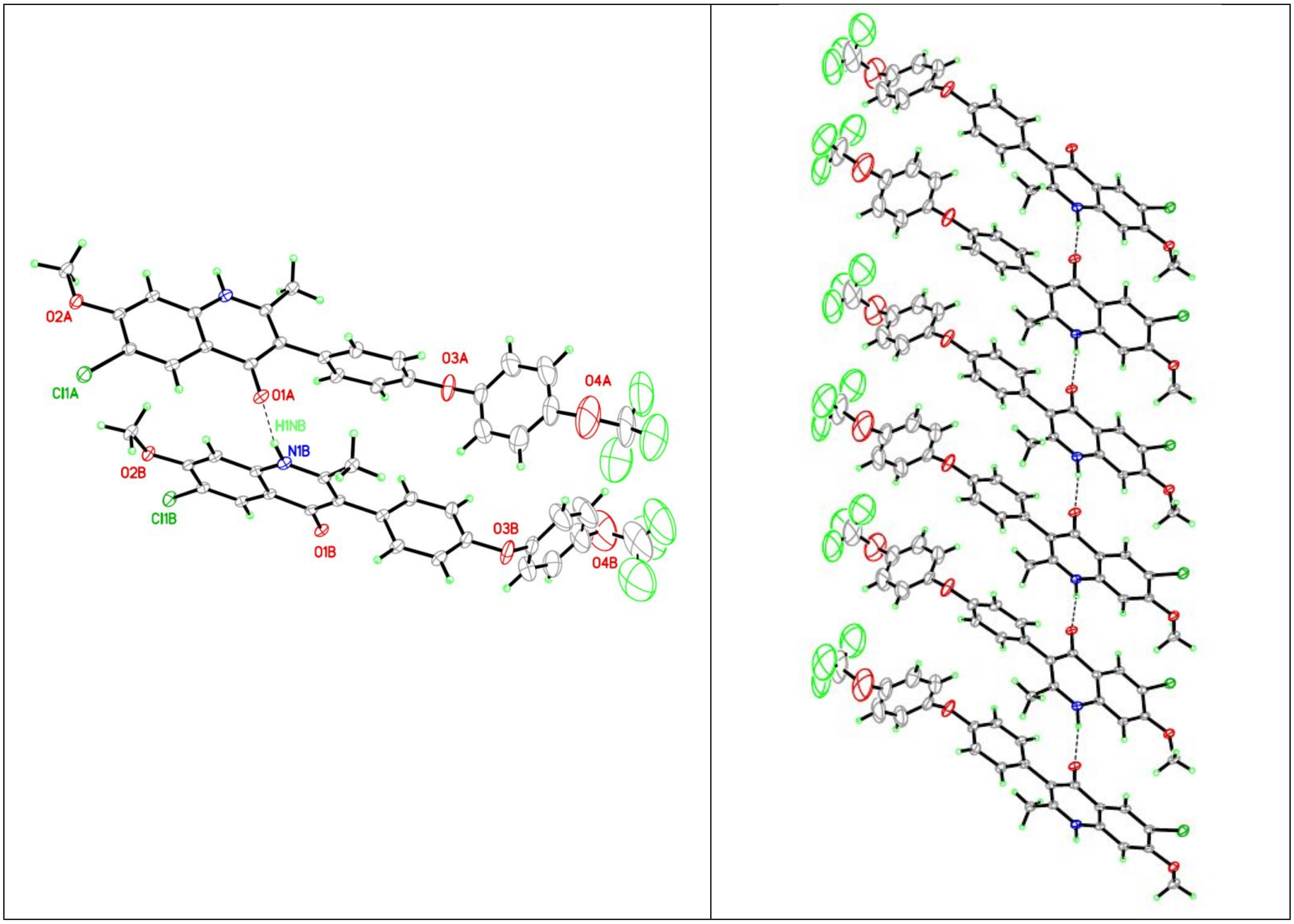

Figure S3: Left panel, ORTEP diagram of **ELQ-300** in crystalline form. Ellipsoids are drawn at the 30% probability level. Right panel, ORTEP packing diagram showing the interaction between adjacent molecules in **ELQ-300** crystal. The dotted lines represent the hydrogen bonds between the C-H…O atoms.

Crystallography Associated Methods and Analysis:

X-ray Crystallography. X-ray diffraction intensities for **HLQ-102**, **HLQ-105, and ELQ-300** were collected at 173 K on a Bruker Apex2 single crystal diffractometers using CuKα radiation, 1.54178 Å. Space groups were determined based on systematic absences. Absorption corrections were applied by SADABS[6]. Structures were solved by direct methods and Fourier techniques and refined on F^2 using full matrix least-squares procedures. All non-H atoms were refined with anisotropic thermal parameters. H atoms in **ELQ-300** were refined in calculated positions in a rigid group model, except the H atom at the N atom involved in H-bond which was refined based on the X-ray diffraction data. H atoms in **HLQ-102** were found on the residual density map and refined with isotropic thermal parameters. Thermal parameters of the C and O atoms in the terminal –OCF_3 groups are elongated indicating flexibility of this group. The RIGU option was used in the final refinement of **ELQ-300**. The structure of **HLQ-102** was determined in a non-centro-symmetrical space group of symmetry, Flack = 0.024(7). All calculations were performed by the Bruker SHELXL-2014/7 package[7]. H atoms in **HLQ-105** were refined in calculated positions in a rigid group model. All calculations were performed by the Bruker SHELXL-2014/7 package[7].

Crystallographic Data for **HLQ-102**: $C_{24}H_{17}ClF_3NO_4$, M = 475.83 g/mol, 0.13 x 0.10 x 0.05 mm, T = 173(2) K, Orthorhombic, space group *Pna*2, a = 13.0672(3) Å, b = 10.5685(3) Å, c = 15.5478(4) Å, V = 2147.16(10) Å^3, Z = 4, Dc = 1.472 Mg/m^3, μ(Cu) = 2.104 mm^{-1}, F(000) = 976, $2\theta_{max}$ = 133.38°, 25377 reflections, 3762 independent reflections [R_{int} = 0.0474], R1 = 0.0273, wR2 = 0.0685 and GOF = 1.044 for 3762 reflections (366 parameters) with

I>2σ(I), R1 = 0.0283, wR2 = 0.0691 and GOF = 1.044 for all reflections, the Flack = 0.024(7), max/min residual electron density +0.134/-0.172 e$Å^{-3}$.

Crystallographic Data for **ELQ-300**: $C_{24}H_{17}C1F_3NO_4$, M = 475.83 g/mol, 0.04 x 0.21 x 0.24 mm, T = 173(2) K, Monoclinic, space group *P2/c*, *a* = 34.8318(11) Å, *b* = 5.6822(2) Å, *c* = 10.9515(4) Å, *β* = 98.438(2)°, *V* = 2144.07(13) $Å^3$, *Z* = 4, *D*c = 1.474 Mg/m^3, *μ*(Cu) = 2.107 mm^{-1}, F(000) = 976, $2\theta_{max}$ = 133.34°, 11916 reflections, 3672 independent reflections [R_{int} = 0.0301], R1 = 0.0715, wR2 = 0.2131 and GOF = 1.046 for 3672 reflections (275 parameters) with I>2σ(I), R1 = 0.0817, wR2 = 0.2259 and GOF = 1.070 for all reflections, max/min residual electron density +0.789/-0.711 e$Å^{-3}$.

Crystallographic Data for **HLQ-105**: $C_{24}H_{17}ClF_3NO_3$, M = 459.85 g/mol, 0.21 x 0.02 x 0.02 mm, T = 213(2) K, Monoclinic, space group *P2/c*, *a* = 28.0402(10) Å, *b* = 4.5435(2) Å, *c* = 16.6366(6) Å, *β* = 90.667(2)°, *V* = 2119.37(14) $Å^3$, *Z* = 4, *D*c = 1.498 Mg/m^3, *μ*(Cu) = 2.131 mm^{-1}, F(000) = 984, $2\theta_{max}$ = 133.55°, 18385 reflections, 3752 independent reflections [R_{int} = 0.0705], R1 = 0.0577, wR2 = 0.1546 and GOF = 1.059 for 3752 reflections (379 parameters) with I>2σ(I), R1 = 0.0718, wR2 = 0.1659 and GOF = 1.059 for all reflections, max/min residual electron density +1.164/-0.777 e$Å^{-3}$.

Supplementary Tables:

Table S1. The GOLD docking scores of ELQ-300 and HLQ-102 are summarized below in the table (see associated publication for Figure and context.

Ligand	With Lipid	Without Lipid
**ELQ-300**	91.63	91.35
**HLQ-102**	93.95	83.10

Supplementary Figures:

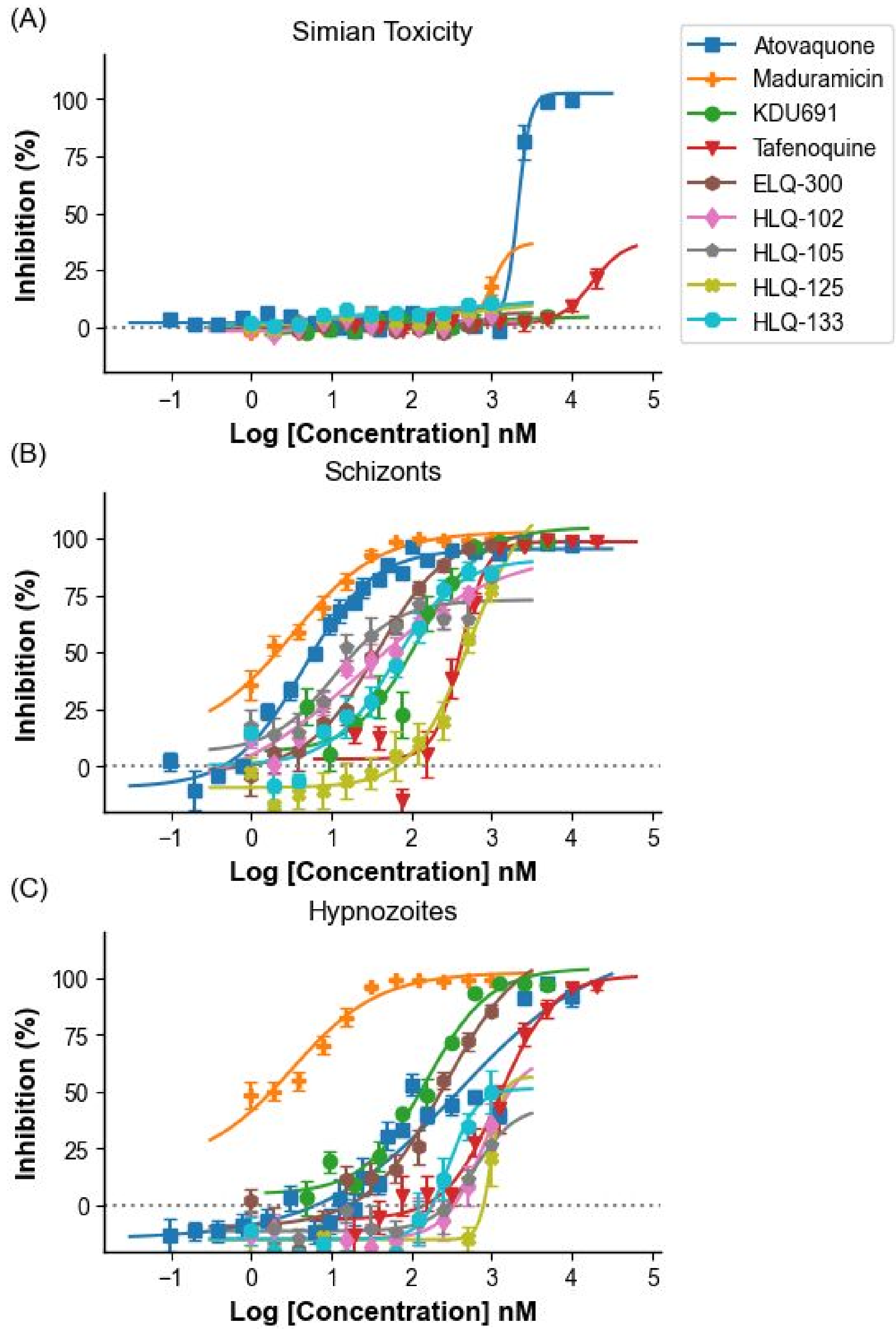

Figure S1. Dose–response prophylactic in vitro liver stage activity for selected HLQs and reference compounds against *P. cynomolgi* infections (schizont and quiescent forms) in non-human primate hepatocytes. Compounds were administered beginning 1 h after sporozoite invasion with repeated dosing through Day 2; parasite burden was evaluated on Day 8 by high-content imaging. (A) Hepatocyte toxicity, (B) Schizont inhibition, (C) Hypnozoite inhibition. Data are normalized to uninfected and vehicle controls; curves are representative of 3 independent experiments. Error bars represent SEM. Atovaquone, ELQ-300, maduramicin, KDU691, and tafenoquine are shown as antimalarial comparators. IC_{50} values are presented in the associated publication.

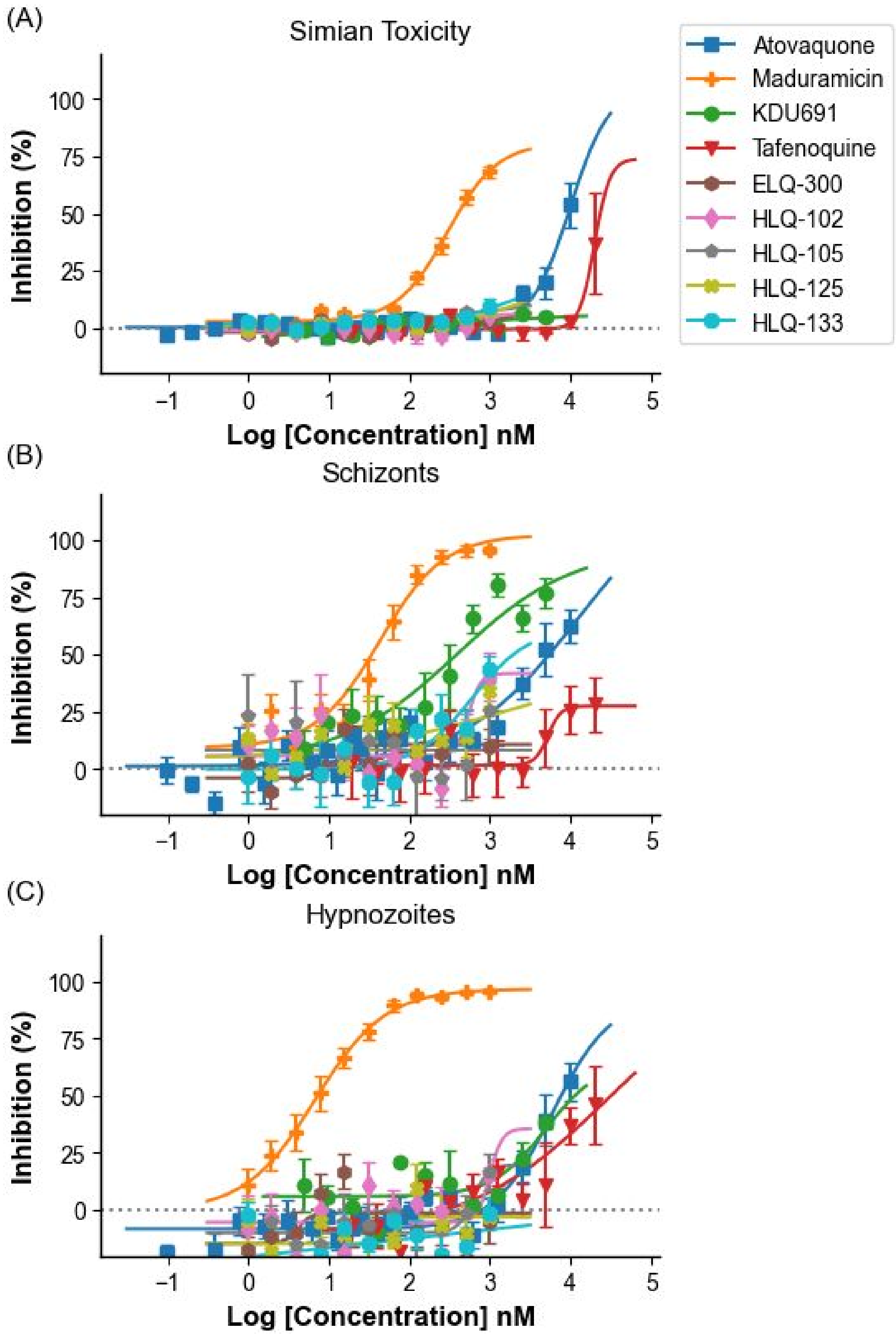

Figure S2. Dose–response radical cure in vitro liver stage activity for selected HLQs and reference compounds against *P. cynomolgi* infections (schizont and quiescent forms) in non-human primate hepatocytes. Compound treatment was initiated after establishment of infection (Days 4–7 post–invasion) with readout on Day 8 by high-content imaging. (A) Hepatocyte toxicity, (B) Schizont inhibition, (C) Hypnozoite inhibition. Data are normalized to uninfected and vehicle controls; curves are representative of 3 independent experiments. Error bars represent SEM. Atovaquone, ELQ-300, maduramicin, KDU691, and tafenoquine are shown as antimalarial comparators. IC_{50} values are presented in the associated publication.

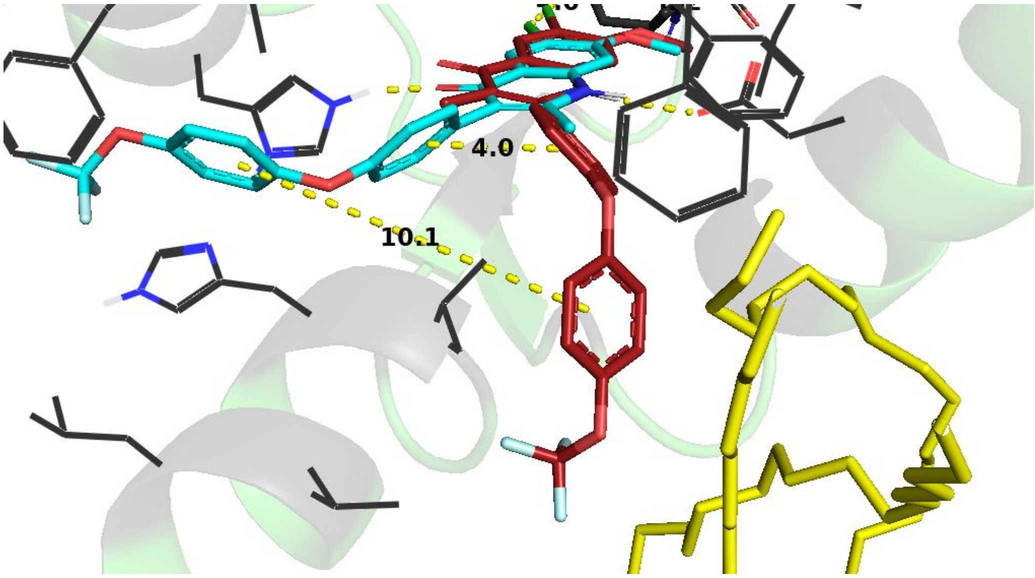

Figure S3. The distance in angstroms between the innermost ring of ELQ-300 and the innermost ring of HLQ-102 is 4Å, and the distance between the outermost rings of ELQ-300 and HLQ-102 is 10.1Å.

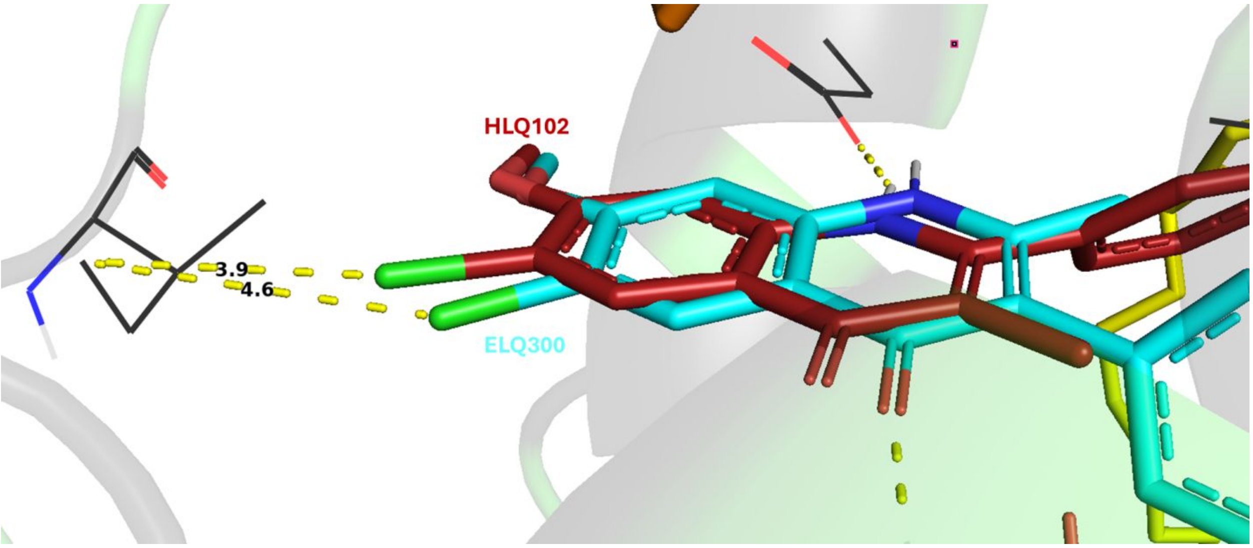

Figure S4. The distances between the chlorine atom on the drug and the I22 residue of the protein for the ELQ-300 docking and the HLQ-102 docking are 4.6 and 3.9 Å respectively for ELQ-300 and HLQ-102.

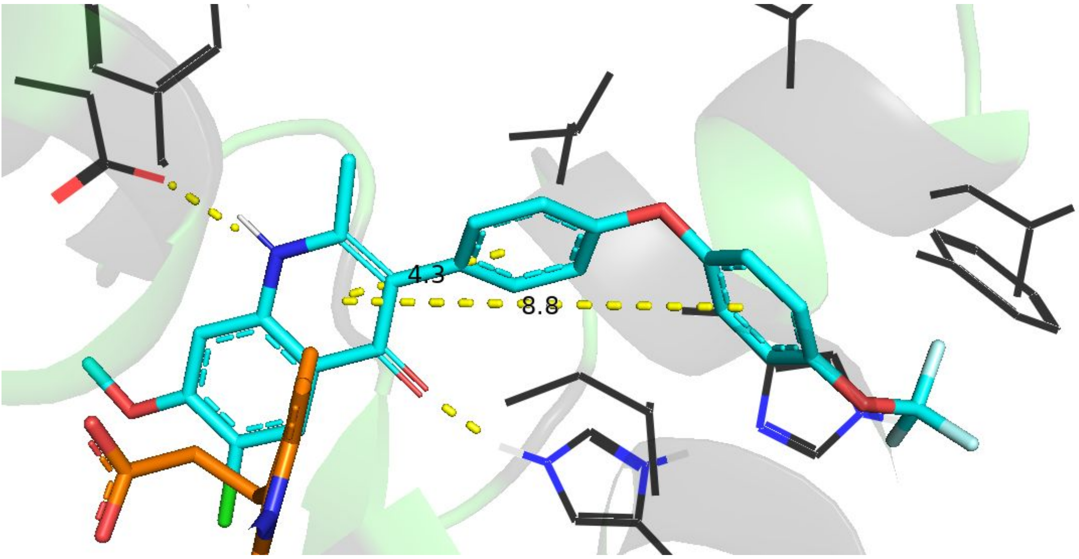

Figure S5. The distance between the quinolone ring and the outer ring in ELQ-300 is 8.8 Å, and the distance between the quinolone ring and the inner ring is 4.3 Å.

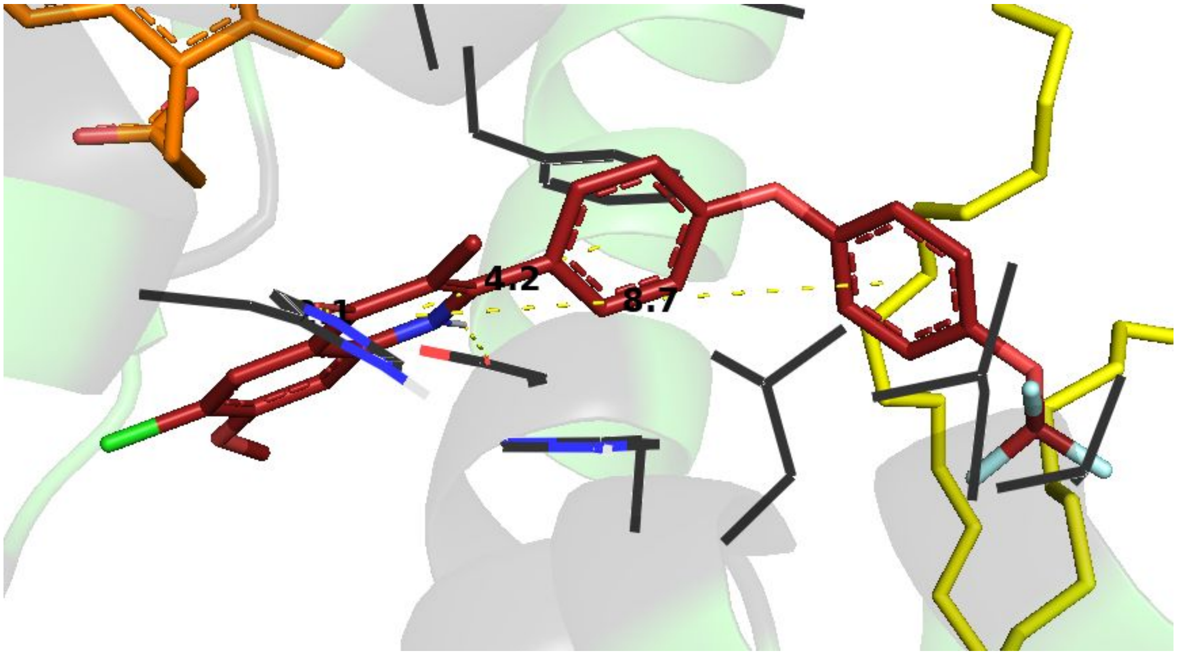

Figure S6. The distance between the quinolone ring and the outer ring in HLQ-102 is 8.7 Å, and the distance between the inner ring and the quinolone ring is 4.2 Å.

References:

(1) Armarego, W. L. F. C., Christina Li Lin. *Purification of Laboratory Chemicals, 7th Edition*; Butterworth-Heinemann, 2012.
(2) Szamosvári, D.; Reichle, V. F.; Jureschi, M.; Böttcher, T. Synthetic quinolone signal analogues inhibiting the virulence factor elastase of Pseudomonas aeruginosa. *Chemical Communications* **2016**, *52* (92), 13440-13443, 10.1039/C6CC06295D. DOI: 10.1039/C6CC06295D.
(3) Kocieński, P. J.; Pelotier, B.; Pons, J.-M.; Prideaux, H. Asymmetric syntheses of panclicins A–E via [2+2] cycloaddition of alkyl(trimethylsilyl)ketenes to a β-silyloxyaldehyde. *Journal of the Chemical Society, Perkin Transactions 1* **1998**, (8), 1373-1382, 10.1039/A800807H. DOI: 10.1039/A800807H.
(4) Reen, F. J.; Clarke, S. L.; Legendre, C.; McSweeney, C. M.; Eccles, K. S.; Lawrence, S. E.; O'Gara, F.; McGlacken, G. P. Structure–function analysis of the C-3 position in analogues of microbial behavioural modulators HHQ and PQS. *Organic & Biomolecular Chemistry* **2012**, *10* (44), 8903-8910, 10.1039/C2OB26823J. DOI: 10.1039/C2OB26823J.
(5) Pidathala, C.; Amewu, R.; Pacorel, B.; Nixon, G. L.; Gibbons, P.; Hong, W. D.; Leung, S. C.; Berry, N. G.; Sharma, R.; Stocks, P. A.; et al. Identification, design and biological evaluation of bisaryl quinolones targeting Plasmodium falciparum type II NADH:quinone oxidoreductase (PfNDH2). *J Med Chem* **2012**, *55* (5), 1831-1843. DOI: 10.1021/jm201179h.
(6) *Bruker/Siemens Area Detector Absorption Correction Program*; Bruker AXS: Madison, WI, 1998. (accessed.
(7) Sheldrick, G. M. Crystal structure refinement with SHELXL. *Acta Crystallogr C Struct Chem* **2015**, *71* (Pt 1), 3-8. DOI: 10.1107/S2053229614024218 From NLM PubMed-not-MEDLINE.
